# Promoted synthesis of spirooxindoles in the presence of chitosan containing an acidic ionic liquid bridge in aqueous medium

**DOI:** 10.1039/d5ra03286e

**Published:** 2025-11-24

**Authors:** Mohadeseh Amiri, Narges Seyedi, Farhad Shirini, Hassan Tajik

**Affiliations:** a Department of Orgnaic Chemistry, Faculty of Chemistry, University of Guilan 41335-19141 Iran shirini@guilan.ac.ir +98 131 3233262 +98 131 3233262

## Abstract

Chitosan (CS) containing an acidic ionic liquid bridge (CS-(M_3_SP)_2_-NH_2_·HSO_4_), was successfully prepared and utilized as a promoter in the one-pot multi-component synthesis of (±)-spiro[indoline-3,4′-pyrazolo[3,4-*b*]quinoline]dione and (±)-spiroindoline-pyrano[2,3-*c*]quinolone derivatives as important building blocks in pharmaceutical and medicinal chemistry. The catalytic efficiency was evaluated using a model reaction, and the yield was determined by HPLC analysis. This eco-friendly protocol offers several advantages, including low catalyst loading, excellent yields, short reaction times, and easy separation of the products. Notably, the catalyst was separated and reused several times without significant loss of its activity. In addition, the reaction was also investigated on a gram-scale, which showed that this method can be applied well at high amounts. In this study, the structural features and morphology of the catalyst were also thoroughly characterized using various techniques, including FT-IR, FESEM, EDX/EDX-map, XRD, TGA/DTG, and the Hammett test.

## Abstract 2

CS-(M_3_SP)_2_-NH_2_·HSO_4_, was successfully utilized as a promoter in the synthesis of (±)-spiro[indoline-3,4′-pyrazolo[3,4-*b*]quinoline]dione and (±)-spiroindoline-pyrano[2,3-*c*]quinolone derivatives.

## Graphical abstract

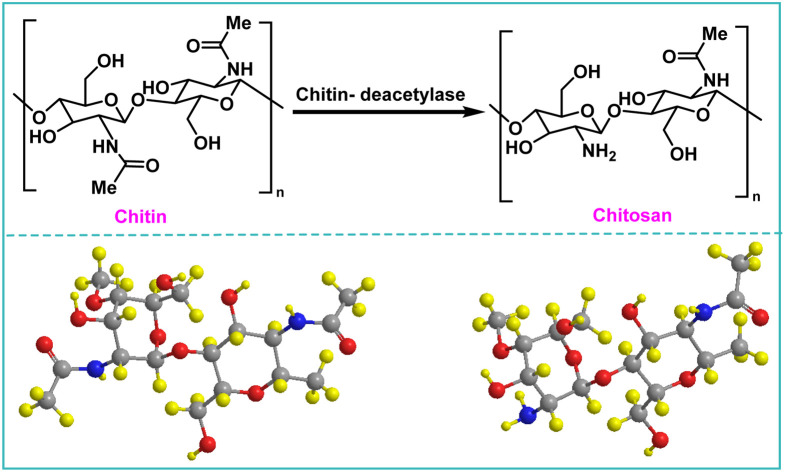

## Introduction

Catalysts play a crucial role in making chemical industries economically viable and environmentally sustainable.^1^ For example, over 75% of the industrial chemical reactions, spanning areas like polymers, pharmaceuticals, agrochemicals, and petrochemicals, rely on catalysts. On the other hand, 90% of new processes being developed today utilize catalytic methods.

Catalysis is generally classified as homogeneous, where the components are in the same phase (usually gas or liquid), or heterogeneous, where the reactants and catalyst are in two separate phases.^2,3^ Between these two, and due to the advantages of easy catalyst recovery and recyclability, simple experimental procedures, mild reaction conditions, and reduced chemical waste compared to liquid phase methods, heterogeneous ones are usually preferred.^4^ This class of catalysts plays an important role in achieving faster large-scale production and selective product formation in producing essential products in critical fields.^5–7^

Ionic liquids (ILs) are salts composed of bulky organic cations and inorganic or organic anions. They often exist as a molten salt, which typically melts below 100 °C, and can also be found as solids in certain cases.^8^ These compounds exhibit negligible vapor pressure and are non-flammable.^9^ By modifying the cationic or anionic components of an IL, its physical properties can be easily tuned. The incorporation of acidic or basic functional groups into the cation or anion of an IL results in the formation of various acidic and basic types of these compounds, leading to the emergence of new IL classes^10^ that exhibit superior activity and selectivity compared to free ionic liquids.^11^ Despite these promising tunable properties, free ionic liquids face practical challenges, including relatively high viscosity, limited thermal stability, and difficulties in product separation and catalyst recovery. These limitations restrict their widespread use, especially in large-scale industrial applications. To overcome these drawbacks while maintaining their desirable features, researchers have focused on supported ionic liquids (SILs)—a subclass of ILs immobilized on solid substrates^12,13^

Supported ionic liquids combine the advantages of ILs and solid supports, offering improved stability, easier recovery, and enhanced catalytic performance through synergistic effects. SILs have gained significant attention in both fundamental research and practical applications for these reasons, effectively addressing many of the limitations associated with traditional free ionic liquids.^14,15^

**Fig. 1:**
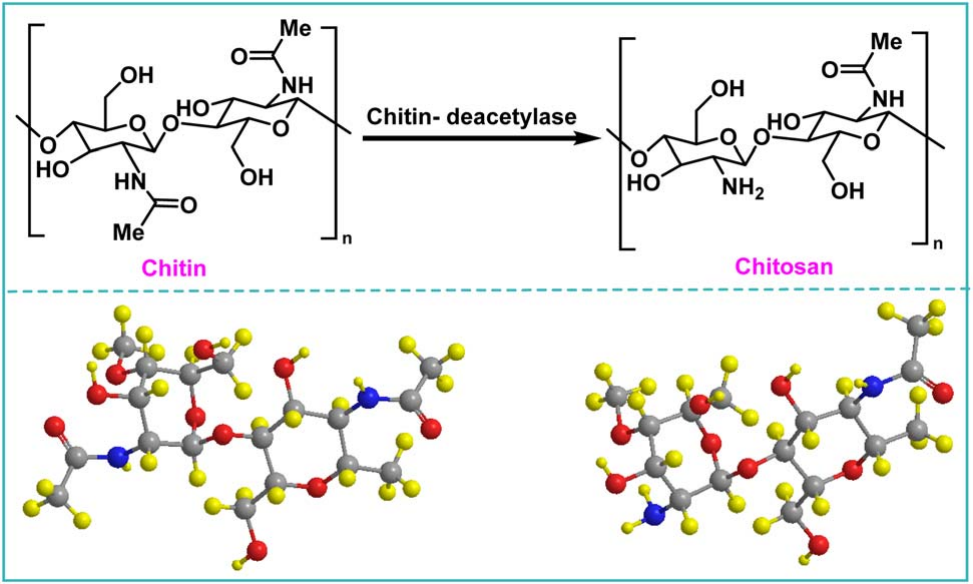
The structure of chitin and chitosan.

Chitosan, represented by the chemical formula (C_6_H_11_O_4_N)_*n*_, is a linear polysaccharide obtained from the deacetylation of chitin and scientifically known as β-(1,4)-2-amino-2-deoxy-d-glucopyranose and is the second most abundant natural biopolymer after cellulose (Fig. 1).

Important sources of chitosan (CS) are hard shell of crustaceans (such as crab and shrimp), the cell wall of fungi, and the exoskeleton of invertebrates and arthropods. This compound as a natural polymer has attracted much attention due to its non-toxicity, excellent absorption properties, ability to be degrade in nature, biocompatibility and cost-effectiveness.^15,16^ Researchers increasingly prefer chitosan due to its eco-friendly properties, high activity and ready availability. Functionalization of chitosan with an ionic liquid is one of the straightforward and effective techniques to modify its surface that have been investigated.^17,18^ The prepared chitosan-based catalysts not only show high activity and selectivity in catalytic processes, but also their green accessibility makes these compounds promising candidates for recyclable heterogeneous catalysis. As catalyst supports, they improve the efficiency of chemical reactions while reducing environmental impact.^19^

The title of multicomponent domino reactions (MDRs) are referred to as the third category of MCRs, which in them three or more different starting materials are easily mixed in one pot and demonstrate reactions under uniform conditions.^20^ These reactions, especially those which conducted in aqueous environments have become a useful tool for the synthesis of important chemical and biological compounds from the perspective of green chemistry, due to convergence, atomic economy, and other favorable properties.^21^

The indole nucleus is arguably the most recognized heterocycle, sharing a common and important feature in various natural products and pharmaceutical agents. Compounds carrying the indole moiety exhibit antibacterial and antifungal activities.^22^ Additionally, it has been reported that sharing the indole-3-carbon in the formation of spiro-oxindole derivatives greatly enhances their biological activity.^23^

**Fig. 2:**
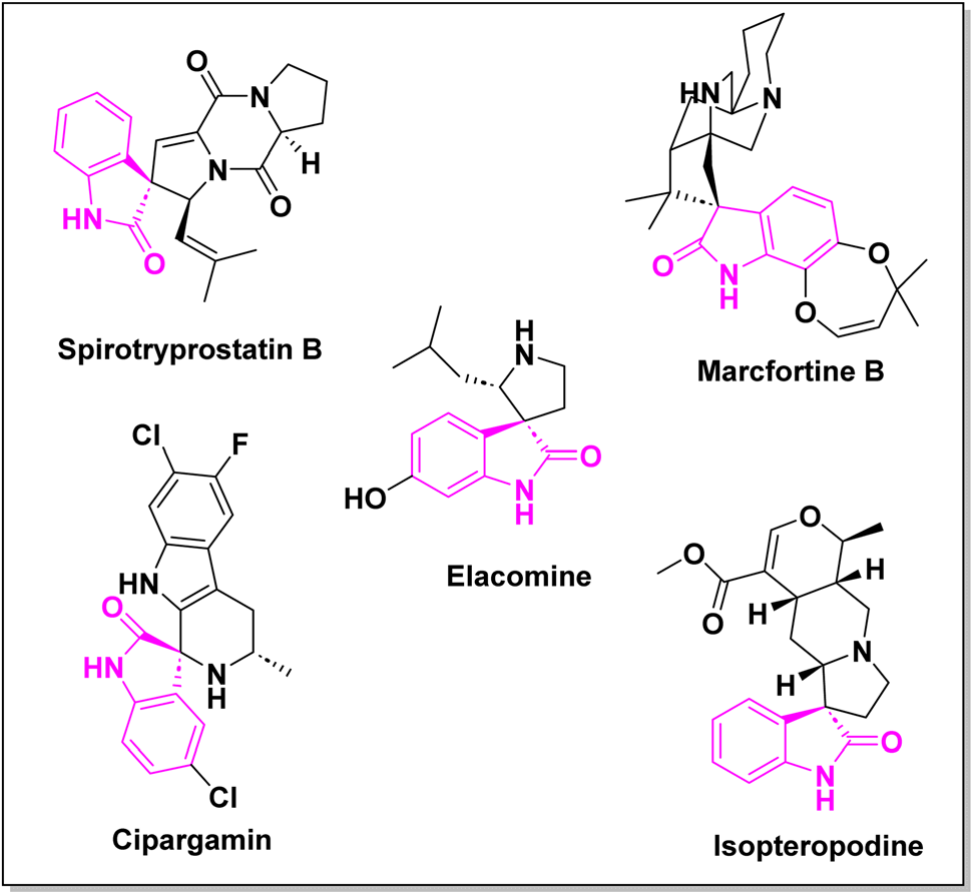
Structures of the representative spiro-oxindole alkaloids.

Spiro-oxindole systems are one of the isatin-based spiro-scaffolds found in various natural products such as phytochemicals in alkaloids, terpenoids, or lactones.^24^ Given their structural rigidity and three-dimensional architecture, spiro-oxindole derivatives often interact more selectively with biological targets, making them valuable scaffolds in drug design and discovery.^25,26^ Moreover, these compounds exhibit a wide range of medicinal and biological activities such as anti-mitotic, anti-cancer, anti-fungal, anti-parasitic, anti-microbial, and anti-malarial effects (Fig. 2).^27–29^ According to these points, isatins attract a lot of attention from organic chemists, because they can be widely used in the synthesis of spiro scaffolds from a variety of molecules directly or through 3-substituted 2-oxindole derivatives.^30^

Spiro-oxindoles are usually prepared *via* multicomponent reactions (MCRs) catalyzed by HAuCl_4_·3H_2_O,^31^ carbon–SO_3_H,^32^ [CMMIM][BF_4_],^33^ Cu(OAc)_2_.H_2_O,^34^ nano MgO,^35^ (SB-DBU)Cl,^36^ AHST-MNP,^37^ [Amb]*l*-prolinate,^38^ β-cyclodextrin (β-CD),^39^ papain,^40^*p*-TSA,^41^ tris-hydroxymethylaminomethane (THAM),^42^*etc.* Although all these reported systems have advantages, use of some of them is accompanied by restrictions such as harsh reaction conditions, long reaction times, use of volatile organic solvents and use of expensive metals. Hence, further research is still necessary to provide efficient, cost-effective and environmentally friendly catalytic systems for the synthesis of these heterocyclic compounds.

Herein and in continuation of our ongoing research program in aqueous organic synthesis and our work on the synthesis of indole derivatives,^43–45^ we wish to introduce a novel and potent acidic ionic liquid supported on chitosan (CS-(M_3_SP)_2_-NH_2_·HSO_4_) for accelerating the synthesis of spiro frameworks, including (±)-spiro[indoline-3,4′-pyrazolo[3,4-*b*]quinoline]dione and (±)-spiroindoline-pyrano[2,3-*c*]quinolone derivatives. This unique catalyst is easily prepared by synthesizing and consolidating bis-3-(trimethoxysilylpropyl)-ammonium hydrogen sulfate onto chitosan. We believe that this catalyst, with its distinctive features, has the potential to overcome some of the limitations observed in previously reported methods and to exhibit excellent catalytic performance.

## Experimental

All materials were obtained from Fluka, Merck, and Aldrich chemical companies and used without further purification. Chitosan (>97%) and bis[3-(trimethoxysilyl)propyl]amine (90%) were used in the catalyst preparation process. Isatins (isatin (98%), 4-chloroisatin (97%), and 4-bromoisatin (98%))/acenaphthylene-1,2-dione (95%), acenaphthoquinone (95%), malononitrile (99%), acidic hydrogen-containing compounds (1,3-cyclohexanedione (98%), dimedone (95%), barbituric acid (99%), 1,3-dimethylbarbituric acid (98%), thiobarbituric acid (>99%), and 4-hydroxycoumarin (98%)), and 3-methyl-5-amino-1*H*-pyrazole (98%) were used for the synthesis of the requested target molecules. The obtained products were characterized by comparing their physical data as well as FT-IR and NMR spectra with those of authentic references. The reaction progress and the purity of the substrates were monitored through thin-layer chromatography (TLC) using silica gel plates (SIL G/UV 254). Melting points were determined on an IA9100 electrothermal apparatus and are reported in degrees Celsius. FT-IR spectra were recorded using a Bruker VERTEX 70 (Germany) spectrometer, either for neat liquids or in combination with KBr pellets. The ^1^H and ^13^C NMR spectra were recorded using Bruker BioSpin GmbH (Germany) operating at 300, 400, and 500 MHz, equipped with standard probes and software. Spectra were obtained in DMSO-d_6_ using tetramethylsilane (TMS) as an internal standard. X-ray diffraction (XRD) analysis was performed on a Philips X-Pert diffractometer (Panalytical Company, Netherlands). Field emission scanning electron microscopy (FESEM) coupled with energy-dispersive X-ray spectroscopy (EDX) was conducted using a TESCAN MIRA3 (Czech Republic) instrument. Thermogravimetric analysis (TGA) was carried out at the polymer laboratory employing a Q600 thermal analyzer (TA Company, USA). The acidic strength of the catalyst was evaluated using a Cary 300 UV-Vis spectrophotometer (USA). The reaction progress and yields were monitored using a Knauer (Germany) HPLC system with a C18 column (250 mm × 4.6 mm, 5 µm particle size). The mobile phase consisted of methanol/acetonitrile/water (50/40/10, v/v/v), with a flow rate of 0.8 mL min^−1^. Detection was carried out at 254 nm. Yields (%) were calculated based on the increase in peak area of the product, compared with calibration curves obtained from external standards.

### Synthesis of bis-(3-trimethoxysilylpropyl)-amine-functionalized chitosan (CS-(M_3_SP)_2_-NH)

In a 100 mL round-bottom flask, chitosan (CS) (1 g) was mixed with bis[3-(trimethoxysilyl)propyl]amine (1 mmol, 0.341 g) in 30 mL of distilled ethanol for 72 hours under reflux conditions. The resulting precipitate was subsequently washed with diethyl ether (2 × 10 mL) and allowed to dry at room temperature, yielding the final product (CS-(M_3_SP)_2_-NH) as a cream-colored powder (1.279 g).

### Synthesis of bis-(3-trimethoxysilylpropyl)-ammonium hydrogen sulfate-functionalized chitosan (CS-(M_3_SP)_2_-NH_2_·HSO4)

**Scheme 1:**
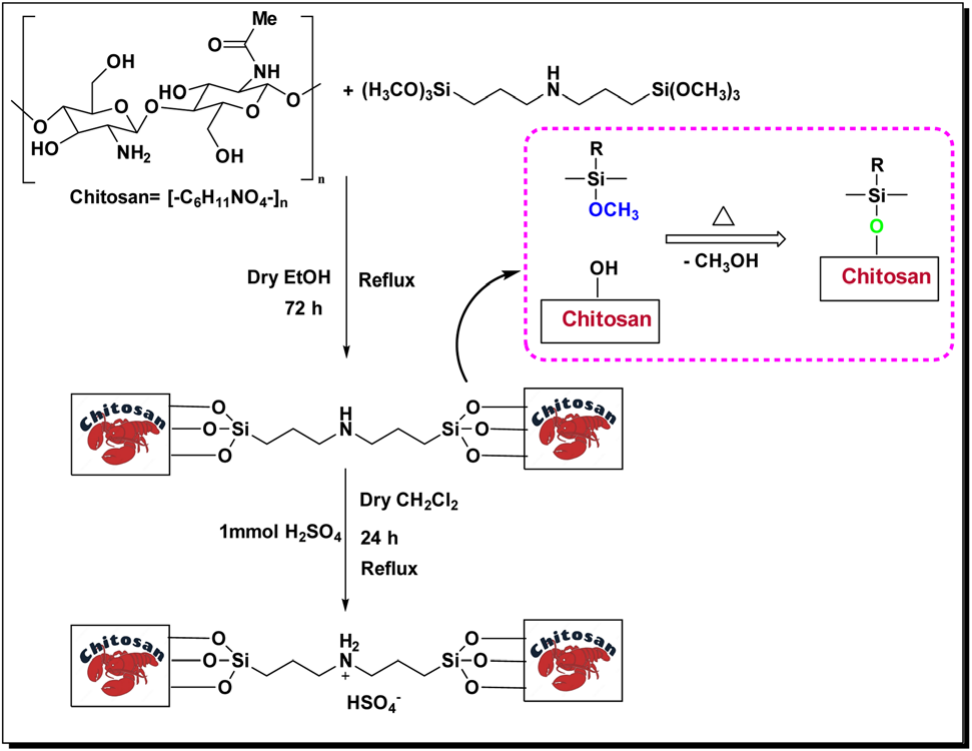
Synthesis of CS-(M_3_SP)_2_-NH_2_·HSO_4_.

In the subsequent step, CS-(M_3_SP)_2_-NH (1.279 g) was dispersed in 10 mL of dry dichloromethane, followed by the dropwise addition of H_2_SO_4_ (98%, 1 mmol, 0.098 g) under stirring in an ice bath (0 °C). The reaction mixture was refluxed for 24 hours to ensure the reaction reached completion. The resulting mixture was washed with diethyl ether (2 × 10 mL) and dried under vacuum to yield the final product, CS-(M_3_SP)_2_-NH_2_·HSO_4_ (1.343 g) (Scheme 1).^46^

### Synthesis of (±)-spiro[indoline-3,4′-pyrazolo[3,4-*b*]quinoline]dione derivatives

In a round-bottomed flask, a mixture of 3-methyl-5-amino-1*H*-pyrazole (1 mmol, 0.17 g), isatins (isatin, 4-chloroisatin, and 4-bromoisatin)/acenaphthylene-1,2-dione (1 mmol), acidic hydrogen-containing compounds (1,3-cyclohexanedione, dimedone, barbituric acid, 1,3-dimethylbarbituric acid, thiobarbituric acid, and 4-hydroxycoumarin) (1 mmol) and CS-(M_3_SP)_2_-NH_2_·HSO_4_ (20 mg), was prepared in water (3 mL). The resulting mixture was stirred for a suitable time in an oil bath at 80 °C by a magnetic stirrer. The progress of the reaction was monitored by TLC [*n*-hexane : ethyl acetate (4 : 7)]. After the completion of the reaction, water was evaporated and ethanol (10 mL) was added to the mixture and the catalyst was separated by simple filtration, dried at room temperature and stored to use in the next run in reusability test. In the next step, by the evaporation of the solvent from the filtrate and recrystallization of the precipitate in ethanol, the pure product was obtained in high yields. Under these reaction conditions, all synthesized compounds were obtained as a (±)-racemate, and no further chiral resolution was performed.

### Synthesis of (±)-spiroindoline-pyrano[2,3-*c*]quinolines derivatives

In a 10 mL flask, a mixture of isatins (isatin, 4-chloroisatin, and 4-bromoisatin)/acenaphthylene-1,2-dione (1 mmol), malononitrile (1.1 mmol, 0.07 g), acidic hydrogen-containing compounds (1,3-cyclohexanedione, dimedone, barbituric acid, thiobarbituric acid, and 4-hydroxycoumarin) (1 mmol), and CS-(M_3_SP)_2_-NH_2_·HSO_4_ (32 mg) in water (3 mL) was stirred under reflux conditions using a magnetic stirrer. The progress of the reaction was monitored by thin-layer chromatography (TLC) using *n*-hexane : ethyl acetate (3 : 7) as the eluent. Upon completion, the solvent was evaporated. Then 10 mL of ethanol was added to the residue, and the catalyst (CS-(M_3_SP)_2_-NH_2_·HSO_4_) was separated by simple filtration, dried at room temperature, and stored. After evaporation of the solvent from the filtrate, the pure product was obtained in high yields *via* recrystallization from ethanol. All compounds synthesized under the described conditions were obtained as (±)-racemic mixtures, and no enantiomeric separation was subsequently performed.

### Gram-scale synthesis of (±)-2-amino-7,7-dimethyl-2′,5-dioxo-5,6,7,8-tetrahydrospiro[chromene-4,3′-indoline]-3-carbonitrile

To synthesize the target compound on a gram scale, the reaction was conducted on a larger scale to assess the method's scalability and efficiency. Specifically, dimedone (5 mmol, 0.70 g), malononitrile (5.5 mmol, 0.36 g), and isatin (5 mmol, 0.73 g) were mixed in the presence of CS-(M_3_SP)_2_-NH_2_·HSO_4_ (5 mmol, 0.16 g) as a catalyst in water (10 mL). The mixture was stirred under reflux conditions for 8 minutes, with the process monitored by TLC. The reaction proceeded smoothly, yielding 94% of the desired product (1.68 g). These results aligned with those from smaller-scale tests, confirming the method's robustness and scalability. Additionally, using water as a solvent significantly reduced the environmental impact, and environmental assessments confirmed the method's sustainability for industrial-scale applications.

## Results and discussion

### Characterization of CS-(M_3_SP)_2_-NH_2_·HSO_4_

After the successful synthesis of CS-(M_3_SP)_2_-NH_2_·HSO_4_, it was comprehensively characterized using various analytical techniques, including Fourier transform infrared spectroscopy (FT-IR), field emission scanning electron microscopy (FESEM), energy-dispersive X-ray spectroscopy and mapping (EDX/EDX-map), X-ray diffraction (XRD), thermogravimetric and derivative thermogravimetric analyses (TGA/DTG), and the Hammett test.^46^

**Fig. 3:**
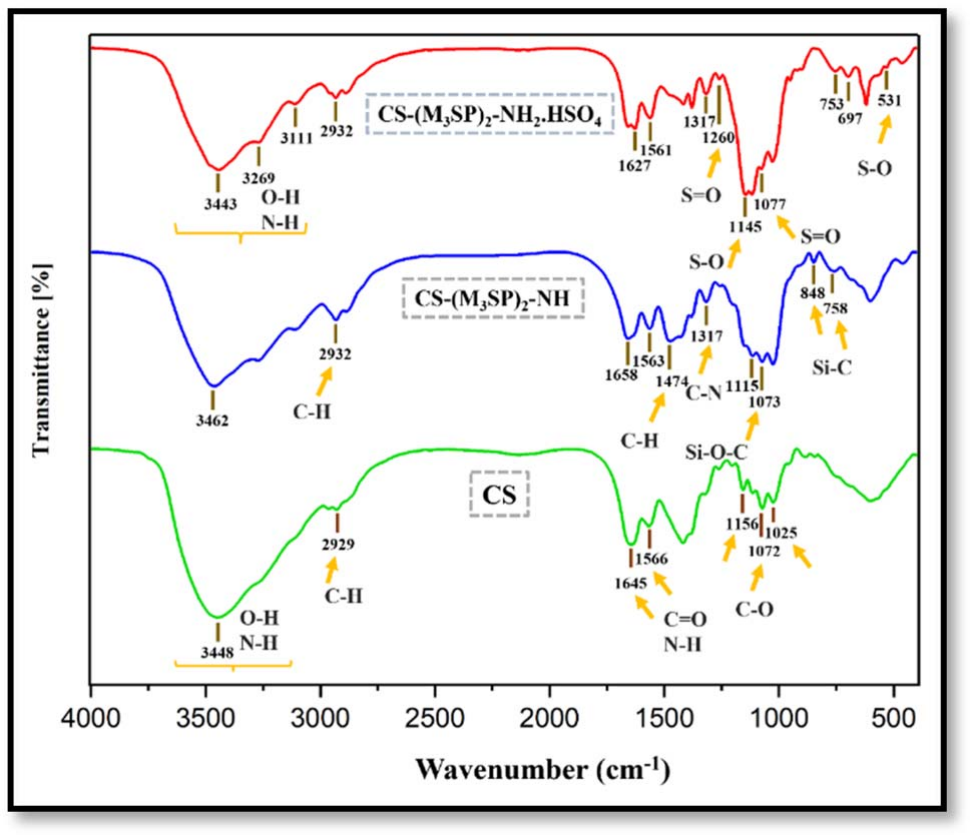
Comparison of the FT-IR spectra of CS, CS-(M_3_SP)_2_-NH and CS-(M_3_SP)_2_-NH_2_·HSO_4_.

FT-IR spectroscopy was employed to monitor the variations in functional groups during the synthesis of the catalyst. The infrared spectra of CS, CS-(M_3_SP)_2_-NH and CS-(M_3_SP)_2_-NH_2_·HSO_4_ are compared in Fig. 3. In the FT-IR spectrum of CS, the stretching vibrations of C–O bonds are detected at 1025 cm^−1^, 1072 cm^−1^ and 1156 cm^−1^. Moreover, the absorption bands observed at 1645 cm^−1^ and 1566 cm^−1^ correspond to the C=O stretching of residual acetyl amide groups and the N–H bending vibrations of amine groups, respectively. The band appearing in the region of 2929 cm^−1^ is assigned to the symmetric and asymmetric stretching vibrations of the C–H bond. In addition, the broad absorption in the 3200–3550 cm^−1^ region is attributed to the stretching vibrations of hydroxyl (–OH) and amine (–NH) functional groups.^47^

In the spectrum of CS-(M_3_SP)_2_-NH, absorption bands emerge at 758 cm^−1^ and 848 cm^−1^, corresponding to the Si–C stretching vibrations, while the band at 1073 cm^−1^ is assigned to Si–O–C stretching. Furthermore, the C–N stretching vibration appearing at 1317 cm^−1^ confirms the formation of chains on the chitosan surface. The peaks at 2932 cm^−1^ and 1474 cm^−1^ are associated with C–H stretching and bending vibrations, respectively. Also, a reduction in the intensity of the bands observed in the 2700–3000 cm^−1^ region is likely attributed to the restricted vibrations of the alkyl chains resulting from bond formation between the silane and chitosan groups.

In the spectrum of the manufactured catalyst, the peaks observed in the 1145 cm^−1^ and 531 cm^−1^ regions can be attributed to the asymmetric and symmetric S–O stretching vibrations, respectively. Also, the peaks at 1260 cm^−1^ and 1077 cm^−1^ are related to the asymmetric and symmetric stretching vibrations of the S=O bond, respectively. In addition, the stretching vibrations of the OH groups of SO_3_H overlapped with CS vibrations in the region of 3000–3600 cm^−1^.

**Fig. 4:**
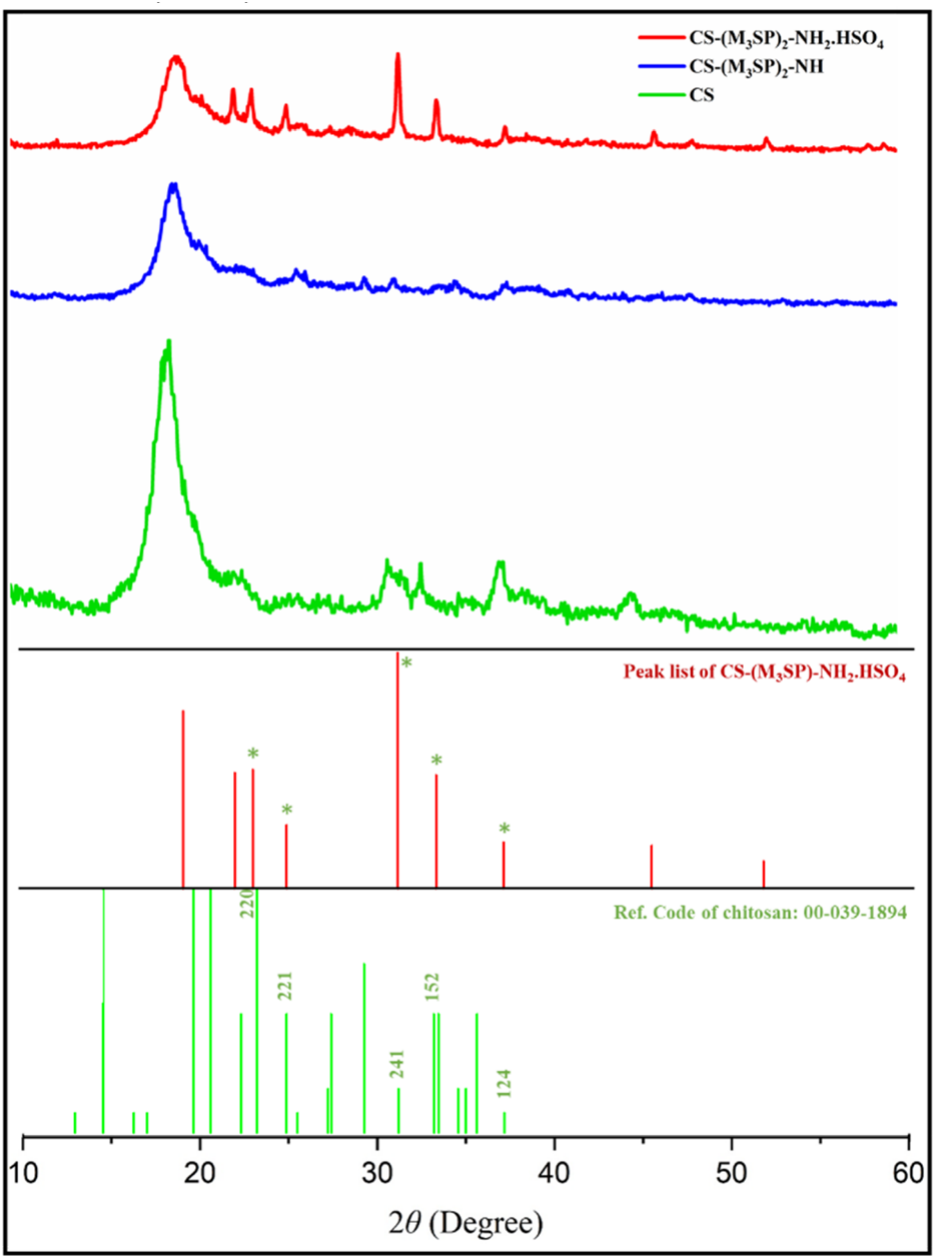
XRD patterns of CS, CS-(M_3_SP)_2_-NH and CS-(M_3_SP)_2_-NH_2_·HSO_4_.

X-ray diffraction (XRD) pattern was used to determine the structure and phase of CS, CS-(M_3_SP)_2_-NH and CS-(M_3_SP)_2_-NH_2_·HSO_4_ (Fig. 4). In this study, the XRD pattern related to chitosan, shows the main peak in the region of 2*θ* = 20.^48^ This peak is due to the presence of crystalline areas in the chitosan polymer chains and indicates its semi-crystalline structure.^49^

After the formation of the CS-(M_3_SP)_2_-NH, the intensity of this peak decreases, which indicates the interaction between the silane groups and chitosan polymer chains. After the addition of sulfuric acid, more changes occur in the crystal structure. Sulfuric acid can chemically react with the amine groups, leading to the structural changes resulting in the formation of new peaks.

The XRD pattern of CS-(M_3_SP)_2_-NH_2_·HSO_4_ exhibits five peaks at 2*θ* around 23.901, 25.577, 31.937, 33.929, and 37.934, corresponding to the Miller indices (220), (221), (241), (152), and (124) for the CS standard pattern (JCPDS: 00-039-1894). This confirms the presence of CS in the prepared ionic liquid. Additionally, other peaks can be attributed to the organic components in the catalyst's structure.

Also, the degree of crystallinity of CS-(M_3_SP)_2_-NH_2_·HSO_4_ was determined according to the following relationship.Crystallinity = (crystalline of area peaks)/(total area peaks)

The approximate size of crystalline particles was also calculated using Scherer's relation.*D* = (*kλ*)/(*β* cos *θ*)in the above relationship, *k* is the shape coefficient (0.9), *λ* is the wavelength of X-rays (0.154 in the device in question), *β* is the peak width at half the maximum height (in radians), *θ* is the diffraction angle of the peak (in radians) and *D* is the size. It is a crystalline particle (nanometer) that can be used to obtain the average size of the particles by using the peak that has the highest intensity. Based on the calculations, the average particle size in CS-(M_3_SP)_2_-NH_2_·HSO_4_ was 29.3 nm.

**Fig. 5:**
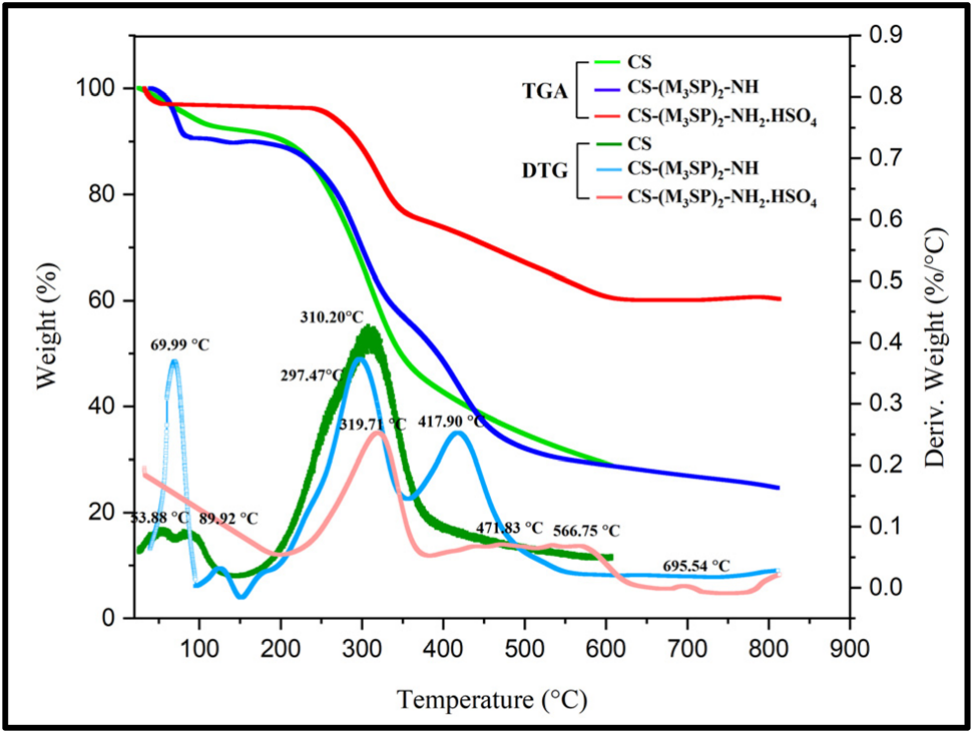
TGA and DTG curves of CS, CS-(M_3_SP)_2_-NH and CS-(M_3_SP)_2_-NH_2_·HSO_4_.

TGA and DTG analysis were used to compare the thermal stability of CS-(M_3_SP)_2_-NH_2_·HSO_4_ with CS and CS-(M_3_SP)_2_-NH (Fig. 5). In the first stage of the CS curve, the initial weight loss at temperatures of 53.88 and 89.92 °C by the amount of 7.84 w/w% is attributed to the loss of water absorbed by hydrogen bonds on the polymer substrate. The amounts of loss of water for CS-(M_3_SP)_2_-NH and CS-(M_3_SP)_2_-NH_2_·HSO_4_ under the same conditions are 9.39 w/w% (at 69.99 °C) and 1.24 w/w% (at 89.92 °C), respectively. The higher amounts of the absorbed water on these two reagents can be related to their higher hydrophilicity resulting from the appearance of groups capable to form hydrogen bonding on the surface of the modified chitosan. The second stage of weight loss for CS is about 44.45% by weight occurring at a temperature of 310.20 °C. In the curves related to CS-(M_3_SP)_2_-NH and CS-(M_3_SP)_2_-NH_2_·HSO_4_, the main weight loss occurs in two stages. In the curve of CS-(M_3_SP)_2_-NH, this weight loss occurs at 297.47 and 417.90 °C, equal to 28.15% and 23.71 w/w% by weight, respectively. In the curve of CS-(M_3_SP)_2_-NH_2_·HSO_4_, the weight loss at temperatures of 319.71 and 566.75 °C occurs at 19.44 and 15.82 w/w%, respectively, due to the formation of stronger bonds and more stable structures, leading to less thermal degradation which is observed.

The main weight loss occurs for CS around 300 °C and for CS-(M_3_SP)_2_-NH and CS-(M_3_SP)_2_-NH_2_·HSO_4_ at slightly higher temperatures, indicating their greater thermal stability. These results show the effect of chemical composition and interactions on the stability and thermal behavior of the synthesized catalyst.^46^

**Fig. 6:**
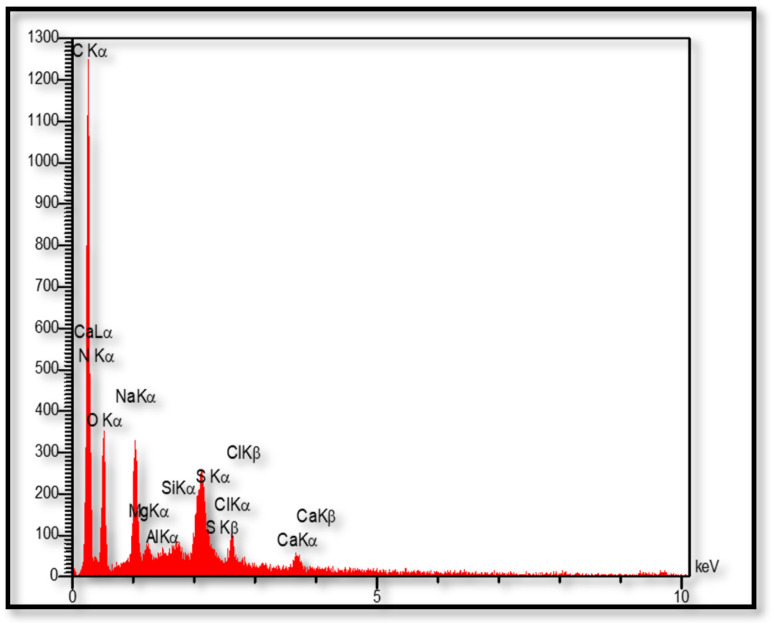
EDX spectrum of CS-(M_3_SP)_2_-NH_2_·HSO_4_.

Energy diffraction X-ray spectroscopy (EDX) is used as an analytical technique for the structural analysis of CS-(M_3_SP)_2_-NH_2_·HSO_4_. The results of this analysis show the presence of all the expected elements including C, N, Si, O and S in the structure of the prepared reagent. In addition, the elements Na, Mg, Ca, Al, and Cl detected in EDX are related to the natural chitosan substrate (Fig. 6).^50^

**Fig. 7:**
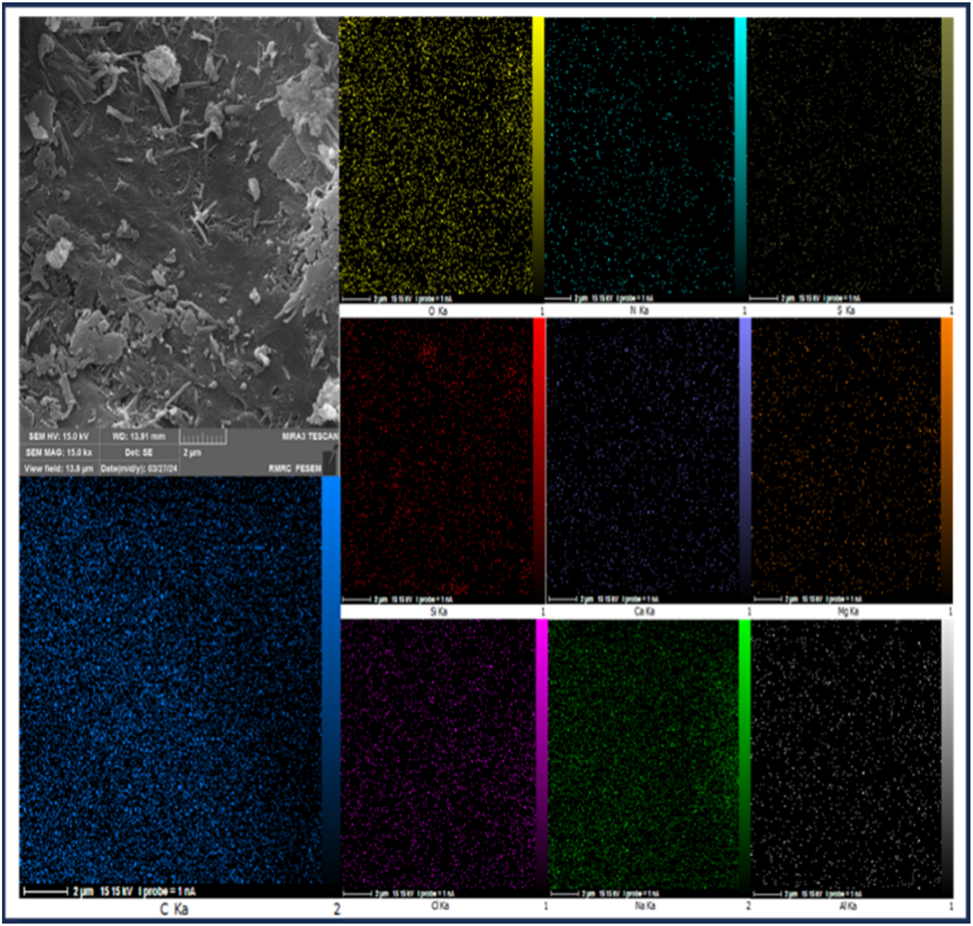
EDX-map analysis of CS-(M_3_SP)_2_-NH_2_·HSO_4_.

In addition to X-ray energy diffraction (EDX) analysis, X-ray mapping analysis (EDX-map) has also been used as a powerful tool to determine the distribution of elements in the catalyst. The images obtained from the X-ray mapping analysis of the synthesized catalyst (CS-(M_3_SP)_2_-NH_2_·HSO_4_) show a uniform distribution of C, N, Si, O, S, Na, Mg, Ca, Al, and Cl elements in its structure, reflecting the interaction and the high homogeneity of the components (Fig. 7).

**Fig. 8:**
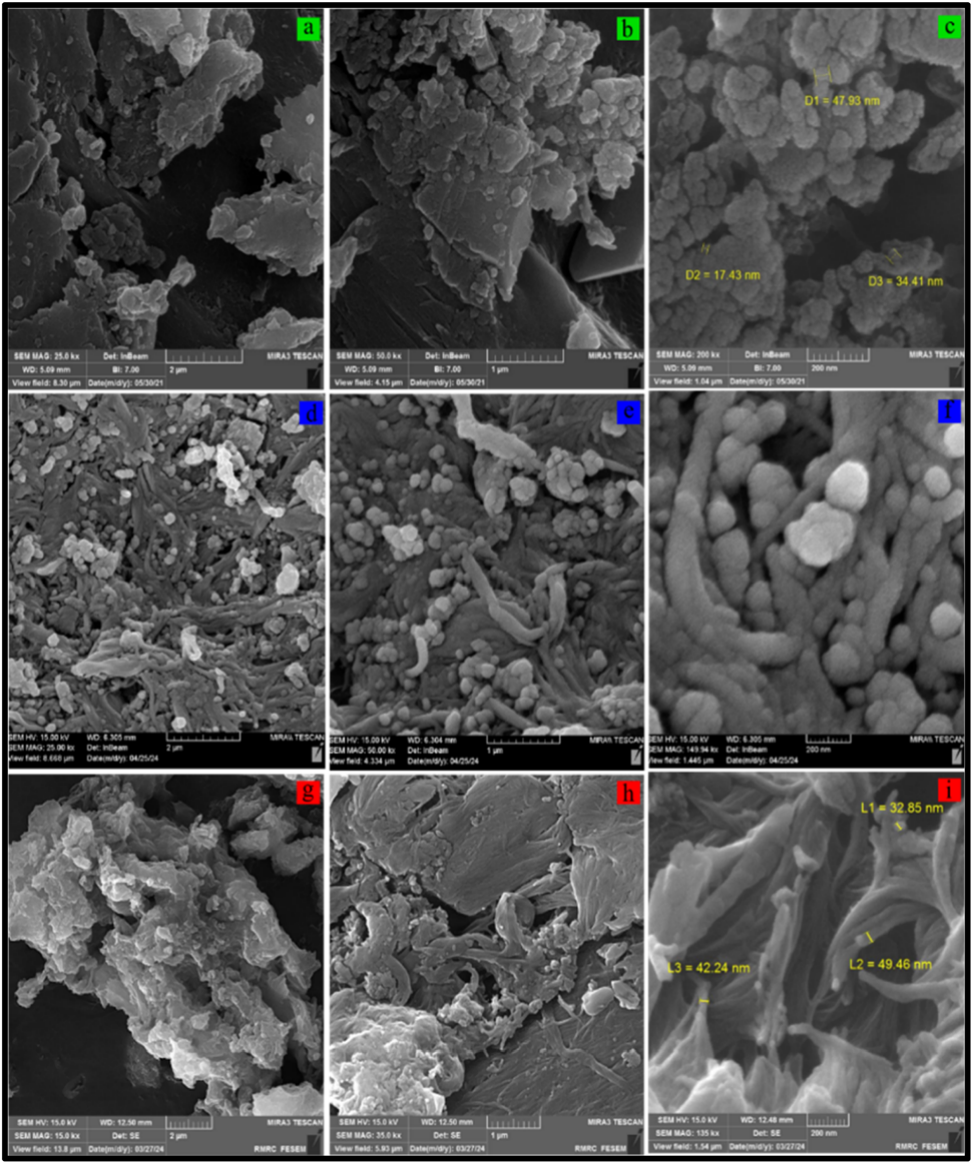
Comparison of the FESEM images of CS (a–c), CS-(M_3_SP)_2_-NH (d–f) and CS-(M_3_SP)_2_-NH_2_·HSO_4_ (g–i).

FESEM analysis was used to investigate the size, surface characteristics and morphology of CS, CS-(M_3_SP)_2_-NH and CS-(M_3_SP)_2_-NH_2_·HSO_4_ (Fig. 8). FESEM analysis reveals that CS exhibits a compact and uniform surface without significant three-dimensional features, consistent with its polymeric structure. In contrast, the modified catalyst (CS-(M_3_SP)_2_-NH_2_·HSO_4_) displays a more pronounced three-dimensional morphology compared to both pure CS and the intermediate CS-(M_3_SP)_2_-NH. This enhanced 3D architecture likely results from the two-step modification process, which introduces robust hydrogen-bonded crosslinking networks.^51–53^

Measuring the acidity of ionic liquids is critical in many applications. Hammett acidity method is an efficient method to evaluate the acid strength of a compound in organic solvents using UV-vis technique.

The Hammett function is defined as: *H*_0_ = pK(I)_aq_ + log([*I*]_s_/[IH^+^]_s_ where pK(*I*)_aq_ is the p*K*_a_ value of the aqueous solution and [IH^+^]_s_ and [*I*]_s_ are the molar concentrations of the protonated and deprotonated forms in the solvent, respectively.

According to the Lambert–Beer's law, the value of [*I*]_s_/[IH^+^]_s_ can be determined and calculated through the UV-visible spectrum.

**Fig. 9:**
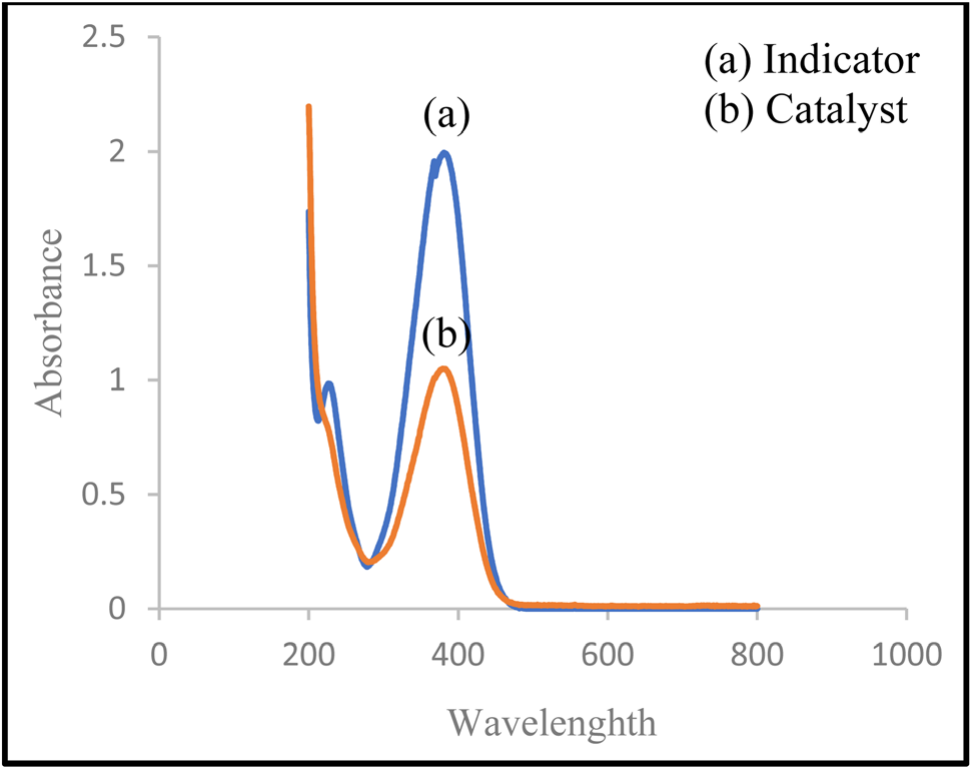
Absorption spectra of 4-nitroaniline before (blue) and after (orange) addition of the CS-(M_3_SP)_2_-NH_2_·HSO_4_ in water.

In the present experiment, 4-nitroaniline (pK(*I*)_aq_ = 0.99) was employed as the basic indicator, while distilled water served as the solvent. The deprotonated form of the indicator (10 mL, concentration = 1.44 × 10^−4^ mol L^−1^) exhibited a maximum absorbance at 383 nm in distilled water at 25 °C. Upon addition of CS-(M_3_SP)_2_-NH_2_·HSO_4_ (10 mg) to the indicator solution, a decrease in the absorbance intensity of the deprotonated form of the indicator was observed, implying partial conversion of the indicator to [HI^+^]_s_. The obtained data are summarized in Table 1, confirming the acid strength of CS-(M_3_SP)_2_-NH_2_·HSO_4_ (Fig. 9).

**Table 1:** Calculation of the Hammett acidity function (*H*_0_) for CS-(M_3_SP)_2_-NH_2_·HSO_4_^a^

Entry	Catalyst	*A*_max_	[*I*]_s_%	[IH^+^]_s_%	H_0_
1	—	1.9916	100	0	—
2	CS-(M_3_SP)_2_-NH_2_·HSO_4_	1.0485	52.6	47.4	1.035

a Condition for UV-visible spectrum measurement: solvent: H_2_O, indicator: 4-nitroaniline (pK(I)aq = 0.99), 1.44 × 10^−4^ mol L^−1^ (10 mL); catalyst: CS-(M_3_SP)_2_-NH_2_·HSO4 (10 mg), 25 °C.

### Catalytic activity

After the successful identification of CS-(M_3_SP)_2_-NH_2_·HSO_4_, we aimed to investigate its catalytic performance in the synthesis of spiro-oxindole products, as a process that could be accelerated in the presence of acidic reagents, and to address the limitations associated with the previously reported methods for the synthesis of these compounds.

To optimize the reaction conditions for the preparation of the specified target molecules, the influence of CS-(M_3_SP)_2_-NH_2_·HSO_4_ on the reaction of isatin (1 mmol), 3-methyl-5-amino-1*H*-pyrazole (1 mmol), and 1,3-cyclohexanedione (1 mmol) under the effect of various conditions, including different temperatures, the presence or absence of solvent, and amounts of the catalyst was checked. Yield values were determined by HPLC analysis. The results obtained are shown in Table 2.

**Table 2:** Optimization of the reaction conditions in the synthesis of (±)-3′-methyl-1′-phenyl-1′,7′,8′,9′-tetrahydrospiro[indoline-3,4′-pyrazolo[3,4-*b*]quinoline]-2,5′(6′*H*)-dione^a^

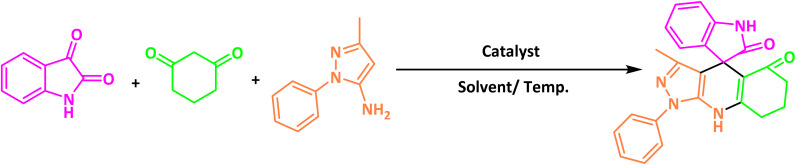					
Entry	Catalyst (mg)	Solvent	Temp. (^°^C)	Time (min.)	Yield^b^ (%)
1	20	Water	Reflux	7	96
2	20	Water	80	5	98
3	40	Water	80	5	97
4	20	Ethanol	Reflux	60	92
5	20	Water/ethanol (1 : 1)	Reflux	20	93
6	20	Acetonitrile	Reflux	45	64
7	20	Solvent-free	120	16	94
8	20	Solvent-free	100	16	93

a Reaction conditions: isatin (1 mmol), 3-methyl-5-amino-1*H*-pyrazole (1 mmol), 1,3-cyclohexanedione (1 mmol), solvent (3 mL), catalyst.

b Yield values were determined by HPLC analysis using the product as an external standard.

**Scheme 2:**
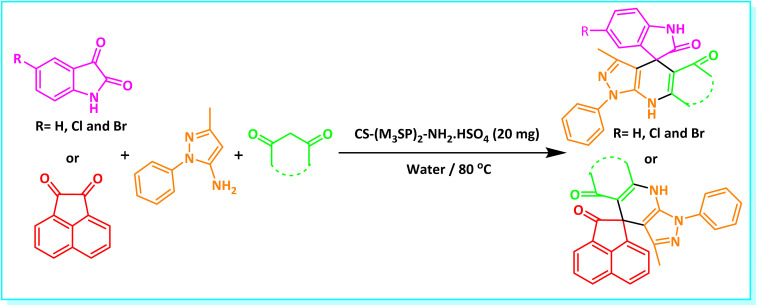
Synthesis of (±)-spiro-indoline-3,4′-pyrazolo[3,4-*b*]-pyridines in the presence of CS-(M_3_SP)_2_-NH_2_·HSO_4_.

The data in Table 2 clarify that the best results for this reaction were obtained by performing the model reaction in the presence of 20 mg of the catalyst in the absence of solvent at 80 °C (Table 2, entry 2) (Scheme 2). It should be noted that, as mentioned before this reaction was also carried out in other experimental conditions, involving different solvents, varying catalyst amounts, and temperatures. However, these conditions led to longer reaction times compared to the optimal one (Table 2, entries 1 and 3–8).

After confirming that CS-(M_3_SP)_2_-NH_2_·HSO_4_ could effectively accelerate the model reaction, various substituted isatins and/or acenaphthylene-1,2-dione, 3-methyl-5-amino-pyrazoles, and various acidic hydrogen containing compounds (1,3-cyclohexanedione, 4-hydroxycoumarin, dimedone, barbituric acid, 1,3-dimethylbarbituric acid, and thiobarbituric acid) were examined as substrates to explore the scope of this protocol (Table 3) (**1a–1o**). The results showed that all the studied reactions produced the requested target molecules during short reaction times with high yields.

**Table 3:** Synthesis of (±)-spiro-indoline-3,4′-pyrazolo[3,4-*b*]-pyridine derivatives using CS-(M_3_SP)_2_-NH_2_·HSO_4_ as an ionic heterogeneous catalyst^a^

Entry	Carbonyl compound	Acidic hydrogen containing compound	Product	Time (min.)	Yield^b^ (%)	M. P. (°C)	
						Found	Reported ^Ref.^
1	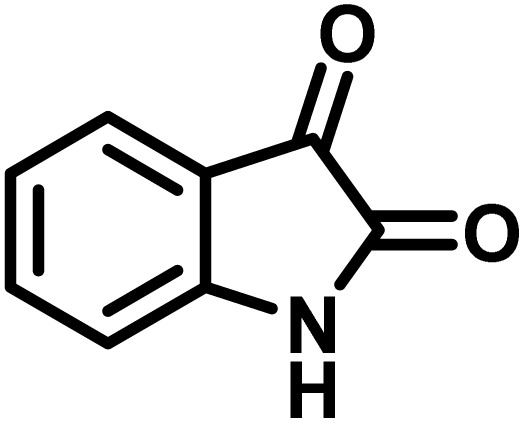	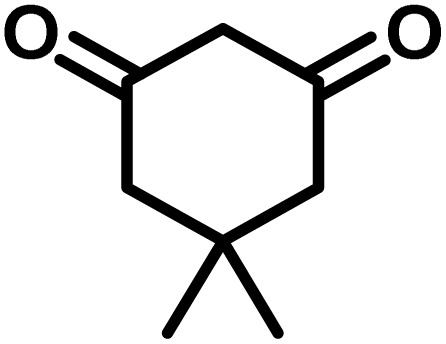	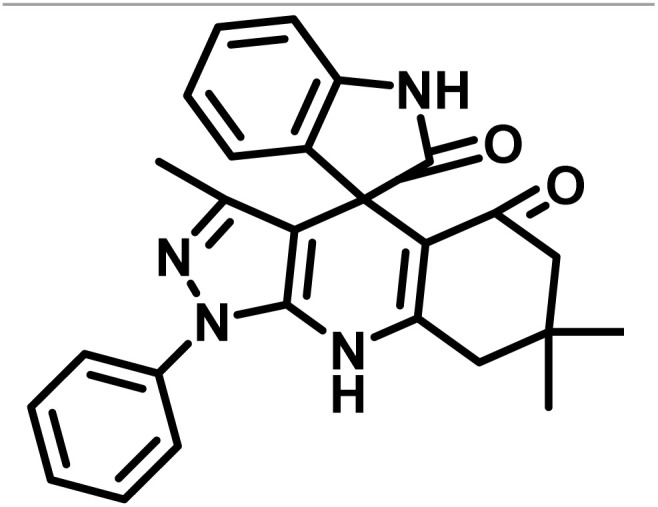	5	96	240–242	241–243 (ref. 40)
2	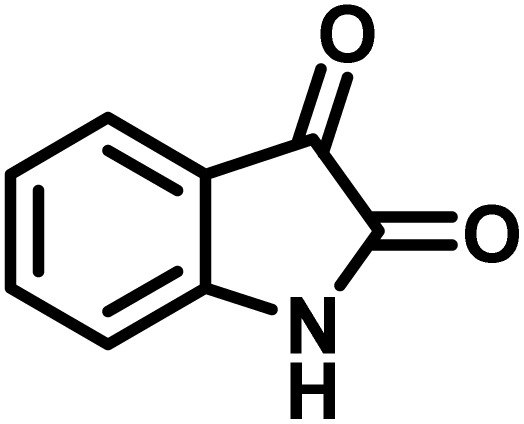	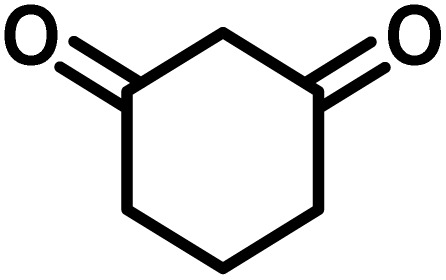	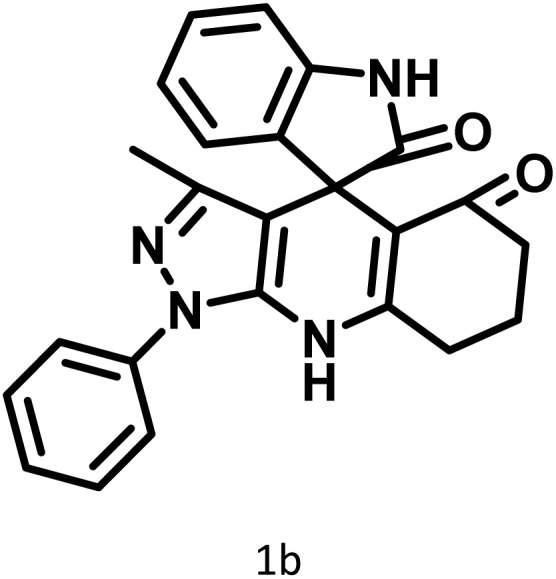	4	97	298–300	300 (ref. 21)
3	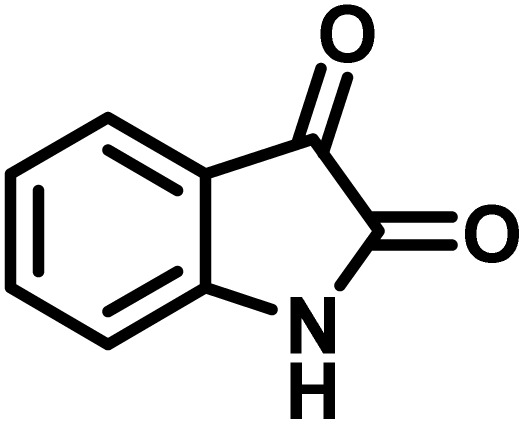	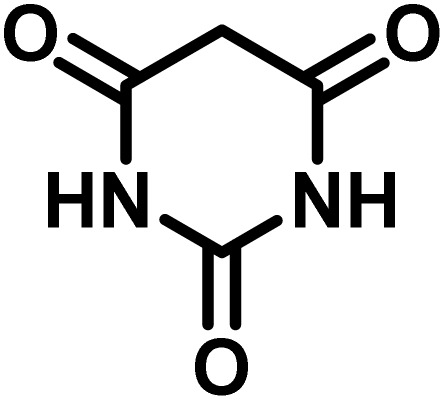	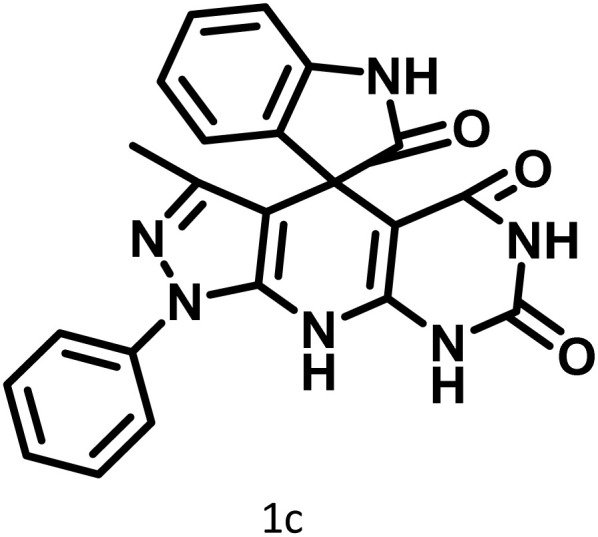	6	97	>300	300 (ref. 21)
4	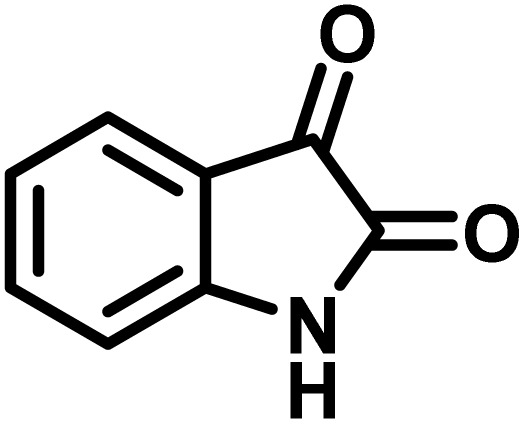	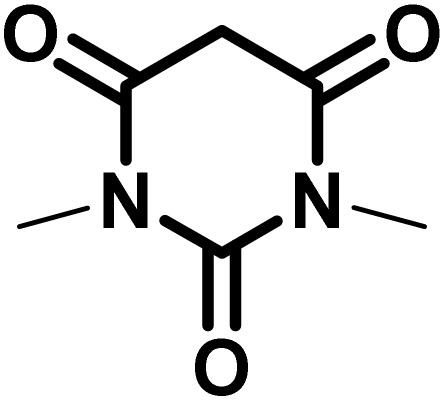	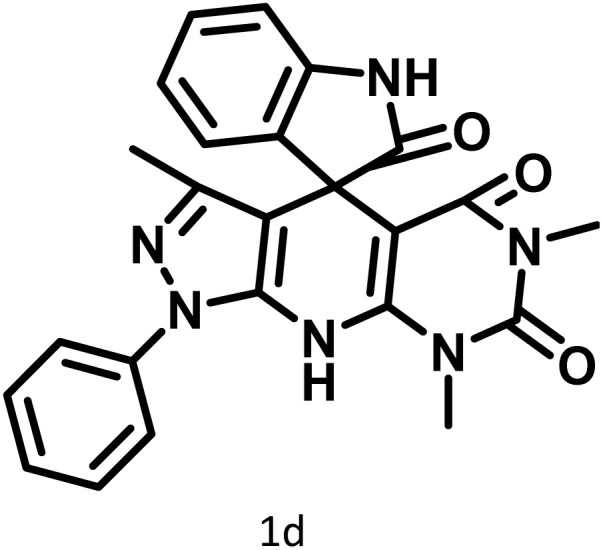	20	92	259–261	261–262 (ref. 55)
5	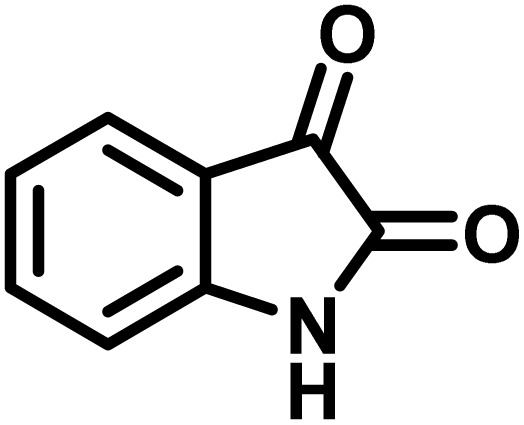	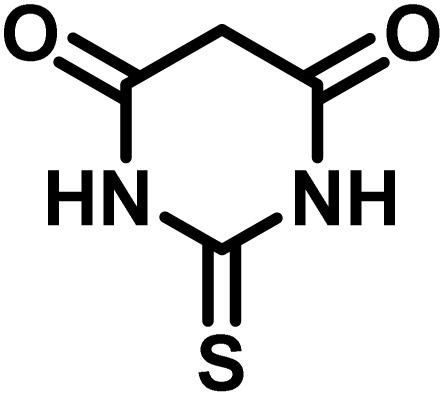	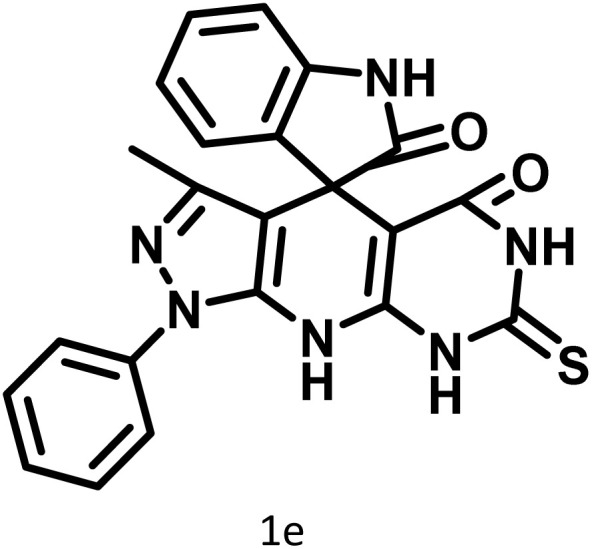	14	94	297–299	300 (ref. 21)
6	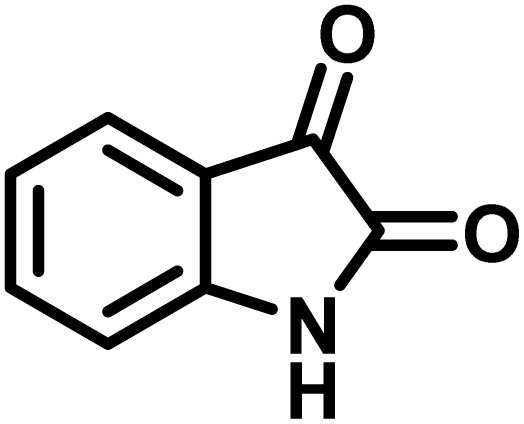	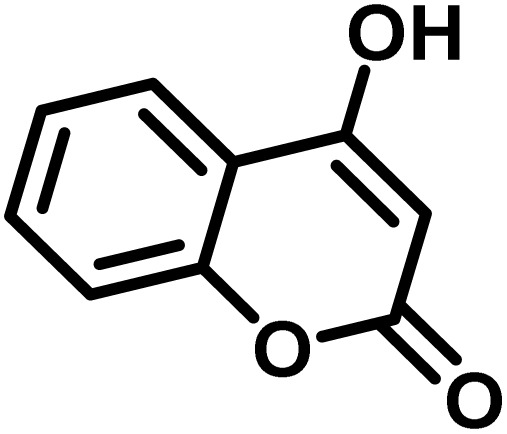	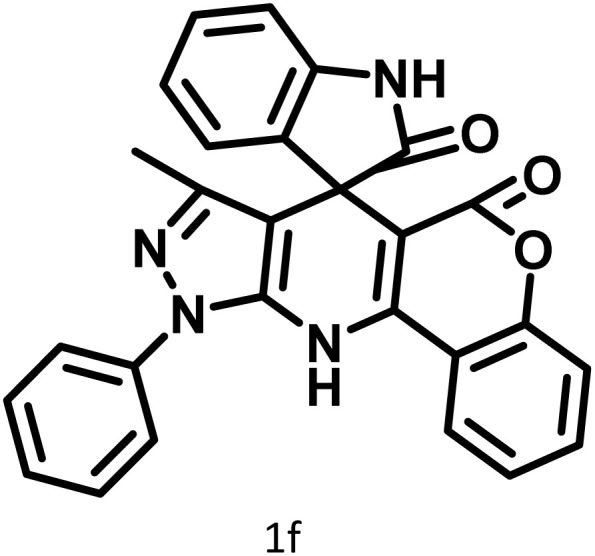	13	95	269–271	268–270 (ref. 20)
7	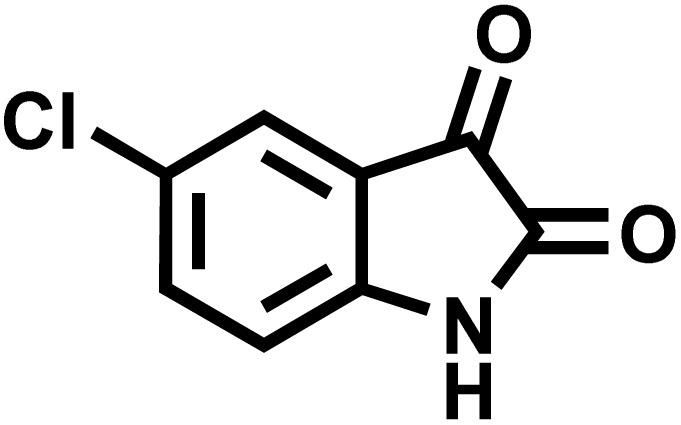	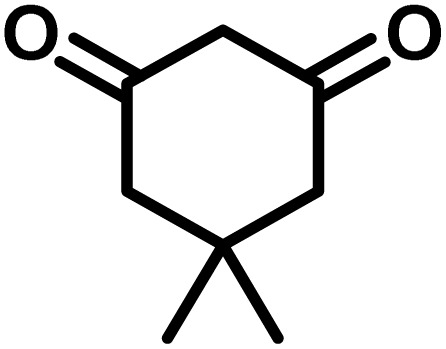	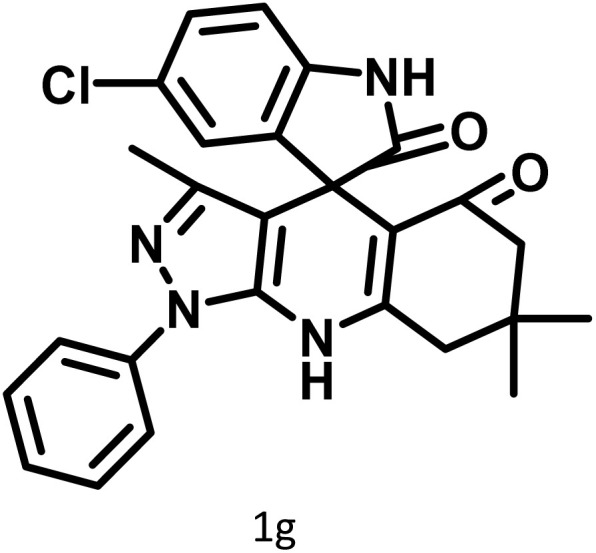	11	94	294–296	300 (ref. 21)
8	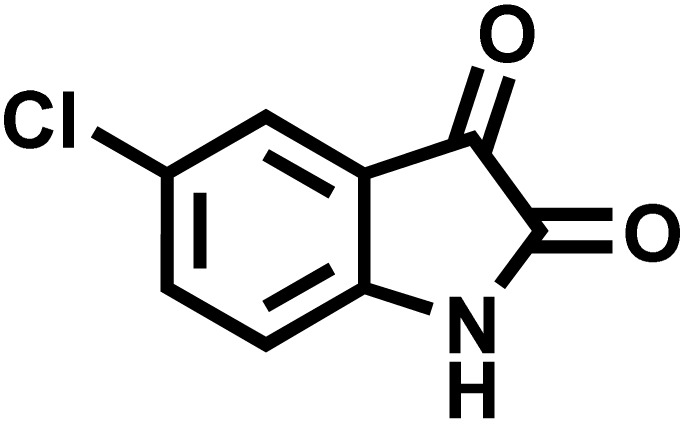	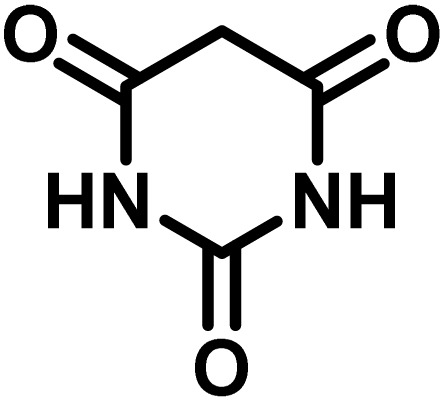	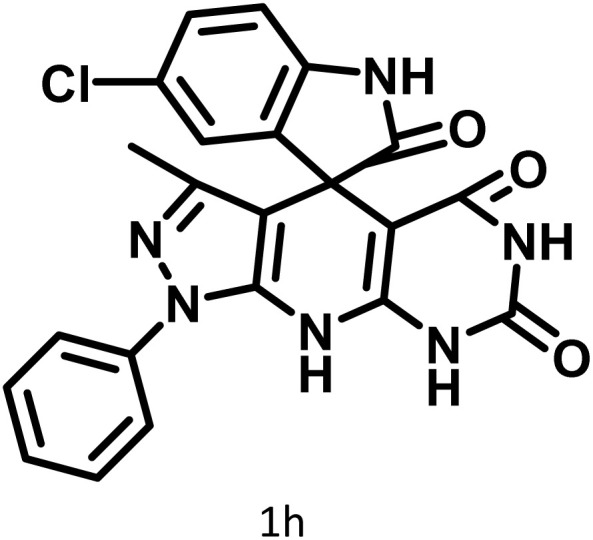	10	95	>300	300 (ref. 21)
9	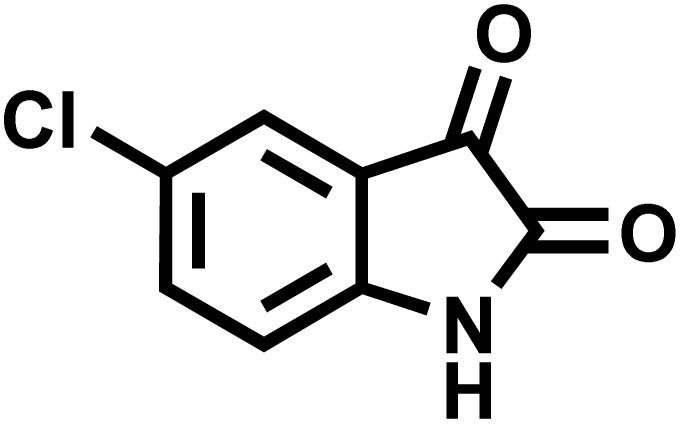	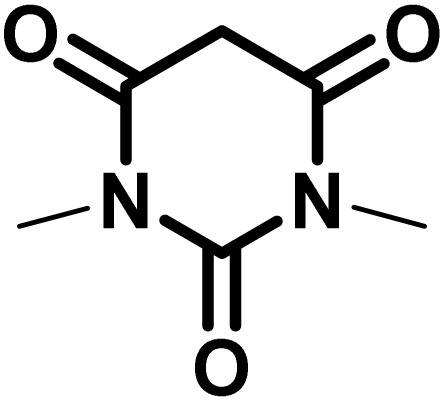	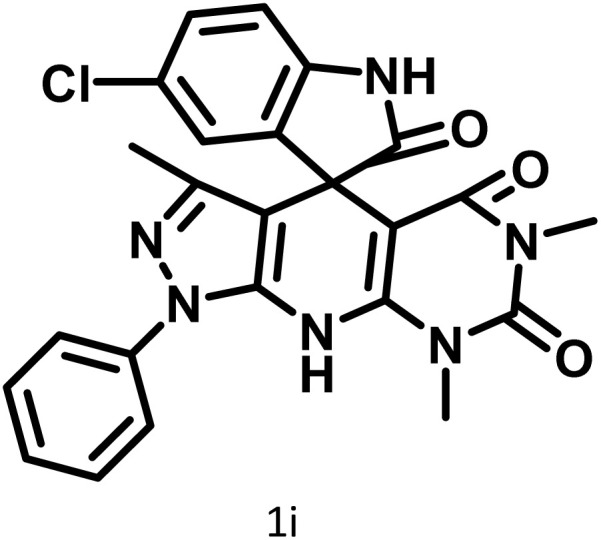	23	91	270–272	272–274 (ref. 55)
10	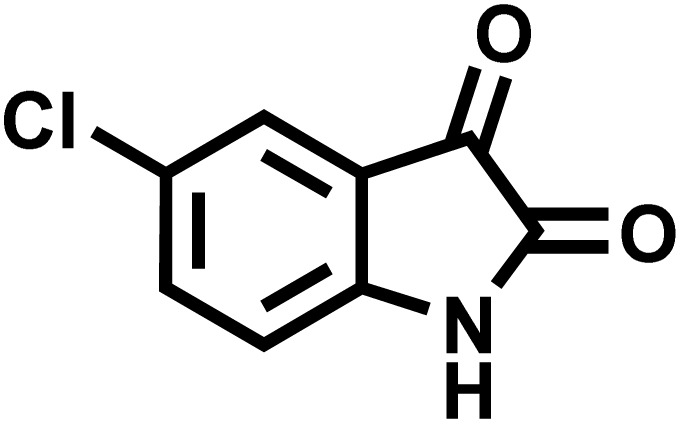	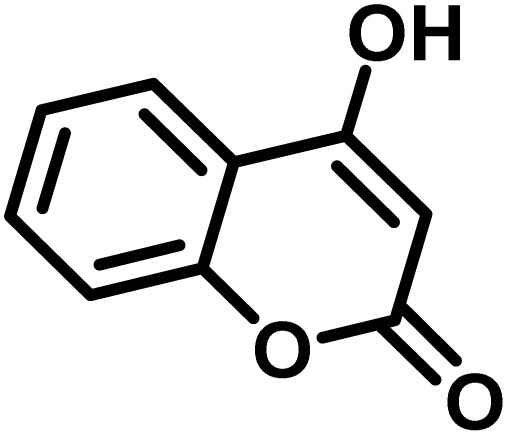	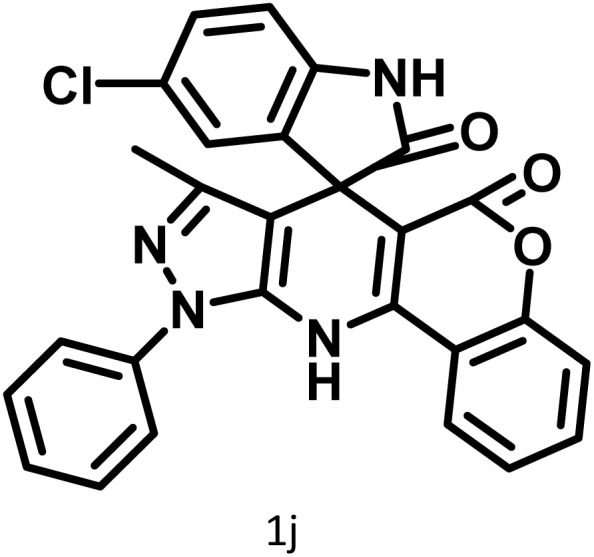	13	95	298–300	305–307 (ref. 20)
11	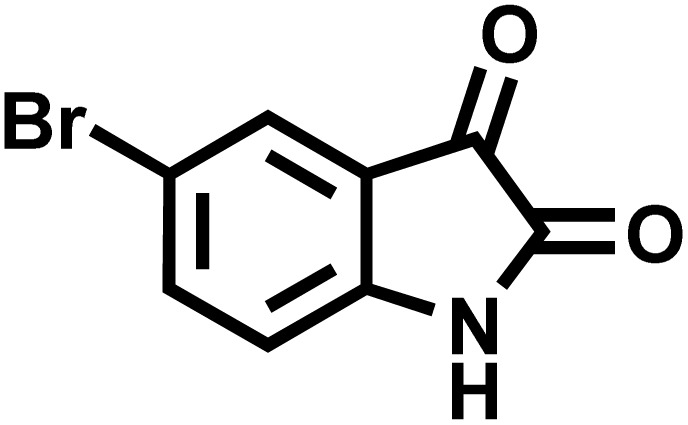	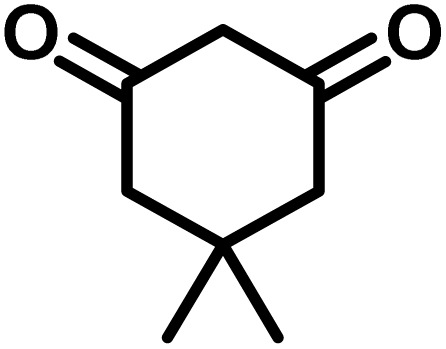	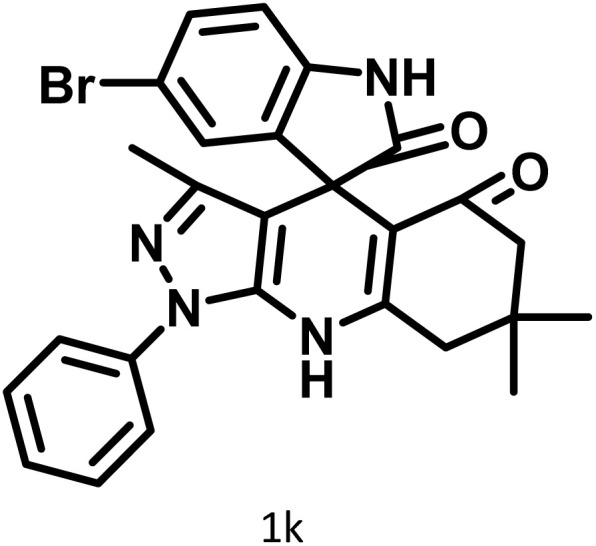	20	91	298–300	300 (ref. 21)
12	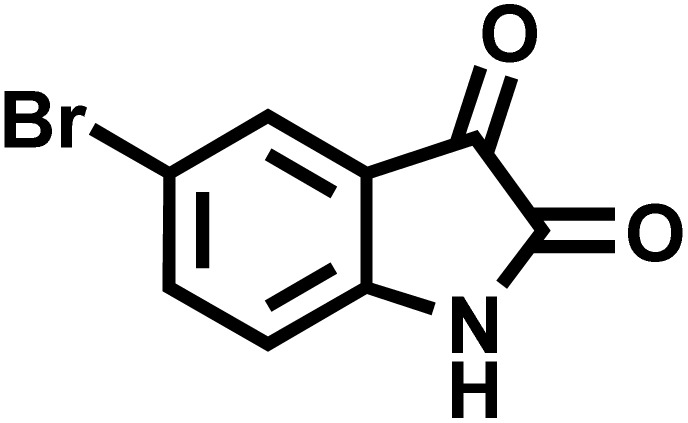	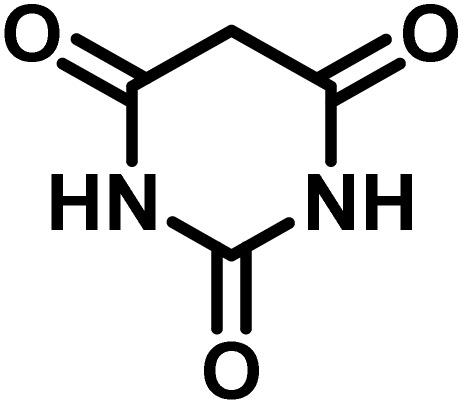	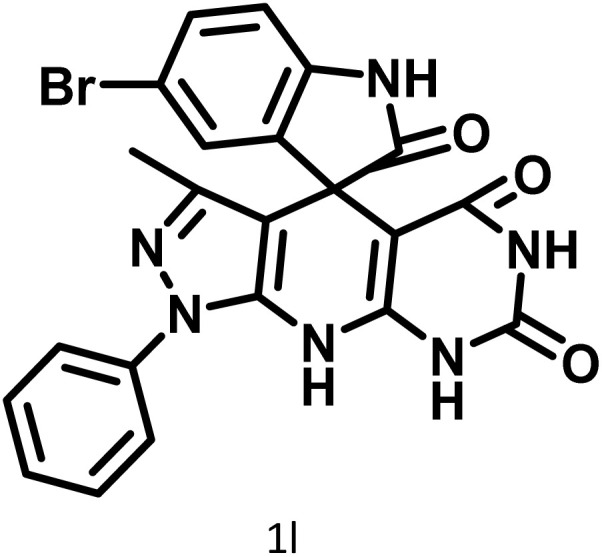	12	94	285–287	>280 (ref. 55)
13	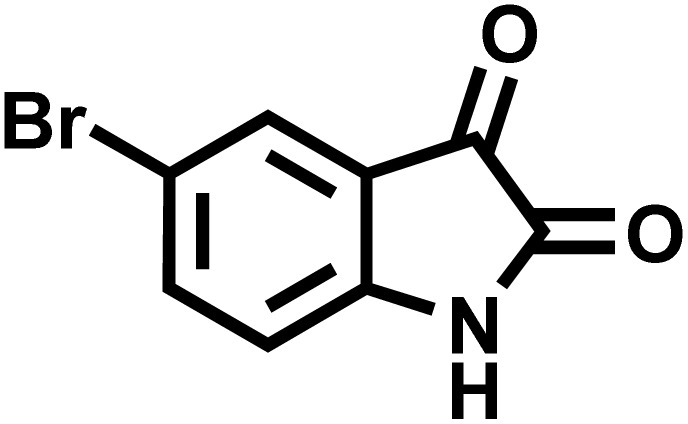	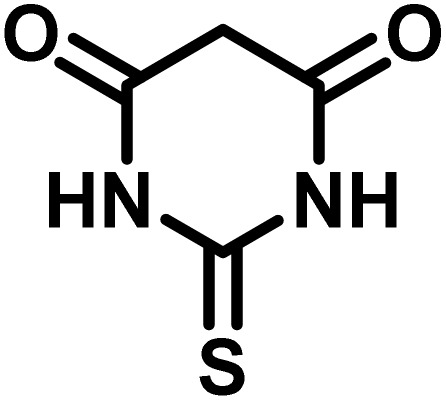	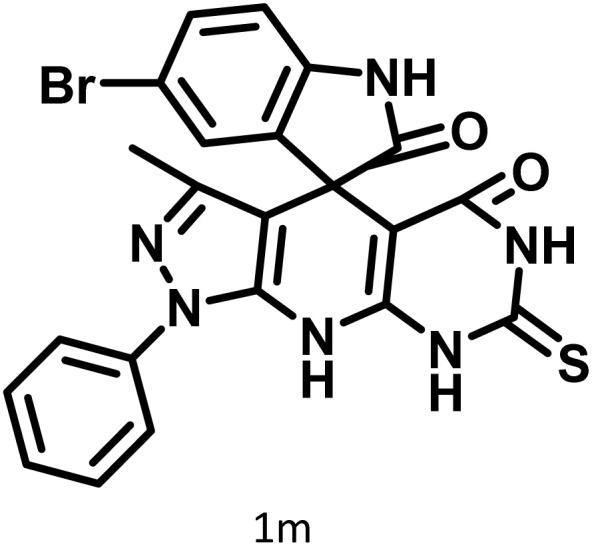	25	91	>300	300 (ref. 21)
14	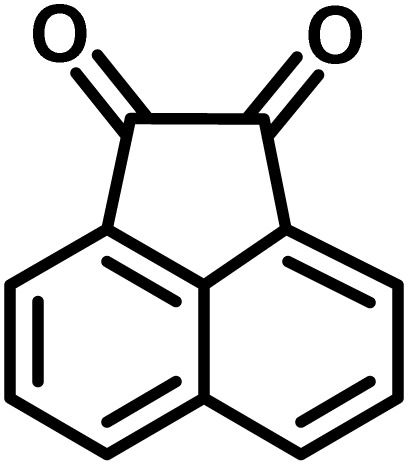	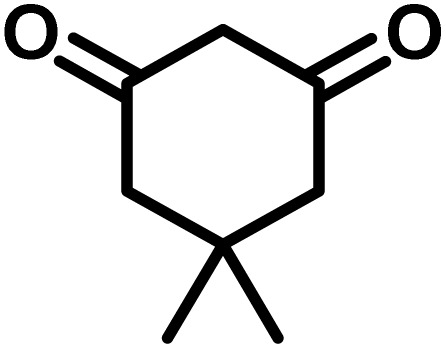	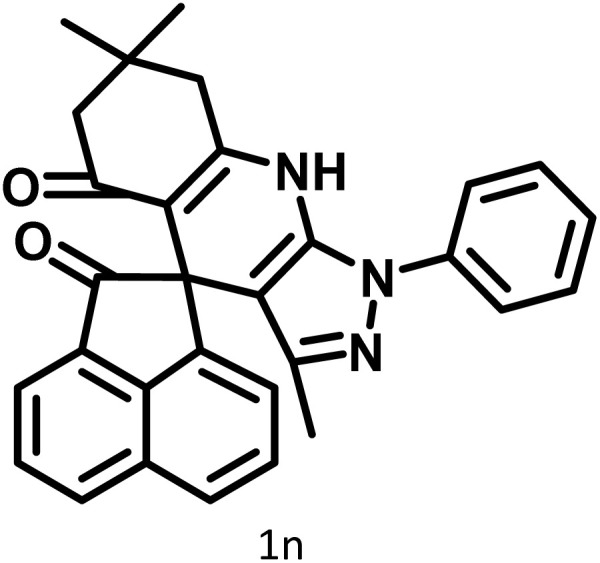	12	94	294–296	300 (ref. 21)
15	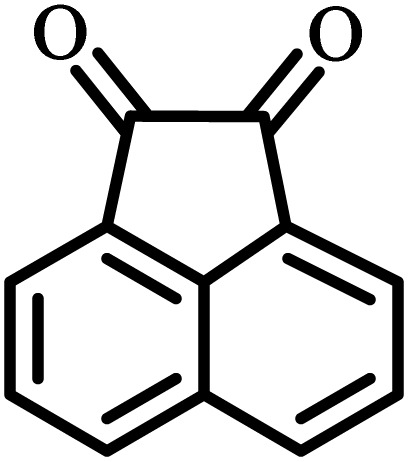	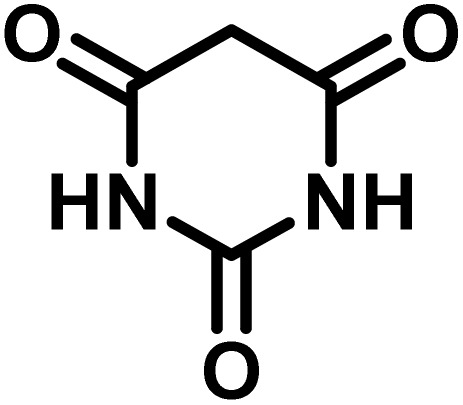	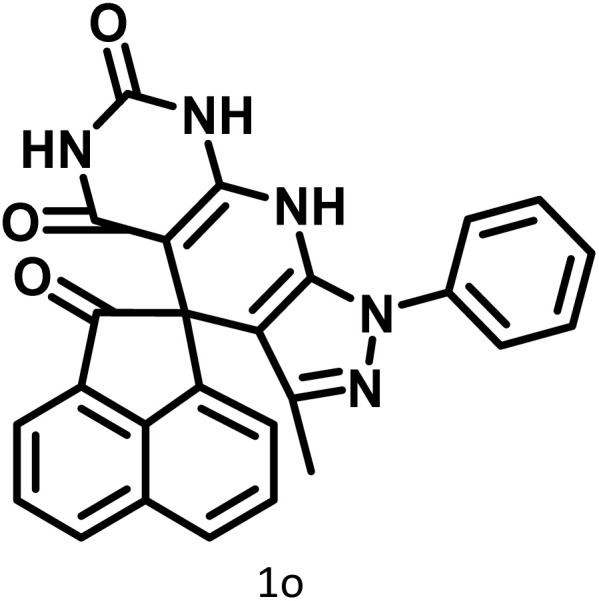	21	94	>300	300 (ref. 21)

a Reaction conditions: isatin (1 mmol), malononitrile (1.1 mmol), C–H activated acid derivatives (1 mmol), CS-(M_3_SP)_2_-NH_2_·HSO_4_ (20 mg), H_2_O (3 mL), 80 °C.

b Isolated yields.

**Scheme 3:**
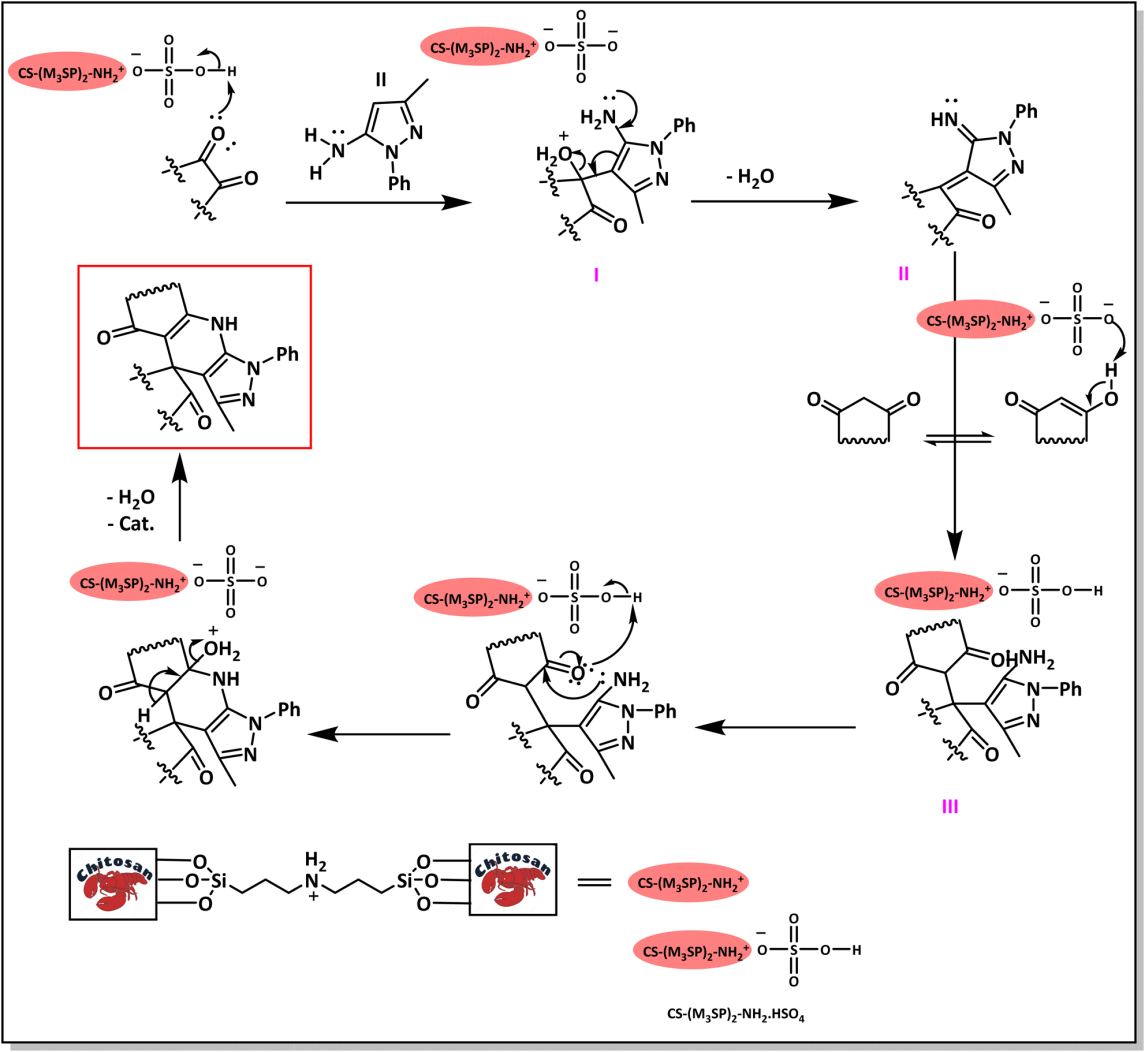
Plausible mechanism for the synthesis of (±)-spiro-indoline-3,4′-pyrazolo[3,4-*b*]-pyridines using CS-(M_3_SP)_2_-NH_2_·HSO_4_.

A plausible mechanism for the synthesis of (±)-spiro-indoline-3,4′-pyrazolo[3,4-*b*]-pyridines in the presence of CS-(M_3_SP)_2_-NH_2_·HSO_4_ is shown in Scheme 3. According to this mechanism, for the synthesis of spiro-oxindoles, at first, by nucleophilic addition of 5-amino-3-methyl-1-phenylpyrazole to the 1,2-dicarbonyl substrate, the intermediate (**I**) was formed. In continue loss of a molecule of water led to the formation of the intermediate (**II**). Then, the reaction of the acidic hydrogen-containing compound and the intermediate **II***via* Michael addition produced the intermediate (**III**). Next, nucleophilic attack of the NH_2_ group on the activated carbonyl group, followed by the removal of a molecule of water, provided the desired products (**1a–1o**) (Scheme 3).^54^ In this process, the –SO_3_H groups present in the catalyst, activate the carbonyl groups through hydrogen bonding and facilitate the formation of intermediates without participating in the reaction directly.

**Scheme 4:**
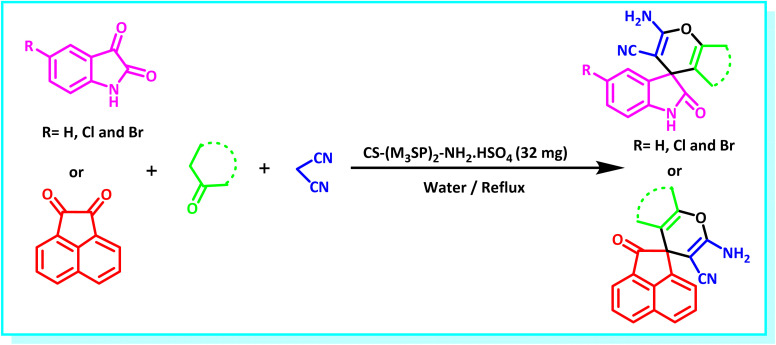
Synthesis of (±)-spiro-indoline-pyrano[2,3-*c*]-quinolines in the presence of CS-(M_3_SP)_2_-NH_2_·HSO_4_.

After the efficient effect of CS-(M_3_SP)_2_-NH_2_·HSO_4_ in the acceleration of the synthesis of (±)-spiro-indoline-3,4′-pyrazolo[3,4-*b*]-pyridines, the synthesis of (±)-spiro-indoline-pyrano[2,3-*c*]-quinolines using this promotor was also investigated. In order to determine the best conditions, the reaction of isatin, malononitrile and dimedone in the presence of CS-(M_3_SP)_2_-NH_2_·HSO_4_ was studied and the effect of various amounts of the catalyst in different solvents at a variety of temperatures was investigated on it. As Table 4 shows, the best results were obtained in the presence of 32 mg of CS-(M_3_SP)_2_-NH_2_·HSO_4_ in refluxing water (Table 4, entry 2) (Scheme 4).

**Table 4:** Optimization of the reaction conditions in the synthesis of (±)-2-amino-7,7-dimethyl-2′,5-dioxo-5,6,7,8-tetrahydrospiro[chromene-4,3′-indoline]-3-carbonitrile^a^

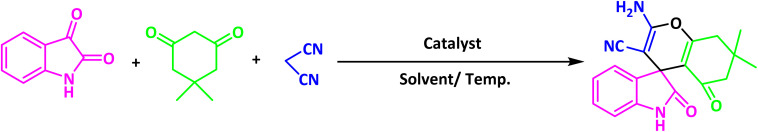					
Entry	Catalyst (mg)	Solvent	Temp. (^°^C)	Time (min.)	Yield^b^ (%)
1	20	Water	Reflux	30	97
2	32	Water	Reflux	6	98
3	40	Water	Reflux	6	97
4	20	Ethanol	Reflux	45	66
5	32	Ethanol	Reflux	18	96
6	32	Water/ethanol (1 : 1)	Reflux	38	95
7	32	Acetonitrile	Reflux	45	54
8	32	Solvent-free	100	45	63

a Reaction conditions: isatin (1 mmol), malononitrile (1.1 mmol), dimedone (1 mmol), solvent (3 mL), catalyst.

b Yield values were determined by HPLC analysis using the product as an external standard.

In continue the effect of the optimized conditions was studied on the reaction of a variety of acidic hydrogen containing compounds and malononitrile with isatin derivatives and/or acenaphthoquinone. The results are tabulated in Table 5. These results considerably demonstrate the ability of the prepared catalyst in the acceleration of the synthesis of various types of the requested products during short times with good-to-excellent yields (>∼90%) (**2a–2r**).

**Table 5:** Synthesis of (±)-spiro-indoline-pyrano[2,3-*c*]-quinoline derivatives using CS-(M_3_SP)_2_-NH_2_·HSO_4_ as an ionic heterogeneous catalyst^a^

Entry	Carbonyl compound	Acidic hydrogen-containing compound	Product	Time (min.)	Yield^b^ (%)	M. P. (^°^C)	
						Found	Reported ^Ref.^
1	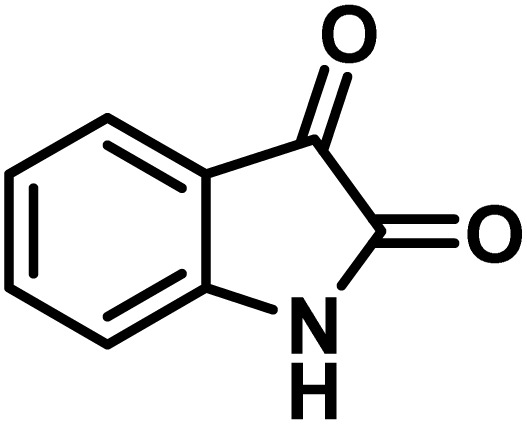	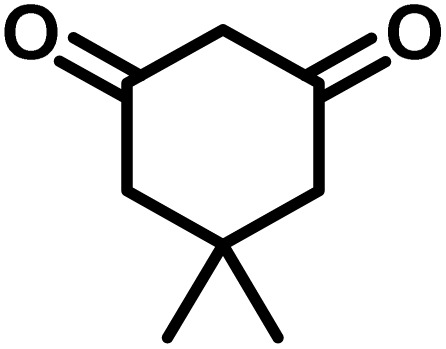	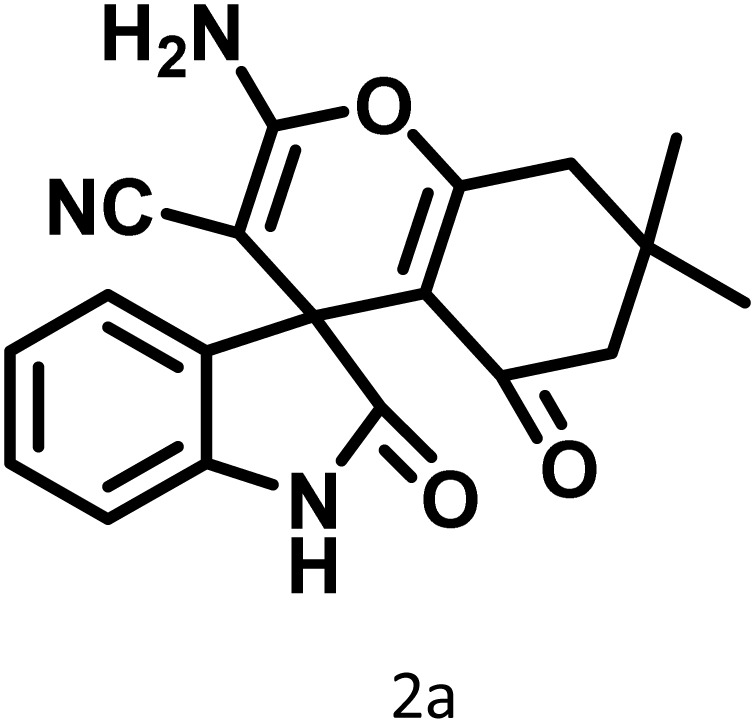	6	97	296–298	296–298 (ref. 21)
2	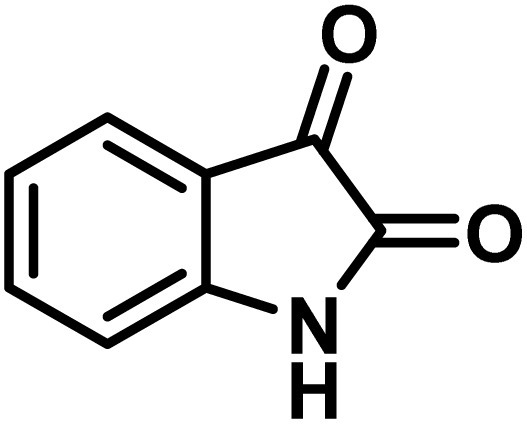	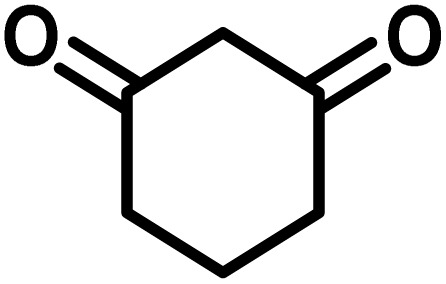	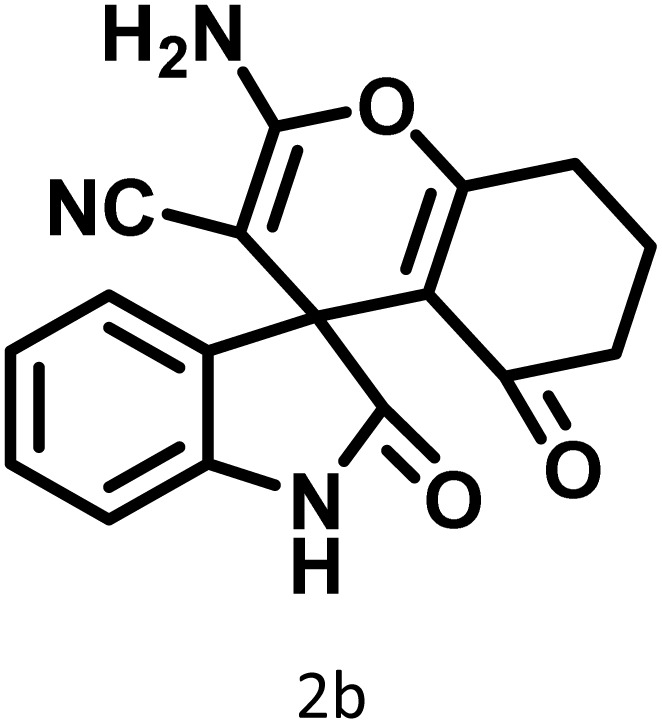	2	98	281–283	278–280 (ref. 56)
3	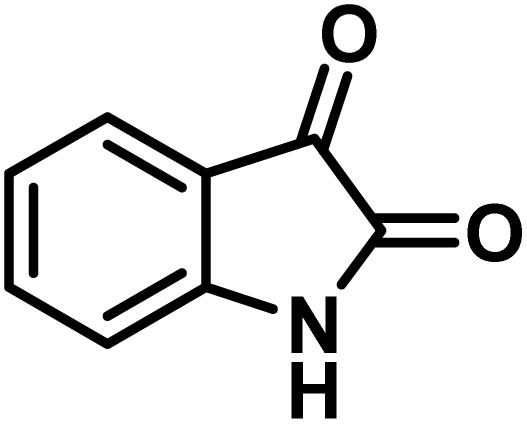	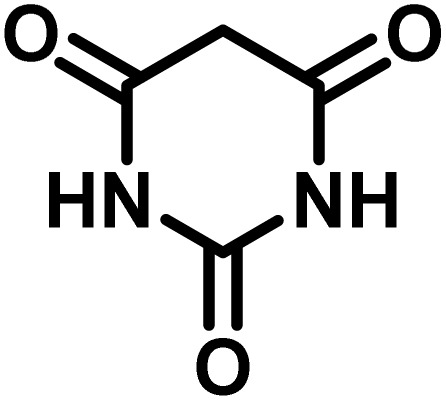	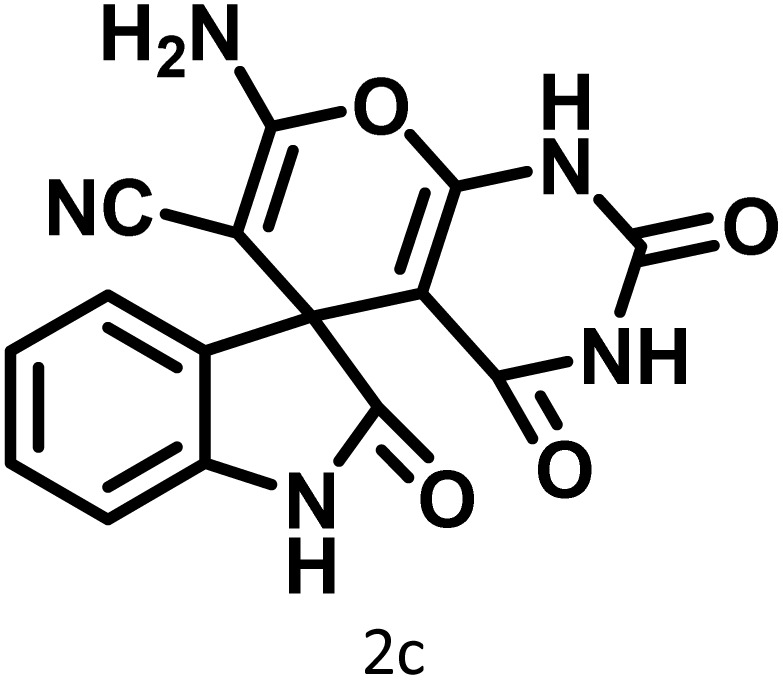	10	97	266–269	265–267 (ref. 57)
4	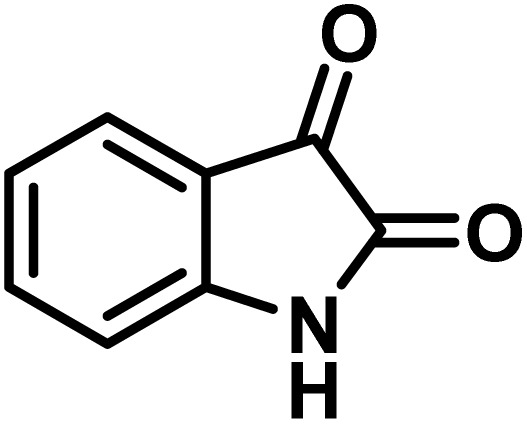	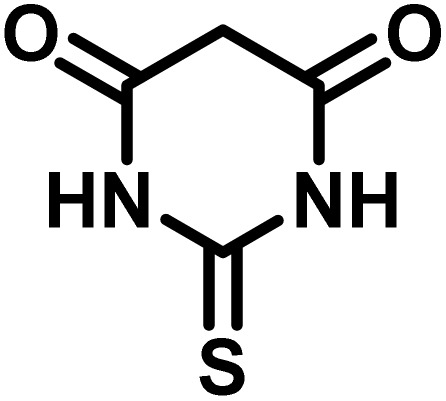	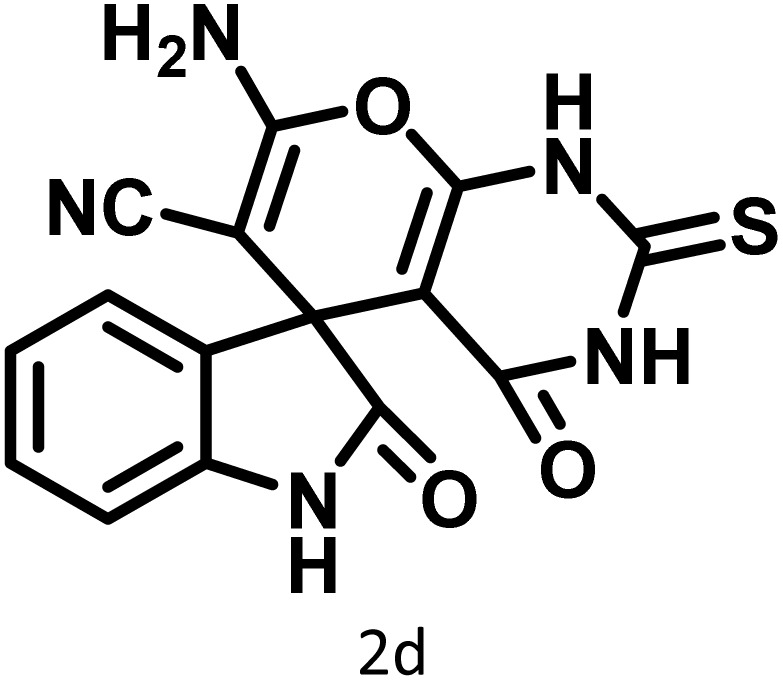	4	98	225–227	232–235 (ref. 58)
5	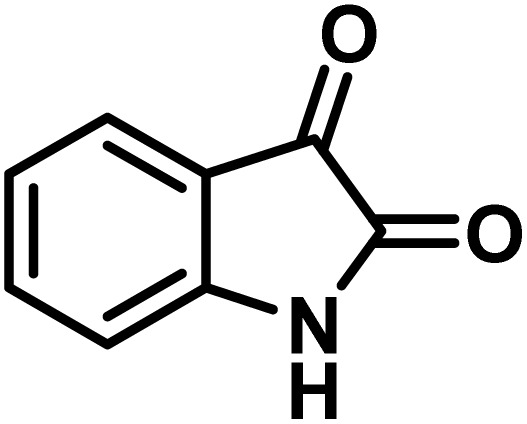	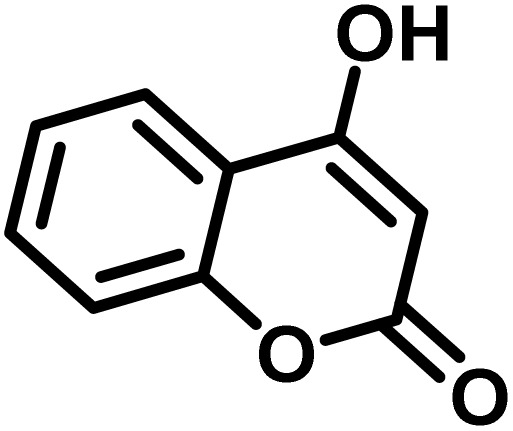	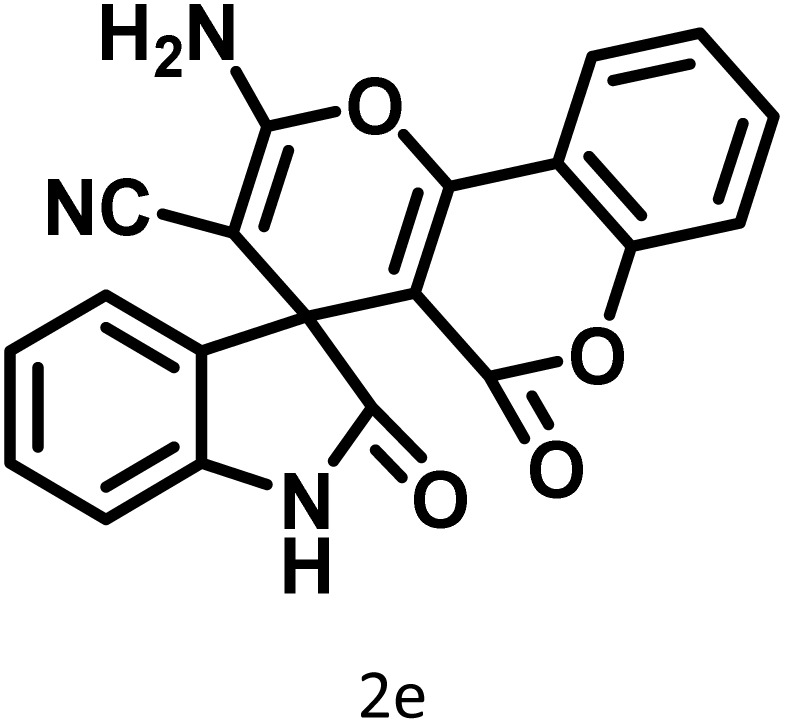	8	96	285–287	290–292 (ref. 57)
6	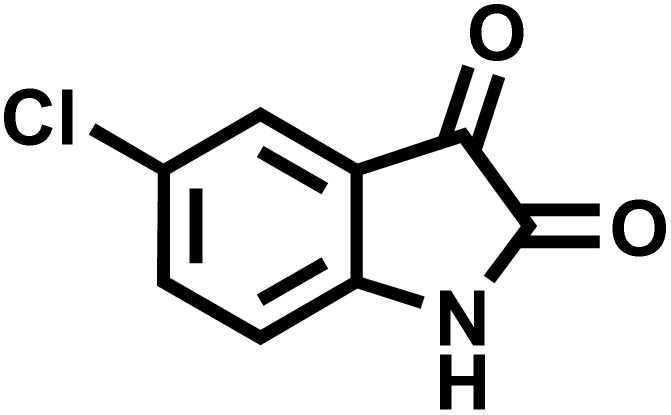	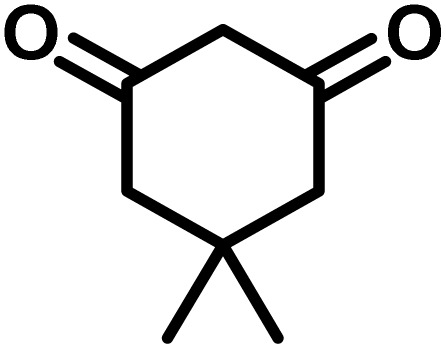	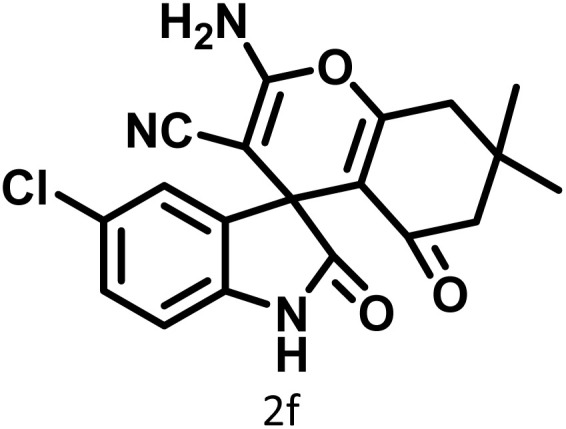	14	96	303–307	300 (ref. 59)
7	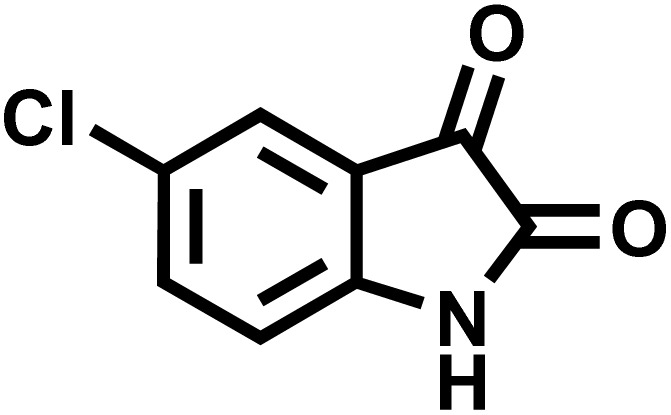	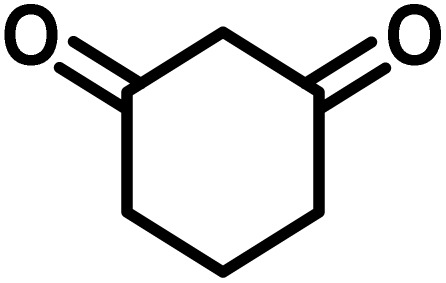	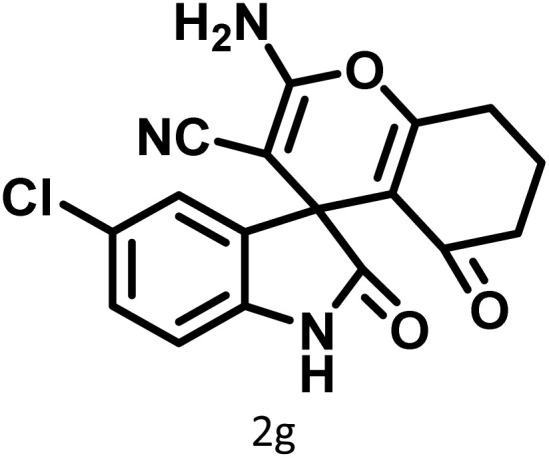	10	97	285–287	285–287 (ref. 57)
8	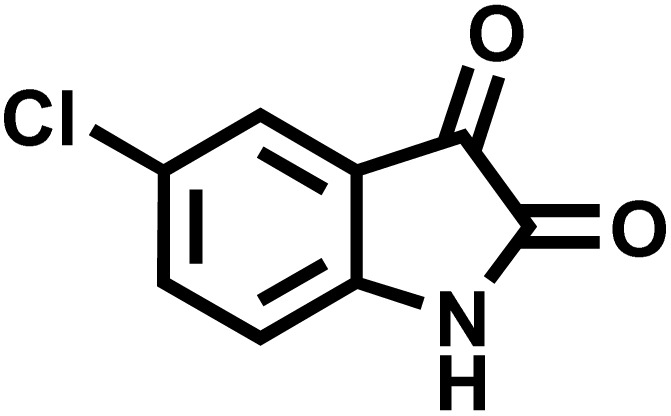	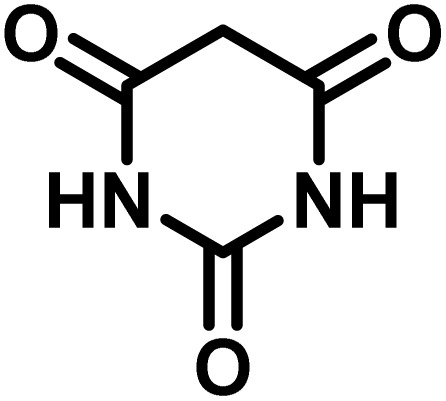	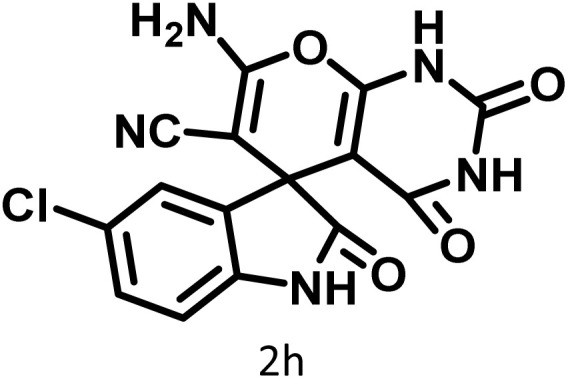	12	95	247–250	248–250 (ref. 60)
9	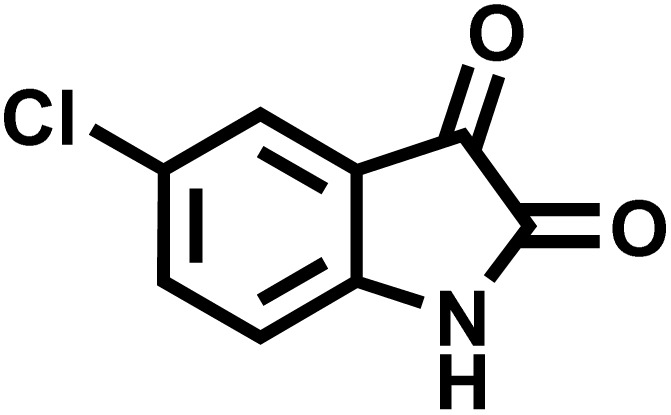	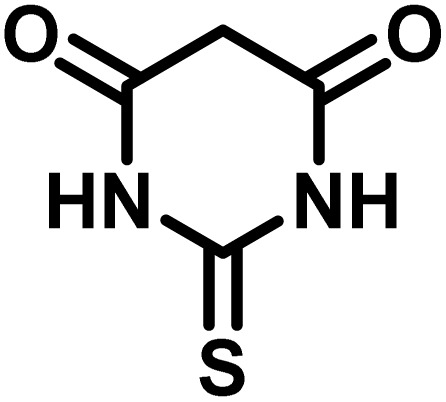	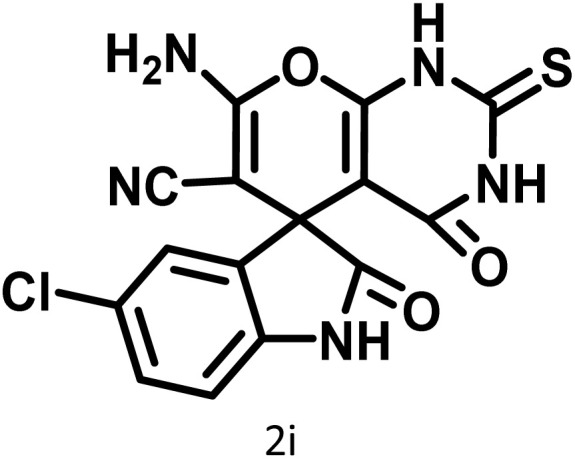	14	94	241–244	228–230 (ref. 61)
10	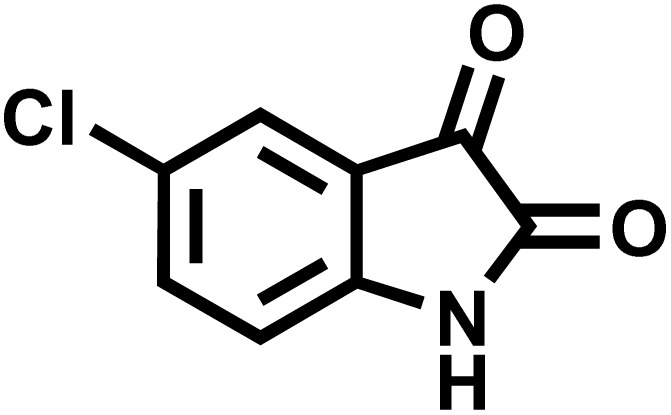	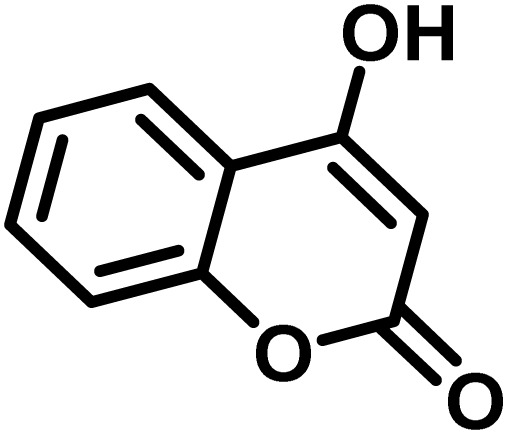	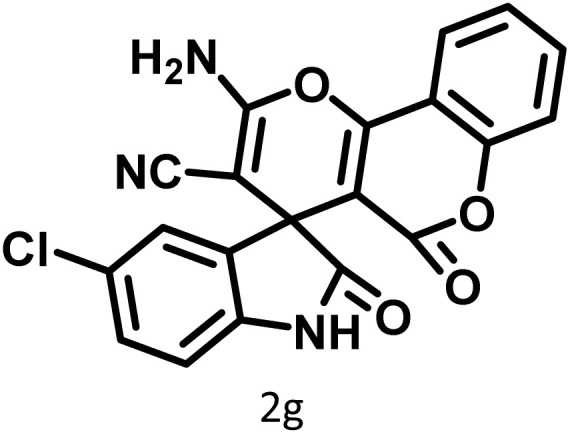	16	95	312–314	318–320 (ref. 60)
11	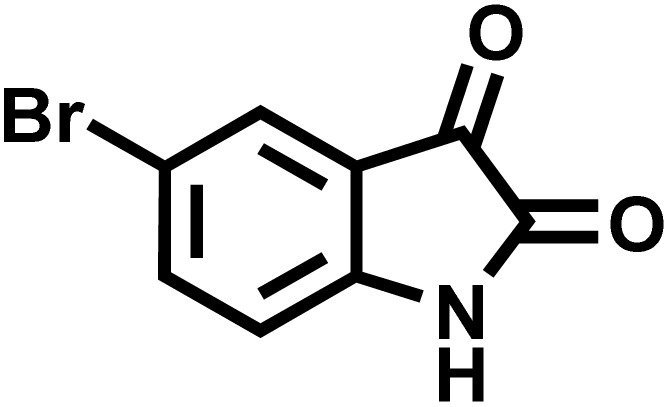	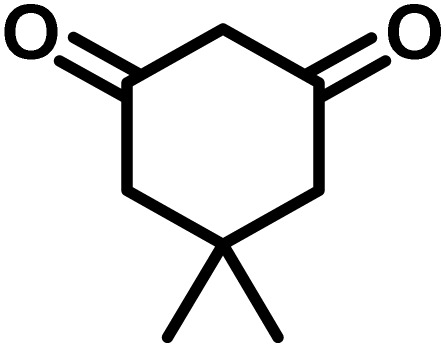	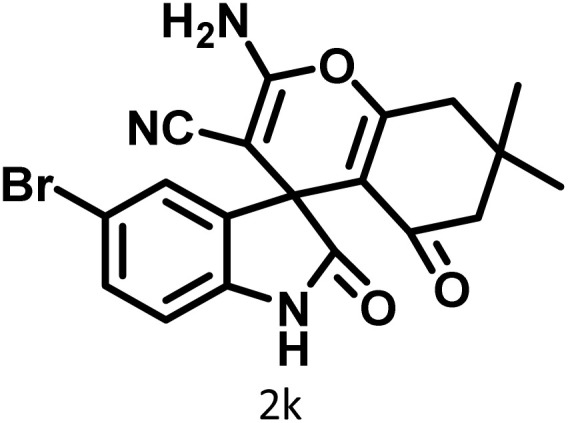	11	97	296–298	295–297 (ref. 62)
12	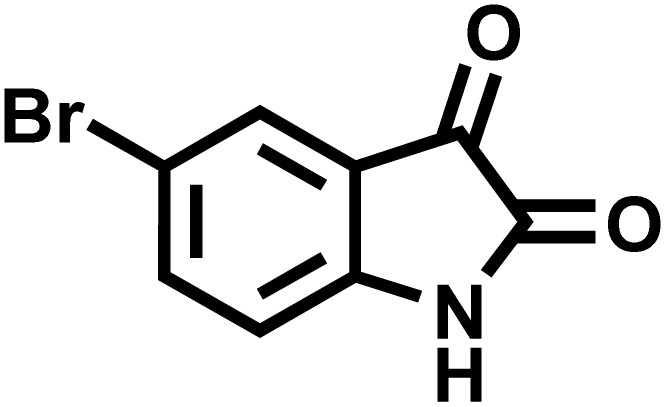	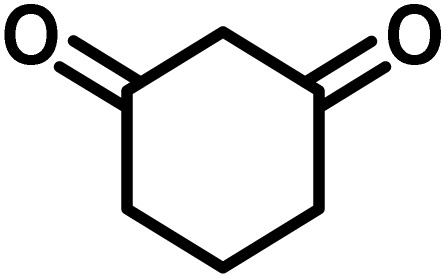	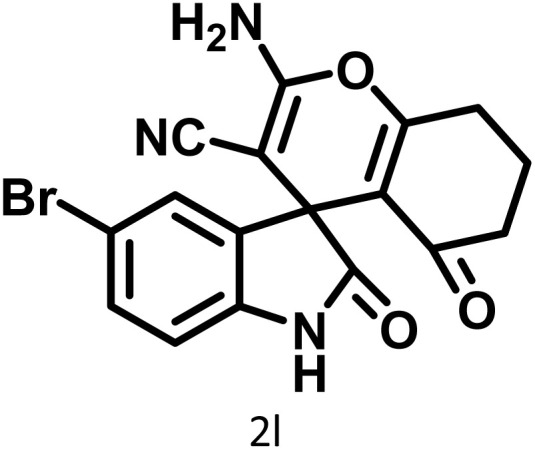	8	98	277–279	276–278 (ref. 63)
13	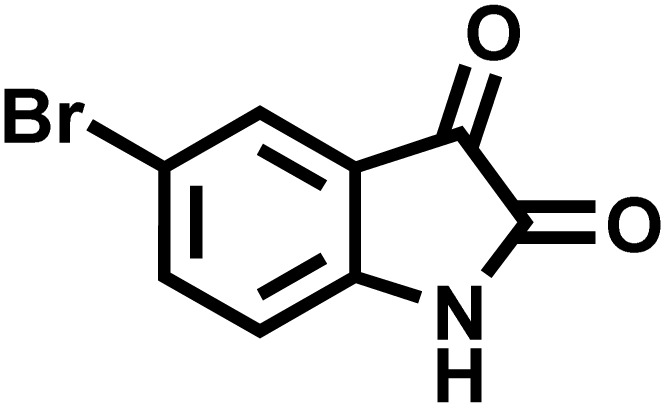	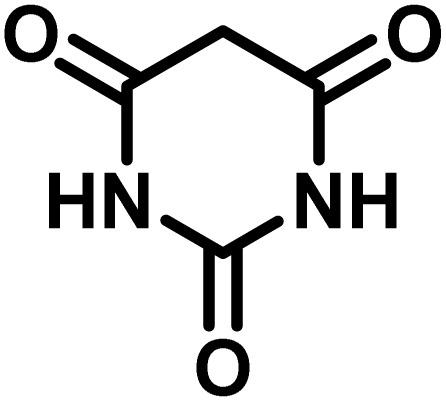	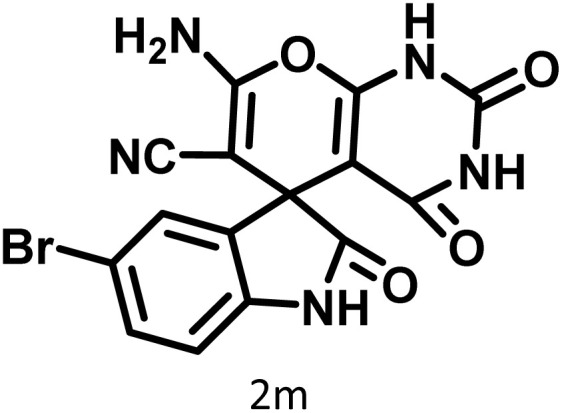	9	96	251–253	254–256 (ref. 60)
14	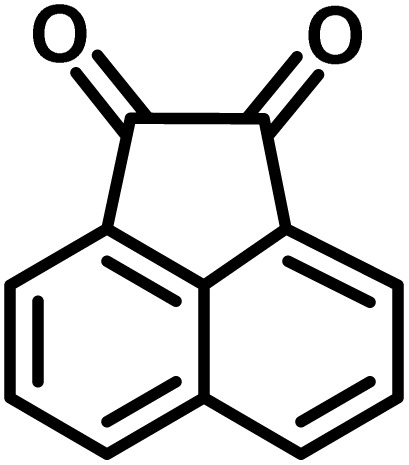	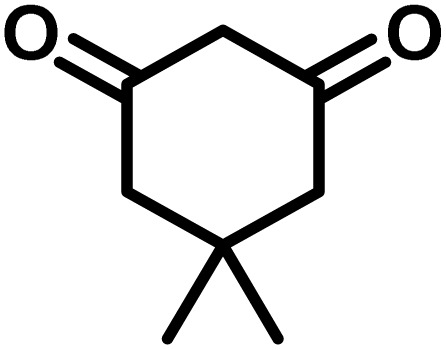	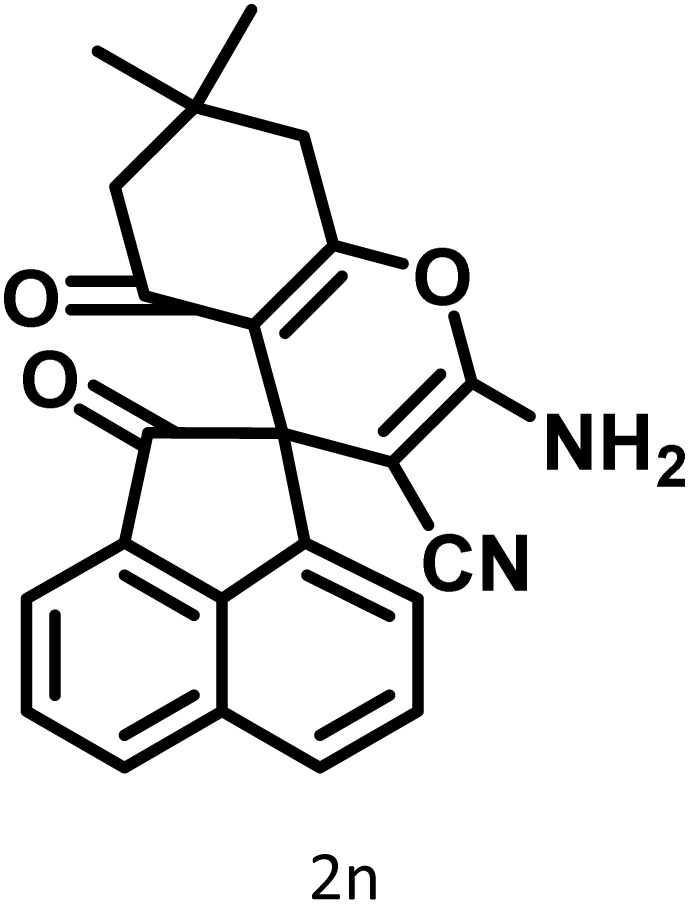	11	95	289–291	288–290 (ref. 64)
15	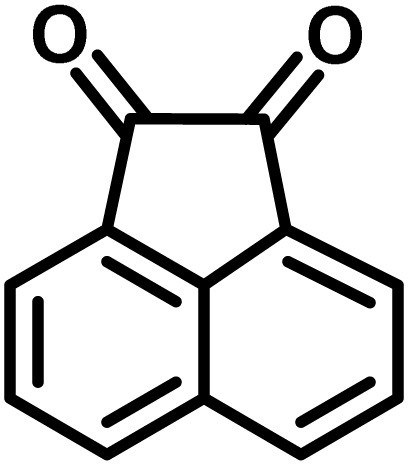	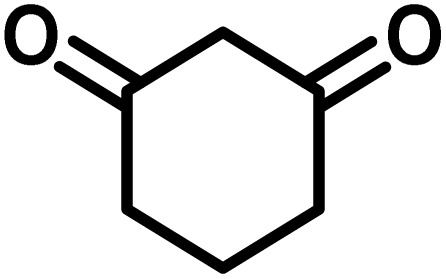	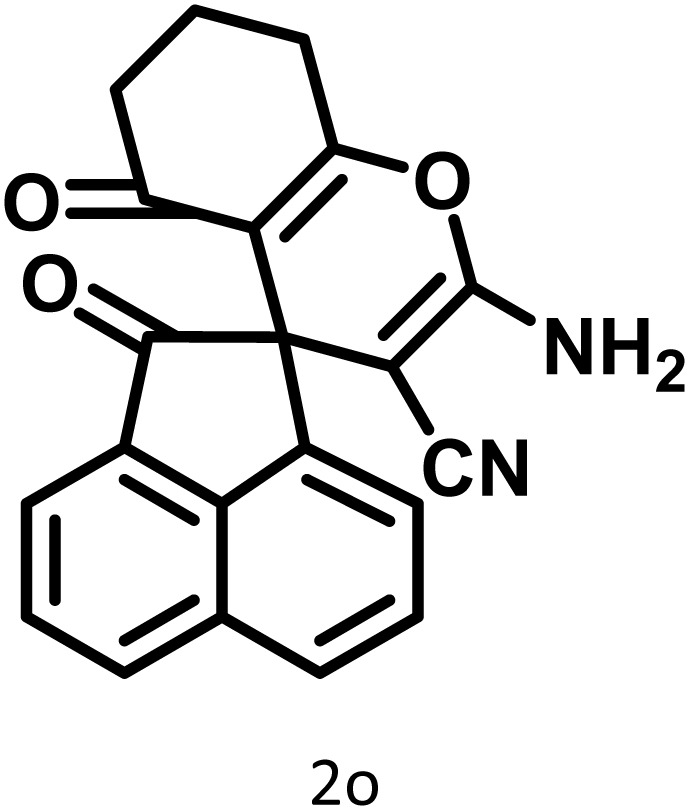	6	97	244–246	244–246 (ref. 15)
16	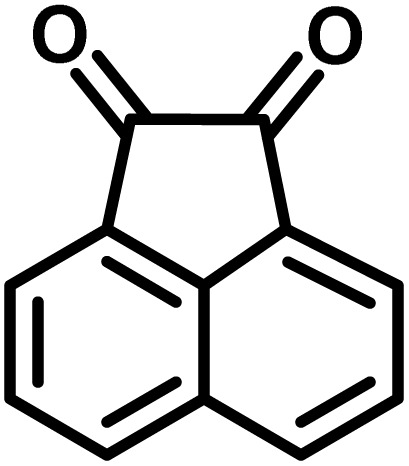	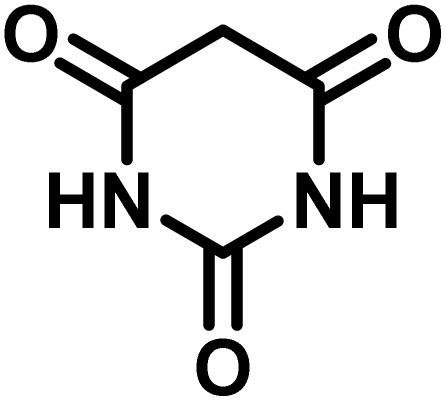	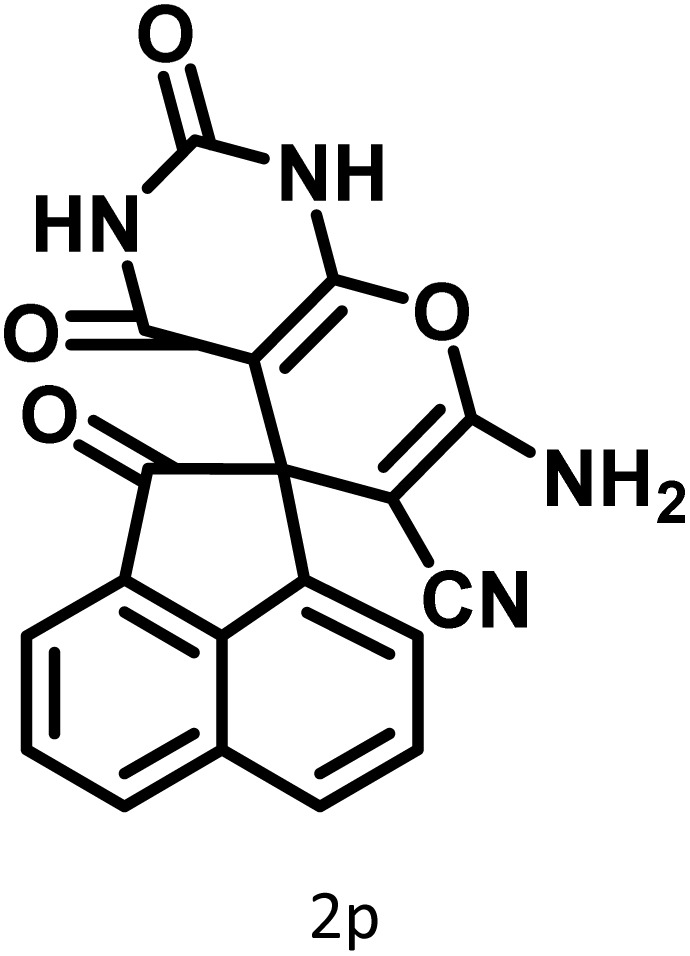	22	95	298–300	300 (ref. 65)
17	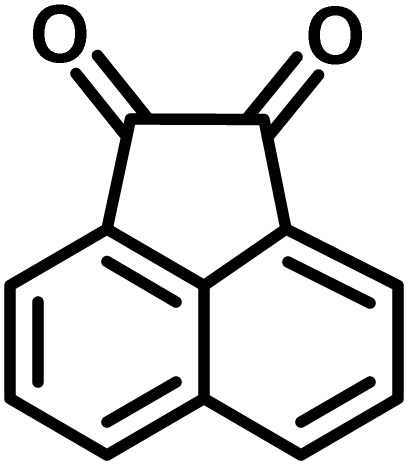	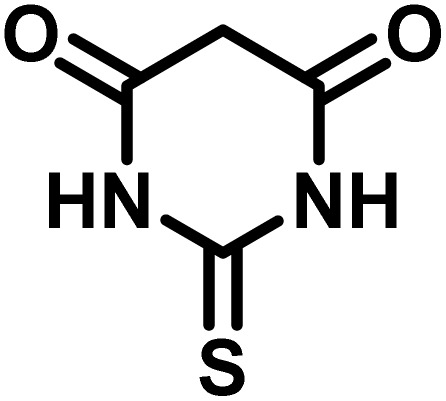	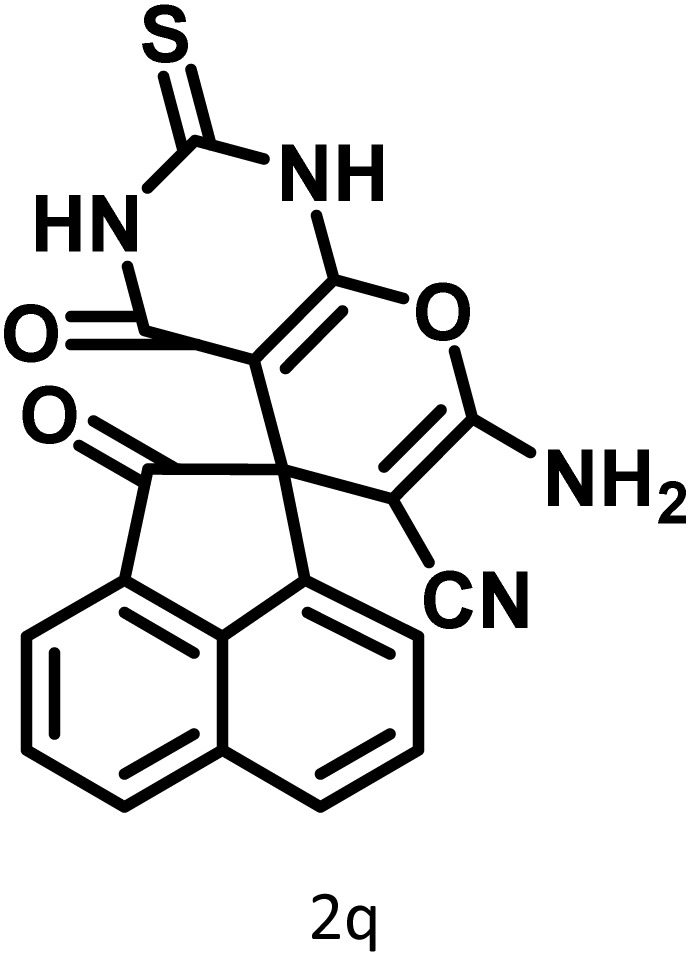	25	94	214–216	213–215 (ref. 66)
18	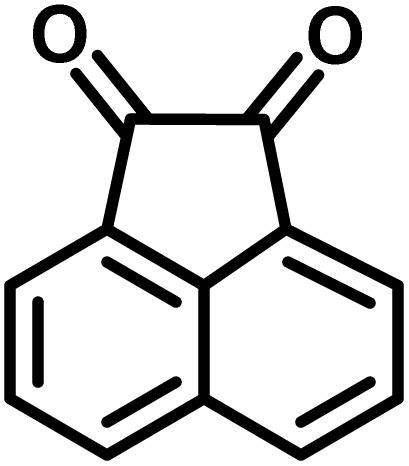	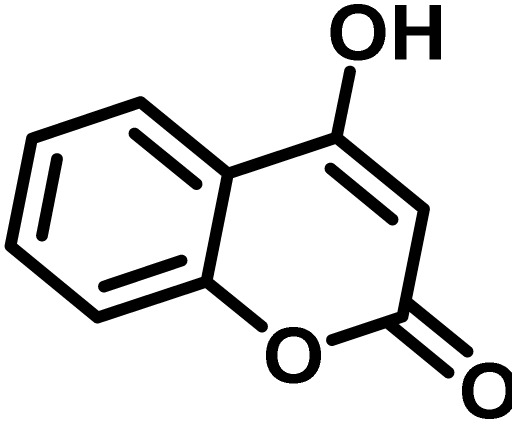	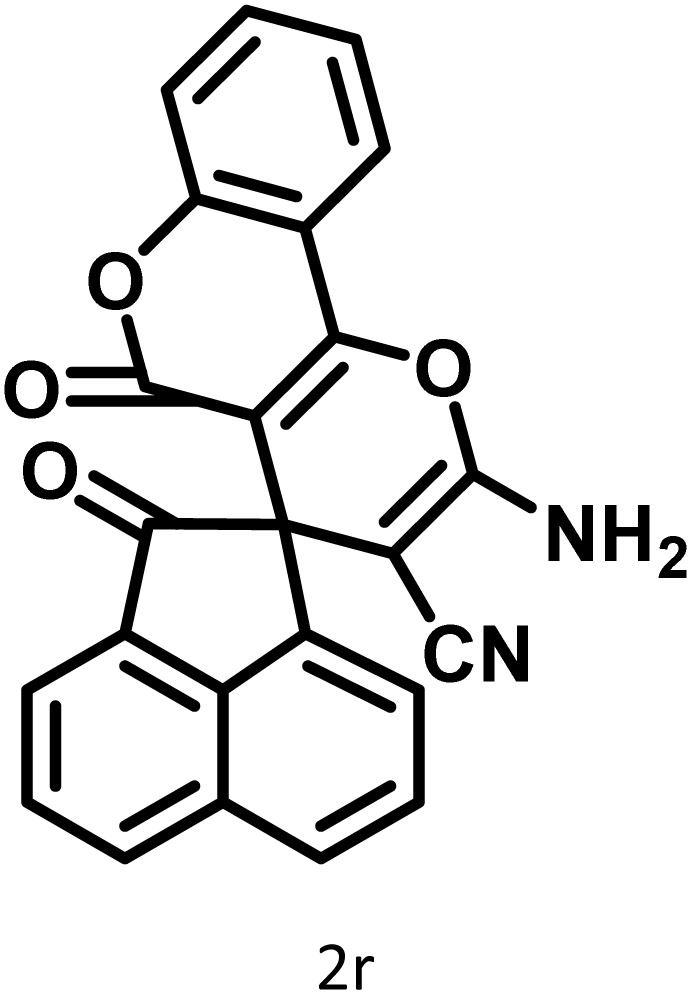	30	93	298–300	300 (ref. 67)

a Reaction conditions: isatin (1 mmol), malononitrile (1.1 mmol), C–H activated acid derivatives (1 mmol), CS-(M_3_SP)_2_-NH_2_·HSO_4_ (32 mg), H_2_O (3 mL), reflux.

b Isolated yields.

**Scheme 5:**
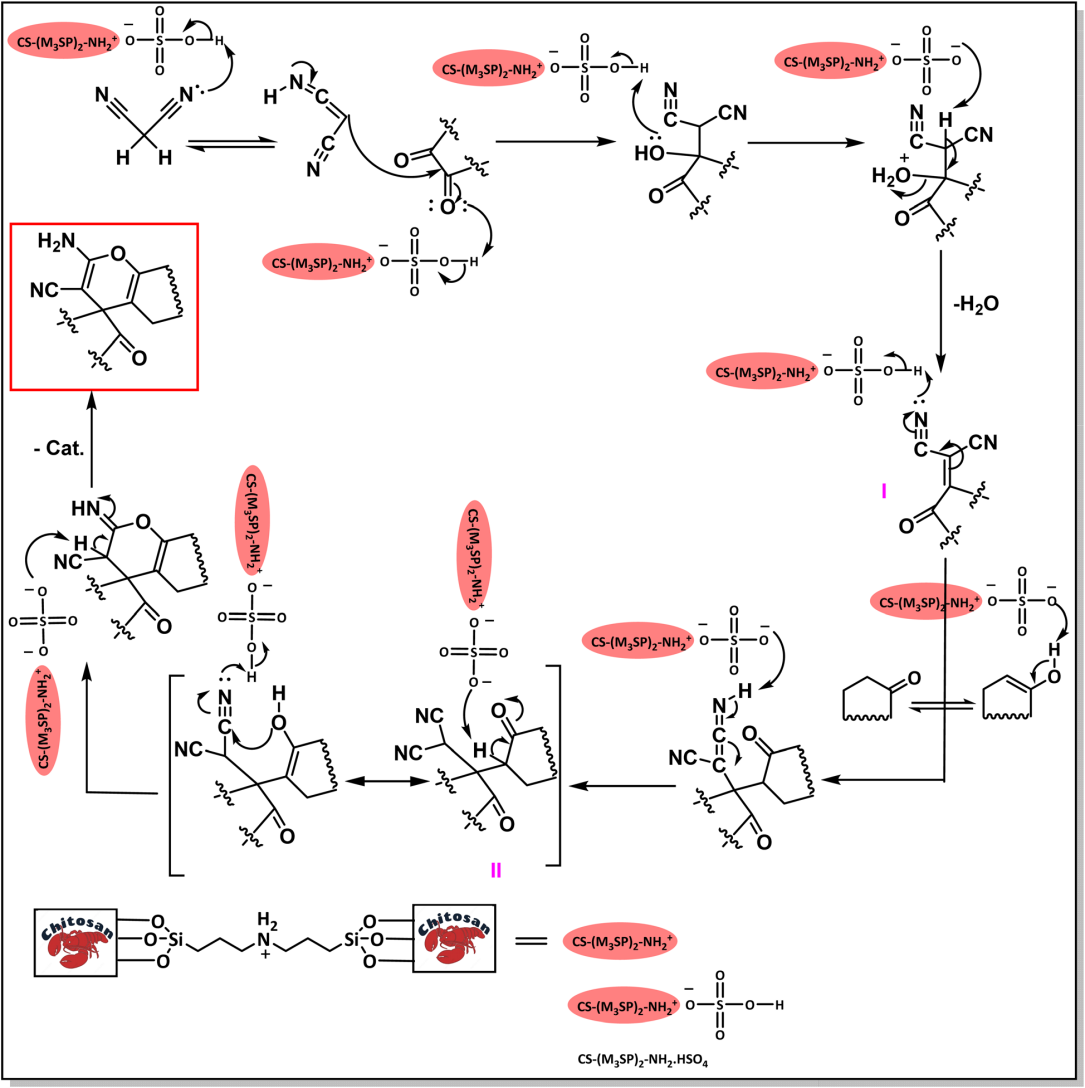
The proposed mechanism for the synthesis of (±)-spiro-indoline-pyrano[2,3-*c*]-quinoline derivatives using CS-(M_3_SP)_2_-NH_2_·HSO_4_ as a catalyst.

The probable pathway of the synthesis of (±)-spiro-indoline-pyrano[2,3-*c*]-quinoline derivatives in the presence of CS-(M_3_SP)_2_-NH_2_·HSO_4_ is suggested in Scheme 5. According to this mechanism, the catalyst acted as a promotor for the tautomerization of malononitrile and acidic hydrogen containing compounds, as well as the carbonyl group of 1,2-dicarbonyl substrate. The Knoevenagel-type coupling between malononitrile and the carbonyl carbon of 1,2-dicarbonyl compound, resulted in the formation of the intermediate (**I**). Next, the reaction between the acidic hydrogen containing compound and the intermediate (**I**) *via* Michael addition led to the intermediate (**III**). Finally, the intermediate (**III**) underwent intramolecular cyclization and hydrogen transfer, which ultimately led to the requested target products.^28^

To evaluate the effectiveness of the investigated catalyst, its performance in the synthesis of (±)-spiro-indoline-3,4-pyrazolo[3,4-*b*]-pyridines and (±)-spiro-indoline-pyrano[2,3-*c*]-quinolines by CS-(M_3_SP)_2_-NH_2_·HSO_4_ was compared with some of the results obtained using other reported catalysts (Table 6). It is worth mentioning that CS-(M_3_SP)_2_-NH_2_·HSO_4_ showed higher efficiency than other catalysts in terms of the catalyst loading, reaction conditions, times, and yields of the products. According to these favorable results, this method was found to be promising for the effective synthesis of the aimed molecules. It should be mentioned that CS and CS-(M_3_SP)_2_-NH could also promote the compared reactions but required higher reaction times with lower yields (Table 6). This observation showed the necessity of the changes made on chitosan to reach to more efficient catalyst for the desired reactions.

**Table 6:** Performance of CS-(M_3_SP)_2_-NH_2_·HSO_4_ in the synthesis of (±)-3′,7′,7′-trimethyl-1′-phenyl-1′,7′,8′,9′-tetrahydrospiro[indoline-3,4′-pyrazolo[3,4-*b*]quinoline]-2,5′(6′*H*)-dione and (±)-2-amino-7,7-dimethyl-2′,5-dioxo-5,6,7,8-tetrahydrospiro[chromene-4,3′-indoline]-3-carbonitrile compared to some of the reported catalytic systems

Product	Catalyst (amount)	Solvent/Temp. (°C)	Time (min.)	Yield^a^ (%)	Ref.
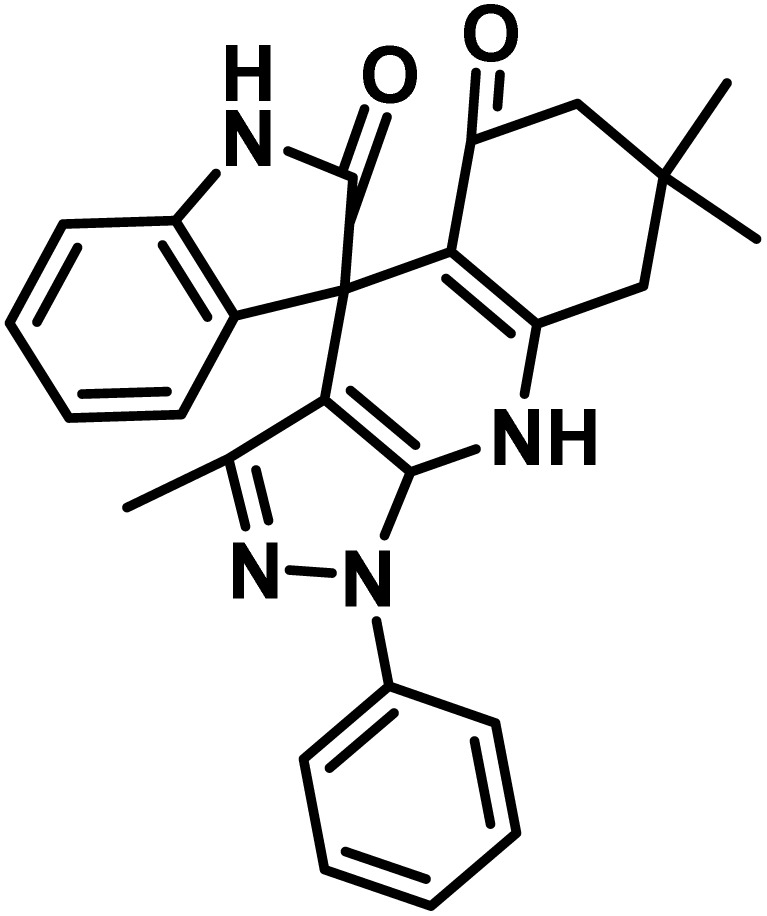	—	H_2_O/80 °C	60	44	This work
	CS (20 mg)	H_2_O/80 °C	60	55	This work
	CS-(M_3_SP)_2_-NH (20 mg)	H_2_O/80 °C	60	68	This work
	CS-(M_3_SP)_2_-NH_2_·HSO_4_ (20 mg)	H_2_O/80 °C	6	98	This work
	CSA (50 mol%)	H_2_O/reflux	3h	90	21
	*p*-TSA (100 mg)	H_2_O/EtOH [5 : 1 (v/v))]/80 °C	6h	75	41
	CAN (20 mol%)	H_2_O/80 °C	8h	88	54
	Papain (80 mg)	EtOH/50 °C	72h	32	40
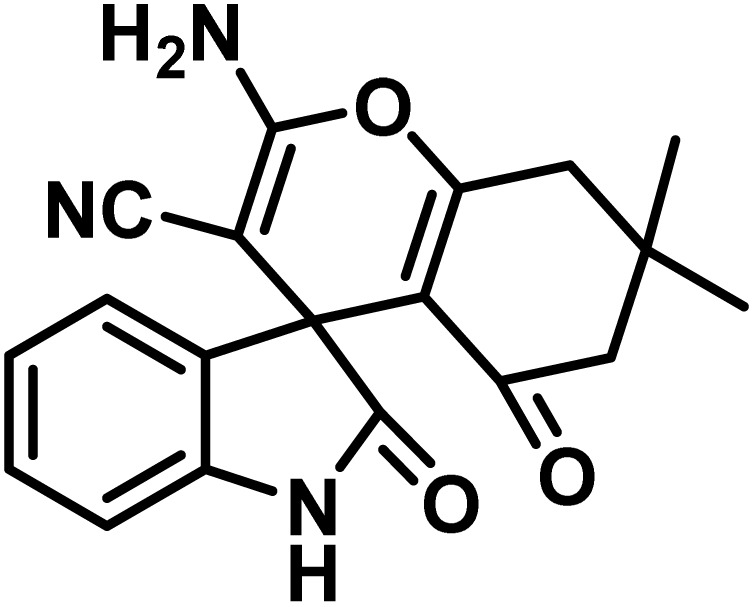	—	H_2_O/reflux	60	40	This work
	CS (32 mg)	H_2_O/reflux	60	56	This work
	CS-(M_3_SP)_2_-NH (32 mg)	H_2_O/reflux	50	83	This work
	CS-(M_3_SP)_2_-NH_2_·HSO_4_ (32 mg)	H_2_O/reflux	5	98	This work
	GN/SO_3_H (80 mg)	EtOH/H_2_O (1 : 1)/reflux	40	95	68
	Borax (10 mol%)	EtOH/reflux	120	94	69
	SSA-MNPs (100 mg)	H_2_O : EtOH/ultrasound, 60 °C	80	95	70
	HMT (10 mol%)	H_2_O/60 °C	20	95	71
	DIL@GO (200 mg)	H_2_O/reflux	10	96	72
	Fe_3_O_4_@APTPOSS (9 mg)	EtOH/r.t	45	88	73

a Isolated yields. CSA: (±)-camphor-10-sulfonic acid; *p*-TSA: *p*-toluene sulfonic acid; CAN: ceric ammonium nitrate; SSA-MNPs: silica sulfuric acid magnetic nanoparticles; HMT: hexamethylenetetramine; DIL@GO: graphene oxide-supported dicationic ionic liquid; Fe_3_O_4_@APTPOSS: polyhedral oligomeric silsesquioxanes magnetic nanoparticle.

**Fig. 10:**
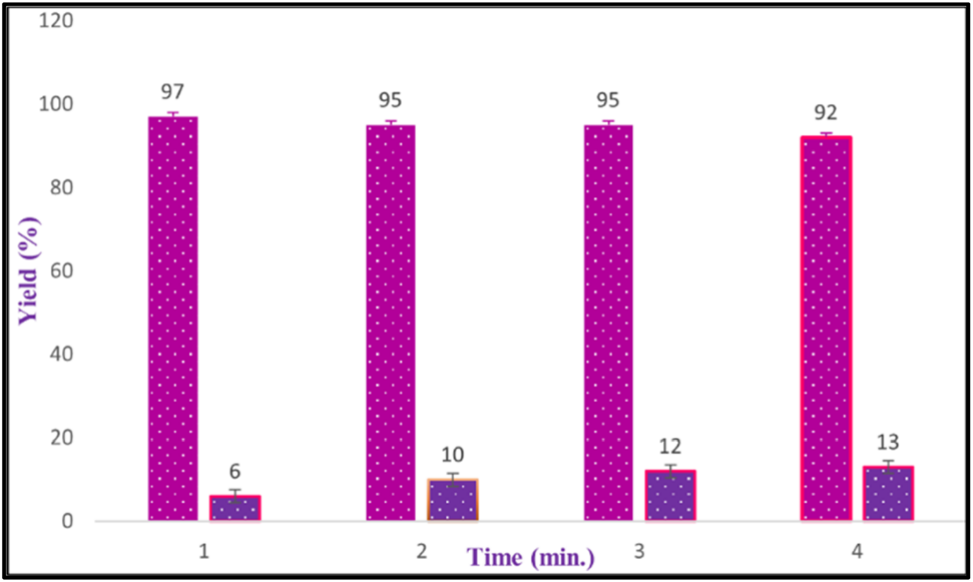
The reusability of CS-(M_3_SP)_2_-NH_2_·HSO_4_.

**Fig. 11:**
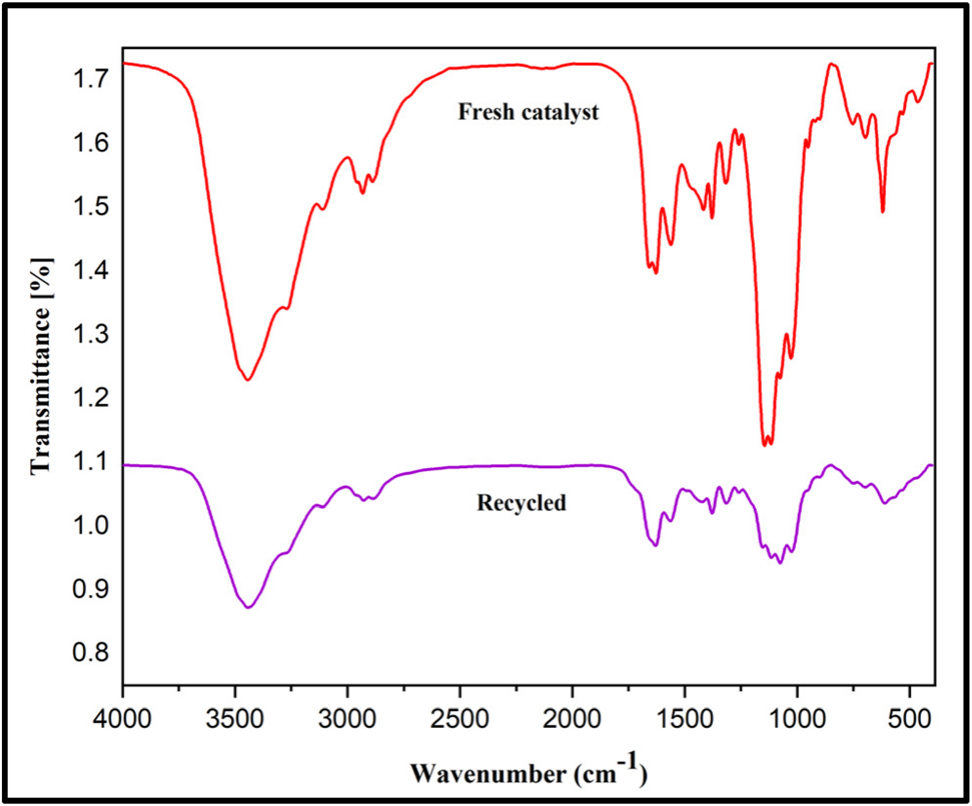
Comparison of the FTIR spectra of fresh and recycled catalyst after four runs.

**Fig. 12:**
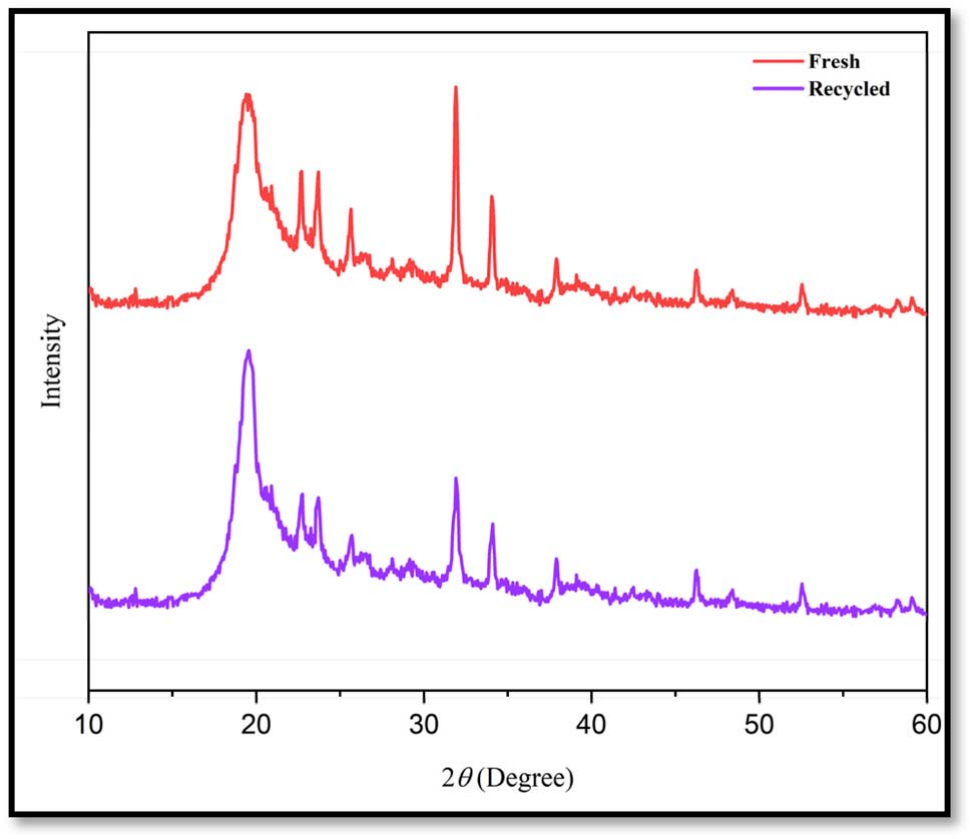
Comparison of the XRD patterns of fresh and recycled catalysts after four runs.

**Fig. 13:**
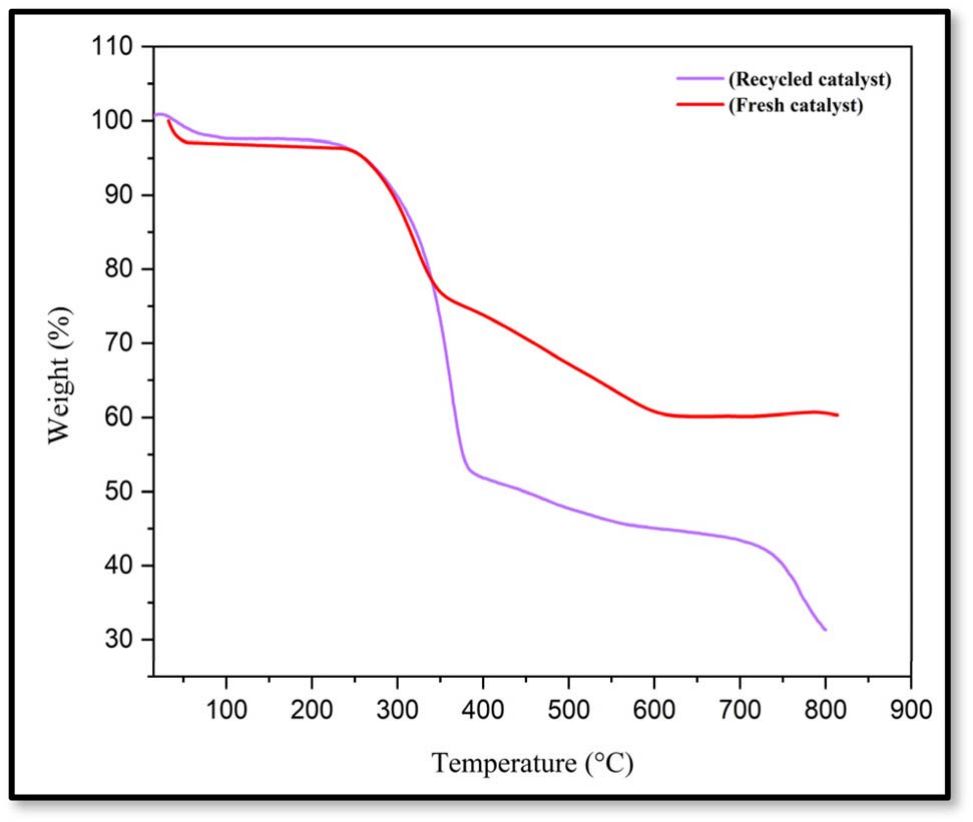
Comparison of the TGA curves of fresh and recycled catalysts after four runs.

**Fig. 14:**
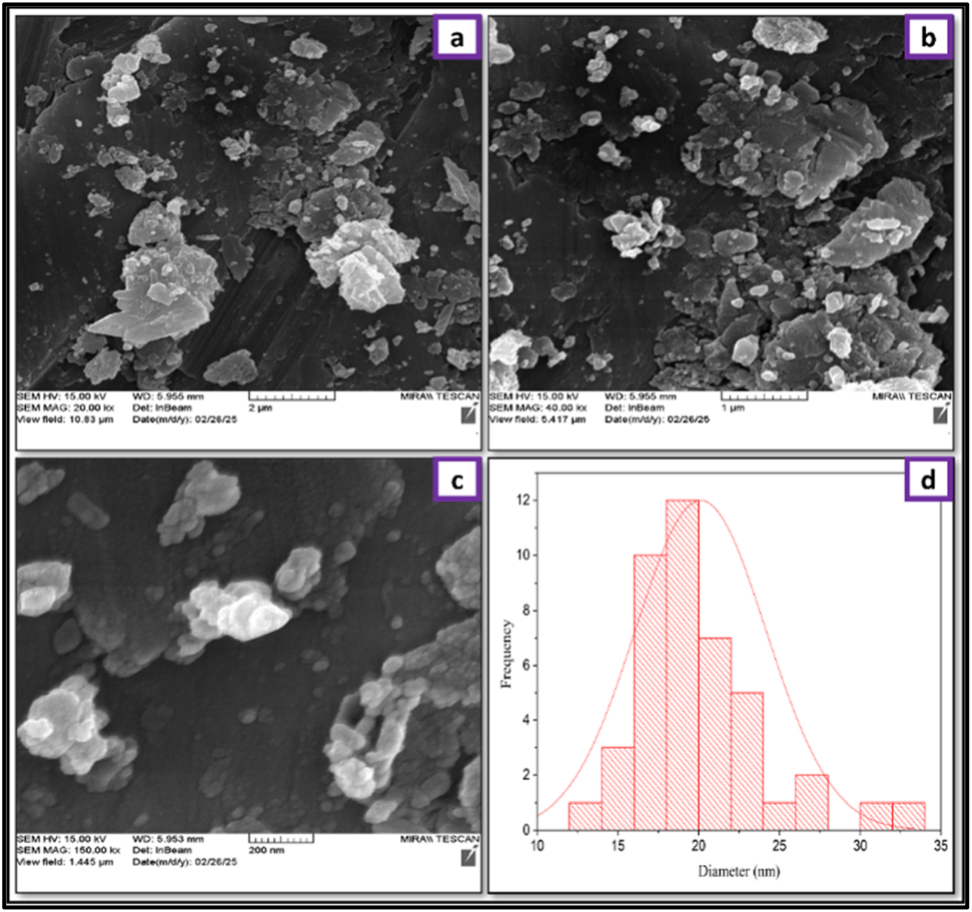
The FESEM images and the size distribution histogram of the recycled catalyst.

To check the reusability of the CS-(M_3_SP)_2_-NH_2_·HSO_4_ catalyst, the reaction of dimedone, isatin and malononitrile was re-examined as a sample one under the optimal conditions. For this purpose, the catalyst was separated and after washing with ethanol and drying at room temperature was used in the next run. Fig. 10 shows that the catalyst could be reused for at least four times without significant change in reaction times and yields of the products. Also, a comparison of the FT-IR, XRD, TGA and FESEM of the synthesized and recovered catalyst confirmed the structural stability of the catalyst under the applied conditions (Fig. 11–14).

In order to evaluate the heterogeneity and stability of the catalyst in an effective reaction, a hot filtration test was performed. For this purpose, a model reaction was initiated using 1,3-cyclohexanedione (1 mmol), isatin (1 mmol), malononitrile (1.1 mmol), and CS-(M_3_SP)_2_-NH_2_·HSO_4_ (32 mg) as a catalyst in EtOH (3 mL) at reflux. After 5 minutes, when the conversion reached 40%, the reaction mixture was rapidly filtered at high temperature to remove the catalyst from the system. The filtered solution was then stirred under the same conditions as the control reaction but without the catalyst. After an additional 15 minutes, the reaction yield in this system reached about 60%, while in the control reaction where the catalyst remained in the medium, the final efficiency increased to 95%.

**Fig. 15:**
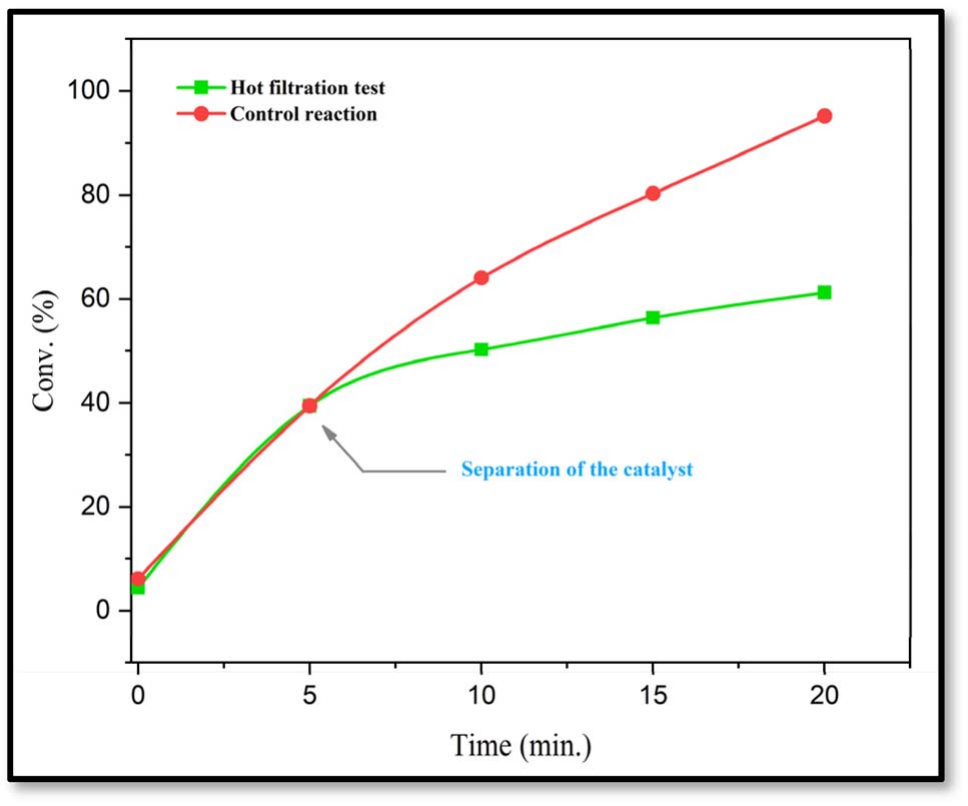
Hot filtration test to investigate the heterogeneous nature of the catalyst.

The results (Fig. 15) demonstrated that after catalyst removal, the reaction rate significantly decreased. This indicated the effective heterogeneity of the catalyst and its non-dissolution into the reaction phase. Therefore, CS-(M_3_SP)_2_-NH_2_·HSO_4_ acted as a stable heterogeneous catalyst in this reaction system and could be easily recovered from the reaction medium.

## Conclusions

In this research, an acidic ionic liquid bridge immobilized on chitosan formulated as CS-(M_3_SP)_2_-NH_2_·HSO_4_ was synthesized from readily available starting materials and was characterized with a variety of techniques. This new heterogeneous acidic reagent was applied as an efficient, cost-effective, and environmentally friendly catalyst for promoting the synthesis of (±)-spiro[indoline-3,4′-pyrazolo[3,4-*b*]quinoline]dione and (±)-spiroindoline-pyrano[2,3-*c*]quinolone derivatives. Noteworthy advantages of this method included mild reaction conditions, straightforward work-up procedures, short reaction times, and high product yields. Additionally, this catalyst was successfully recovered and recycled for at least four cycles without significant loss of activity.

## Conflicts of interest

The authors declare that they have no conflict of interest.

## Supplementary Material

RA-015-D5RA03286E-s001

## Data Availability

All relevant data supporting the findings of this study are available within the article and its supplementary information (SI). Supplementary information: ^1^H NMR & ^13^C NMR of products. See DOI: https://doi.org/10.1039/d5ra03286e.
