# Supplementary material for: Promoted synthesis of spirooxindoles in the presence of chitosan containing an acidic ionic liquid bridge in aqueous medium
Source: RSC Adv. 2025 Nov 24;15(54):45889–908. doi: 10.1039/d5ra03286e (PMC12641388; doi:10.1039/d5ra03286e)
Supplement: RA-015-D5RA03286E-s001 [file RA-015-D5RA03286E-s001.pdf]

Supplementary Information (SI) for RSC Advances.
This journal is © The Royal Society of Chemistry 2025

Electronic Supplementary Information

Promoted Synthesis of Spirooxindoles in the Presence of Chitosan Containing an Acidic Ionic Liquid Bridge in Aqueous Medium

Mohadeseh Amiri, Narges Seyedi, Farhad Shirini* and Hassan Tajik [

The spectral data of the selected compounds are as follow:

(±)-3',7',7'-Trimethyl-1'-phenyl-1',7',8',9'-tetrahydrospiro[indoline-3,4'-pyrazolo[3,4-*b*]quinoline]-2,5'(6'*H*)-dione (1a; Fig. S1 and Fig. S2): m.p.= 240-242 °C., ^{1}H NMR (400 MHz, DMSO-d_6) δ= 10.33 (s, 1H), 9.70 (s, 1H), 7.42-7.57 (m, 5H), 7.07-7.15 (m, 1H), 6.80-6.86 (m, 3H), 2.56-2.60 (m, 2H), 1.98-2.13 (m, 2H), 1.57 (s, 3H), 1.00-1.03 (d, 6H) ppm; ^{13}CNMR (100 MHz) δ= 193.3, 179.5, 153.0, 144.9, 141.7, 137.8, 136.6, 129.4, 127.1, 123.4, 121.4, 108.5, 107.9, 101.5, 50.4, 48.7, 40.9, 32.1, 28.1, 26.9, 11.3 ppm.

(±)-3'-Methyl-1'-phenyl-1',7',8',9'-tetrahydrospiro[indoline-3,4'-pyrazolo[3,4-*b*]quinoline]-2,5'(6'*H*)-dione (1b; Fig. S3 and Fig. S4): m.p.= 298-300 °C., ^{1}H NMR (400 MHz, DMSO-d_6) δ= 10.33 (s, 1H), 9.75 (s, 1H), 7.42-7.56 (m, 5H), 7.09-7.13 (m, 1H), 6.79-6.86 (m, 3H), 2.69-2.73 (m, 2H), 2.13-2.17 (m, 2H), 1.87-1.91 (m, 2H), 1.56 (s, 3H) ppm; ^{13}CNMR (100 MHz) δ= 193.5, 179.6, 154.9, 144.9, 141.7, 137.8, 137.01, 136.5, 129.4, 127.2, 123.4, 121.4, 109.2, 108.4, 101.5, 48.8, 36.9, 27.6 21.0, 11.3 ppm.

(±)-3′-Methyl-1′-phenylspiro[indoline-3,4′-pyrazolo[4′,3′:5,6]pyrido[2,3-*d*]pyrimidine]-2,5′,7′ (6′*H*,8′*H*,9′*H*)-trione (1c; Fig. S5 and Fig. S6): m.p. >300 °C., ^{1}H NMR (300 MHz, DMSO-d_6) δ= 10.77 (s, 1H, NH) ppm, 10.50 (s, 1H, NH), 10.21 (s, 1H, NH), 9.32 (s, 1H, NH), 7.41–7.62 (m, 5H, Ar–H), 7.15 (t, 1H, *J*= 7.5 Hz, Ar–H), 6.97 (d, 1H, *J*= 7.2 Hz, Ar–H), 6.90 (d, 1H, *J*= 7.5 Hz, Ar–H), 6.85 (d, 1H, *J*= 7.8 Hz, Ar–H), 1.57 (s, 3H, CH_3); ^{13}C NMR (75 MHz) δ= 178.9, 162.2, 149.7, 146.7, 145.2, 142.0, 137.7, 136.0, 135.8, 129.9, 127.9, 127.5, 123.8, 122.7, 121.8, 108.9, 100.4, 87.3, 47.8, 11.4 ppm.

Department of Organic Chemistry
Faculty of Chemistry
University of Guilan, Rasht, 41335-19141, Iran.
E-mail: shirini@guilan.ac.ir
fshirini@gmail.com

(±)-3′,6′,8′-Trimethyl-1′-phenyl-1′,9′-dihydrospiro[indoline-3,4′-pyrazolo[4′,3′:5,6]pyrido[2,3-d]pyrimidine]-2,5′,7′(6′H,8′H)-trione (1d; Fig. S7 and Fig. S8): m.p.= 259-261°C., ^{1}H NMR (400 MHz, DMSO-*d*$_6$) *δ*= 10.50 (*s*, 1H, NH), 9.56 (*s*, 1H, NH), 7.69 (*d*, *J* = 8.0 Hz, 2H, ArH), 7.55 (*t*, *J* = 8.0 Hz, 2H, ArH), 7.37 (*t*, *J* = 7.2 Hz, 1H, ArH), 7.15 (*t*, *J* = 7.2 Hz, 1H, ArH), 6.90–6.84 (*m*, 3H, ArH), 3.54 (*s*, 3H, CH_3), 3.05 (*s*, 3H, CH_3), 1.58 (*s*, 3H, CH_3) ppm; ^{13}C NMR (100 MHz) *δ*= 179.1, 160.5, 151.1, 145.1, 142.5, 139.0, 130.0, 128.2, 127.1, 123.9, 122.3, 122.1, 109.3, 102.3, 49.0, 31.1, 28.0, 11.8 ppm.

(±)-3′-Methyl-1′-phenyl-7′-thioxo-spiro[indoline-3,4′-pyrazolo[4′,3′:5,6]pyrido[2,3-*d*]pyrimidine]-2,5′(6′*H*,8′*H*,9′*H*)-dione (1e; Fig. S9 and Fig. S10): m.p.= 297-299 °C., ^{1}H NMR (400 MHz, DMSO-*d*$_6$) *δ*= 10.74 (*s*, 1H, NH), 10.47 (*s*, 1H, NH), 10.18 (*s*, 1H, NH), 9.29 (*s*, 1H, NH), 7.57 (*s*, 4H, ArH), 7.44 (*s*, 1H, ArH), 7.15–7.13 (*m*, 1H, ArH), 6.97 (*d*, *J* = 6.4 Hz, 1H, ArH), 6.88 (*t*, *J* = 7.2 Hz, 1H, ArH), 6.83 (*d*, *J* = 7.2 Hz, 1H, ArH), 1.56 (*s*, 3H, CH_3) ppm; ^{13}C NMR (100 MHz) *δ*= 179.2, 162.5, 150.0, 147.0, 145.5, 142.4, 138.1, 136.4, 136.2, 130.2, 128.2, 127.8, 124.1, 123.0, 122.1, 109.3, 100.8, 87.7, 48.2, 11.7 ppm.

(±)-Spiro[1*H*-pyrazolo[5,4-*b*]pyrido[5,6-*c*]chromene-4,3-indoline]-4*H*,5*H*,11*H*-3-methyl-1-phenyl-5,2′-dione (1f; Fig. S11 and Fig. S12): m.p.= 269-271 °C., ^{1}HNMR (400MHz, DMSO-*d*$_6$) *δ*= 12.05 (1H, s, NH), 9.39 (1H, s, NH), 8.13 (1H, d, *J*= 8.0 Hz), 7.78 (1H, t, *J*= 7.8 Hz), 7.63-7.52 (6H, m), 7.42 (1H, t, *J*= 6.6 Hz), 7.19 (2H, d, *J*= 3.6 Hz), 6.89 (1H, d, *J*= 7.6 Hz), 6.83-6.79 (1H, m), 1.79 (3H, s, CH_3) ppm; ^{13}CNMR (100 MHz) *δ*= 181.9, 181.8, 156.2, 155.3, 152.7, 145.3, 140.6, 140.5, 139.2, 138.9, 133.8, 130.0, 128.8, 127.2, 127.1, 125.0, 124.9, 121.9, 118.7, 117.9, 117.7, 111.5, 109.8, 94.4, 12.1 ppm.

(±)-5-Chloro-3',7',7'-trimethyl-1'-phenyl-1',7',8',9'-tetrahydrospiro[indoline-3,4'-pyrazolo[3,4-*b*]quinoline]-2,5'(6'*H*)-dione (1g; Fig. S13 and Fig. S14): m.p.= 294-296 °C., ^{1}H NMR (400 MHz, DMSO-*d*$_6$) *δ*= 10.34 (s, 1H), 7.76-7.80 (m, 2H), 7.44-7.48 (m, 2H), 7.27-7.31 (m, 1H), 7.07-7.09 (m, 1H), 6.77-6.81 (m, 2H), 2.46-2.50 (m, 2H), 1.92-2.04 (m, 2H), 1.56 (s, 3H), 0.98-1.00 (d, 6H) ppm; ^{13}C NMR (100 MHz) *δ*= 180.6, 128.9, 125.0, 123.0, 122.1, 99.5, 92.6, 91.5, 86.6, 81.4, 46.6, 32.0, 27.3, 11.6 ppm.

(±)-5-Chloro-3′-methyl-1′-phenylspiro[indoline-3,4′-pyrazolo[4′,3′:5,6]pyrido[2,3-*d*]pyrimidine]-2,5′,7′(6′*H*,8′*H*,9′*H*)-trione (1h; Fig. S15 and Fig. S16): m.p. >300 C., ^{1}H NMR (400 MHz, DMSO-*d*$_6$) *δ*= 10.81 (*s*, 1H, NH), 10.67 (*s*, 1H, NH), 10.22 (*s*, 1H, NH), 9.36 (*s*, 1H, NH), 7.61–7.58 (*m*, 4H, ArH), 7.46–7.42 (*m*, 1H, ArH), 7.01 (*d*, *J* = 7.6 Hz, 1H, ArH), 6.93 (*dd*, J_1 = 8.0 Hz, J_2 = 1.2 Hz, 1H, ArH), 6.87 (*s*, 1H, ArH), 1.61 (*s*, 3H, CH_3) ppm; ^{13}C NMR (100 MHz) *δ*= 179.2, 162.6, 150.0, 147.1, 145.4, 143.9, 138.1, 136.5, 135.1, 132.4, 130.2, 127.9, 125.6, 123.1, 121.8, 109.3, 100.2, 87.3, 47.9, 11.8 ppm.

(±)-5-Chloro-3′,6′,8′-trimethyl-1′-phenyl-1′,9′-dihydrospiro[indoline-3,4′-pyrazolo[4′,3′:5,6]pyrido[2,3-*d*]pyrimidine]-2,5′,7′(6′*H*,8′*H*)-trione (1i; Fig. S17 and Fig. S18): m.p.= 270–272°C., ^{1}H NMR (400MHz, DMSO-*d*$_6$) *δ*=10.67 (*s*, 1H, NH), 9.63 (*s*, 1H, NH), 7.69 (*d*, *J* = 8.0 Hz, 2H, ArH), 7.55 (*t*, *J* = 8.0 Hz, 2H, ArH), 7.38 (*t*, *J* = 7.6 Hz, 1H, ArH), 7.21 (*dd*, J_1 = 8.0 Hz, J_2 = 2.0 Hz, 1H, ArH), 6.99 (*d*, *J* = 1.6 Hz, 1H, ArH), 6.87 (*d*, *J* = 8.0 Hz, 1H, ArH), 3.53 (*s*, 3H, CH_3), 3.06 (*s*, 3H, CH_3), 1.63 (*s*, 3H, CH_3) ppm; ^{13}C NMR (100MHz) *δ*=178.9,

160.6, 151.1, 148.4, 145.0, 141.5, 138.9, 138.1, 137.3, 130.0, 128.2, 127.2, 126.1, 124.0, 122.4, 110.7, 101.5, 89.5, 49.3, 31.1, 28.0, 11.8 ppm.

(±)-Spiro[1*H*-pyrazolo[5,4-*b*]pyrido[5,6-*c*]chromene-4,3-indoline]-4*H*,5*H*,11*H*-5′-chloro-3-methyl-1-phenyl-5,2′-dione (1j; Fig. S19 and Fig. S20): m.p.= 298-300 °C., ^{1}H NMR (400MHz, DMSO-d_6) δ= 12.08 (1H, s, NH), 9.56 (1H, s, NH), 8.13 (1H, dd, *J*= 1.4 and 8.4 Hz), 7.79 (1H, dt, *J*= 1.6 and 7.6 Hz), 7.60- 7.52 (6H, m), 7.45-7.40 (1H, m, Ph), 7.37 (1H, dd, *J*= 2.0 and 8.8 Hz), 7.15 (1H, d, *J*= 8.8 Hz), 7.08 (1H, d, *J*= 2.0 Hz), 1.77 (3H, CH_3) ppm; ^{13}C NMR (100MHz) δ= 181.5, 156.3, 155.4, 153.2, 145.1, 140.2, 138.9, 138.7, 133.8, 131.8, 130.0, 129.5, 127.3, 125.2, 125.0, 123.4, 121.0, 119.8, 117.7, 112.9, 111.7, 109.2, 94.3, 51.9, 12.1 ppm.

(±)-5-bromo-3',7',7'-trimethyl-1'-phenyl-6',7',8',9'-tetrahydrospiro[indoline-3,4'-pyrazolo[3,4-*b*]quinoline]-2,5'(1'*H*)-dione (1k; Fig. S21 and Fig. S22): m.p.= 298-300 °C., ^{1}H NMR (400 MHz, DMSO-d_6) δ= 10.48 (s, 1H, NH), 9.77 (s, 1H, NH), 7.53 (s, 4H, ArH), 7.43 (d, *J*= 3.2 Hz, 1H, ArH), 7.29 (d, *J*= 8.0 Hz, 1H, ArH), 6.99 (s, 1H, ArH), 6.77-6.79 (m, 1H, ArH), 2.58 (s, 2H, CH_2), 2.07 (s, 2H, CH_3), 1.59 (s, 3H, CH_3), 1.01 (s, 3H, CH_3), 1.00 (s, 3H, CH_3) ppm. ^{13}C NMR (100MHz) δ= 12.1, 21.4, 27.9, 28.7, 32.8, 41.6, 49.5, 51.2, 102.4, 108.7, 109.0, 124.1, 124.4, 127.8, 128.2, 130.1, 130.8, 137.3, 137.8, 138.5, 140.0, 145.7, 153.6, 180.2, 194.0 ppm.

(±)-5-Bromo-3′-methyl-1′-phenyl-1′,9′-dihydrospiro[indoline-3,4′-pyrazolo[4′,3′:5,6]pyrido[2,3-*d*]pyrimidine]-2,5′,7′(6′*H*,8′*H*)-trione (1l; Fig. S23 and Fig. S24): m.p.= 285-287°C., ^{1}H NMR (400 MHz, DMSO-d_6) δ=10.80 (*s*, 1H, NH), 10.64 (*s*, 1H, NH), 10.17 (*s*, 1H, NH), 9.33 (*s*, 1H, NH), 7.62–7.57 (*m*, 4H, ArH), 7.47–7.43 (*m*, 1H, ArH), 7.34 (*dd*, J_1 = 8.4 Hz, J_2 = 2.0 Hz, 1H, ArH), 7.19 (*d*, *J* = 2.0 Hz, 1H, ArH), 6.82 (*d*, *J* = 8.4 Hz, 1H, ArH), 1.61 (*s*, 3H, CH_3) ppm; ^{13}C NMR (100 MHz) δ= 178.9, 162.6, 150.0, 147.1, 145.3, 141.7, 138.6, 138.0, 136.5, 131.0, 130.2, 127.9, 127.0, 123.3, 123.2, 113.9, 111.3, 100.1, 87.1, 48.4, 11.8 ppm.

(±)-5-Bromo-3′-methyl-1′-phenyl-7′-thioxo-spiro-[indoline-3,4′-pyrazolo[4′,3′:5,6]pyrido[2,3-*d*]pyrimidine]-2,5′(6′*H*,8′*H*,9′*H*)-dione (1m; Fig. S25 and Fig. S26): m.p. >300 °C., ^{1}H NMR (300 MHz, DMSO-d_6) $\delta_=$ 12.05 (s, 1H, NH), 11.90 (s, 1H, NH), 10.72 (s, 1H, NH), 9.35 (s, 1H, NH), 7.58–7.65 (m, 4H, Ar–H), 7.43–7.45 (m, 1H, Ar–H), 7.36 (dd, 1H, *J*= 8.4, 1.5 Hz, Ar–H), 7.26 (s, 1H, Ar–H), 6.83 (d, 1H, *J*=8.4 Hz, Ar–H), 1.67 (s, 3H, CH_3) ppm; ^{13}C NMR (75 MHz) δ= 178.1, 173.6, 159.7, 146.2, 145.1, 141.3, 137.7, 137.4, 135.6, 130.9, 129.9, 127.7, 126.9, 122.7, 113.7, 111.0, 99.5, 90.8, 48.0, 11.4 ppm.

(±)-3′,7′,7′-Trimethyl-1′-phenyl-6′,7′,8′,9′-tetra-hydro-2*H*-spiro[acenaphthylene-1,4′-pyrazolo[3,4-*b*]quinoline]-2,5′(1′*H*)-dione (1n; Fig. S27 and Fig. S28): m.p.= 294-296 °C., ^{1}H NMR (300 MHz, DMSO-d_6) $\delta_=$ 9.87 (s, 1H, NH), 8.23 (d, 1H, *J*= 8.1 Hz, Ar–H), 8.08 (d, 1H, *J*= 6.9 Hz, Ar–H), 7.87 (d, 1H, *J*= 8.7 Hz, Ar–H), 7.82 (d, 1H, *J*= 7.2 Hz, Ar–H), 7.52–7.62 (m, 5H, Ar–H), 7.41–7.43 (m, 1H, Ar–H), 7.24 (d, 1H, *J*= 6.6 Hz, Ar–H), 2.63 (br s, 2H, 8–CH_2), 2.05 (d, 1H, *J*= 16.2 Hz, H-6b), 1.94 (d, 1H, *J*= 16.2 Hz, H-6a), 1.02 (s, 3H, CH_3), 1.00 (s, 3H, CH_3), 0.94 (s, 3H, CH_3) ppm; ^{13}C NMR (75 MHz) δ= 205.0, 193.9, 153.8, 145.9, 145.1, 139.9, 137.9, 136.9, 134.0, 131.0, 129.7, 129.5, 129.2, 128.6, 127.6, 123.9, 121.5, 120.9, 120.0, 109.6, 103.1, 53.7, 50.1, 41.0, 32.4, 28.3, 27.2, 12.2 ppm.

(±)-3′-Methyl-1′-phenyl-spiro[acenaphthylene-3,4′-pyrazolo[4′,3′:5,6]pyrido[2,3-*d*]pyrimidine]-2,5′,7′(6′*H*,8′*H*,9′*H*)-trione (1o; Fig. S29 and Fig. S30): m.p. >300 °C., ^{1}H NMR (300 MHz, DMSO-d_6) δ= 10.73 (s, 1H, NH), 10.29 (s, 1H, NH), 9.42 (s, 1H, NH), 8.27 (d, 1H, J= 8.1 Hz, Ar–H), 7.99 (d, 1H, J= 6.9 Hz, Ar–H), 7.92 (d, 1H, J= 8.4 Hz, Ar–H), 7.85 (d, 1H, J= 6.9 Hz, Ar–H), 7.57–7.66 (m, 4H, Ar–H), 7.43–7.45 (m, 1H, Ar–H), 7.35 (d, 1H, J= 6.9 Hz, Ar–H), 0.93 (s, 3H, CH_3), ppm; ^{13}C NMR (75 MHz) δ= 204.9, 162.9, 150.0, 147.1, 145.3, 144.8, 140.8, 137.9, 136.3, 133.3, 131.8, 130.2, 129.8, 129.4, 128.9, 127.8, 124.5, 123.0, 121.6, 120.7, 101.8, 88.7, 52.8, 12.3 ppm.

(±)-2-Amino-7,7-dimethyl-2′,5-dioxo-5,6,7,8-tetrahydrospiro[chromene-4,3′-indoline]-3-carbonitrile (2a; Fig. S31 and Fig. S32): m.p.= 296-298 °C., ^{1}H NMR (400 MHz, DMSO-d_6) δ= 10.54 (s, 1H, NH), 7.31 (s, 2H, NH_2), 7.28 (dd, J= 8.2, 2.1 Hz, 1H, Ar-H), 7.17 (d, J= 2.0 Hz, 1H, Ar-H), 6.72 (d, J= 8.2 Hz, 2H, Ar-H), 2.59 – 2.47 (m, 2H, CH_2), 2.11 (d, J= 3.1 Hz, 2H, CH_2), 0.98 (s, 6H, CH_3) ppm; ^{13}C NMR (100 MHz) δ= ^{13}C NMR (101 MHz) δ= 195.7, 178.2, 165.2, 159.4, 141.9, 137.3, 131.5, 126.5, 117.8, 113.9, 111.7, 110.7, 57.2, 50.4, 47.5, 32.5, 28.0, 27.7 ppm.

(±)-2-Amino-2',5-dioxo-5,6,7,8-tetrahydrospiro[chromene-4,3'-indoline]-3-carbonitrile (2b; Fig. S33 and Fig. S34): m.p.= 281-283 °C., ^{1}H NMR (400 MHz, DMSO-d_6) δ= 10.53 (s, 1H, NH), 7.30 (s, 2H, NH_2), 7.27 (d, J= 2.0 Hz, 1H, Ar-H), 7.22 (d, J= 1.8 Hz, 1H, Ar-H), 6.71 (d, J= 8.2 Hz, 1H, Ar-H), 2.61 (t, J= 6.0 Hz, 2H, CH_2), 2.20 (t, J= 6.4 Hz, 1H, CH_2), 1.95 – 1.83 (m, 1H, CH_2) ppm; ^{13}C NMR (100 MHz) δ= 195.8, 178.3, 167.2, 159.2, 141.8, 137.5, 131.4, 126.7, 117.8, 113.9, 111.7, 111.6, 57.2, 47.6, 36.8, 27.3, 20.2 ppm.

(±)-7′-Amino-2,2′,4′-trioxo-1′,2′,3′,4′-tetrahydrospiro[indoline-3,5′-pyrano[2,3-*d*]pyrimidine]-6′-carbonitrile (2c; Fig. S35 and Fig. S36): m.p.= 266-269 °C., ^{1}H-NMR (500 MHz, DMSO-d_6) δ= 12.29 (s, 1H), 11.11 (s, 1H), 10.46 (s, 1H), 7.36 (s, 2H), 7.15 7.10 (m, 2H), 6.90 (t, J = 7.4 Hz, 1H), 6.77 (d, J = 7.2 Hz, 1H) ppm; ^{13}C-NMR (126 MHz) δ= 177.5, 161.3, 158.1, 153.2, 149.1, 142.0, 133.4, 128.3, 123.7, 121.7, 116.8, 109.2, 86.7, 57.7, 46.5 ppm.

(±)-7'-Amino-2,4'-dioxo-2'-thioxo-1',2',3',4'-tetrahydrospiro[indoline-3,5'-pyrano[2,3-*d*]pyrimidine]-6'-carbonitrile (2d; Fig. S37 and Fig. S38): m.p.= 225-227 °C., ^{1}H NMR (400 MHz, DMSO-d_6) δ= 13.81 (s, 1H), 12.44 (s, 1H), 10.47 (s, 1H), 7.36 (s, 2H), 7.07 7.12 (m, 2H), 6.83 (s, 1H), 6.71 (d, 1H, J = 7.2Hz) ppm; ^{13}C NMR (100 MHz) δ= 177.1, 173.8, 159.0, 158.0, 152.7, 142.0, 132.9, 128.5, 123.9, 121.8, 116.7, 109.2, 91.5, 57.4, 46.5 ppm.

(±)-2'-Amino-2,5'-dioxo-5'*H*-spiro[indoline-3,4'-pyrano[3,2-*c*]chromene]-3'-carbonitrile (2e; Fig. S39 and Fig. S40): m.p.= 285-287 °C., ^{1}H NMR (400 MHz, DMSO-d_6) δ= 10.51 (br s, 1H, -NH), 7.98 (d, J=7.9 Hz, 1H, Ar-H), 7.65 (t, J= 7.8 Hz, 1H, Ar-H), 7.41 (t, J= 7.6 Hz, 1H, Ar-H), 7.32 (d, J= 8.3 Hz, 1H, Ar-H), 7.21 (t, J= 7.6 Hz, 1H, Ar-H), 7.07 (s, 1H, Ar-H), 7.05 (s, 2H, -NH_2), 6.99 – 6.88 (m, 2H, Ar-H) ppm; ^{13}C NMR (100 MHz) δ= 177.2, 158.5, 158.3, 155.1, 152.1, 142.2, 133.7, 133.1, 128.9, 125.0, 124.2, 122.7, 122.1, 117.0, 116.7, 112.5, 109.5, 101.4, 57.1, 47.6 ppm.

(±)-2-Amino-5'-chloro-7,7-dimethyl-2',5-dioxo-5,6,7,8-tetrahydrospiro[chromene-4,3'-indoline]-3-carbonitrile (2f; Fig. S41 and Fig. S42): m.p.= 303-305 °C., ^{1}H NMR (400 MHz, DMSO-d_6) δ= 10.56 (br s, 1H, -NH), 7.32 (s, 2H, -NH_2), 7.19 (d, J= 8.2 Hz, 1H, Ar-H), 7.09 (d, J= 2.1 Hz, 1H, Ar-H), 6.81 (d, J= 8.2 Hz, 1H, Ar-H), 2.63 – 2.51 (m, 2H, -CH_2), 2.20 – 2.09 (m, 2H, -CH_2), 1.02 (s, 6H, 2 X-CH_3) ppm; ^{13}C NMR (100 MHz) δ= 195.6, 178.3, 165.1, 159.4, 141.5, 136.9, 128.6, 126.1, 123.7, 117.7, 111.1, 110.6, 57.2, 50.4, 47.6, 32.4, 27.9, 27.7 ppm.

(±)-2-Amino-5'-chloro-2',5-dioxo-5,6,7,8-tetrahydrospiro[chromene-4,3'-indoline]-3-carbonitrile (2g; Fig. S43 and Fig. S44): m.p.= 285-287 °C., ^{1}H NMR (400 MHz, DMSO-d_6) δ= 10.55 (s, 1H), 7.32 (s, 2H), 7.17-7.20 (m, 1H), 7.15 (d, 1H, J = 2.0 Hz), 6.79 (d, 1H, J = 8.4 Hz), 2.65 (t, 2H, J = 6.0 Hz), 2.24 (t, 2H, J = 6.6 Hz), 1.88-1.99 (m, 2H) ppm; ^{13}C NMR (100 MHz) δ=195.7, 178.3, 167.0, 159.1, 141.3, 137.0, 128.4, 126.0, 123.9, 117.7, 111.6, 110.9, 57.1, 47.5, 36.7, 27.2, 20.1 ppm.

(±)-7'-Amino-5-chloro-2,2',4'-trioxo-1',2',3',4'-tetrahydrospiro[indoline-3,5'-pyrano[2,3-*d*]pyrimidine]-6'-carbonitrile (2h; Fig. S45 and Fig. S46): m.p.= 247-250 °C., ^{1}H NMR (400 MHz, DMSO-d_6) δ= 12.32 (br s, 1H, -NH), 11.16 (br s, 1H, -NH), 10.62 (br s, 1H, -NH), 7.45 (s, 2H, -NH_2), 7.34 (d, J= 2.0 Hz, 1H, Ar-H), 7.22 (dd, J= 8.2, 2.1 Hz, 1H, Ar-H), 6.81 (d, J= 8.2 Hz, 1H, Ar-H) ppm; ^{13}C NMR (100 MHz) δ= 177.9, 161.9, 158.8, 154.0, 149.7, 141.5, 136.1, 128.7, 126.3, 124.6, 117.3, 111.1, 86.7, 57.5, 47.4 ppm.

(±)-7'-Amino-5-chloro-2,4'-dioxo-2'-thioxo-1',2',3',4'-tetrahydrospiro[indoline-3,5'-pyrano[2,3-*d*] pyrimidine]-6'-carbonitrile (2i; Fig. S47 and Fig. S48): m.p.= 241-244 °C., ^{1}H NMR (400 MHz, DMSO-d_6) δ= 12.54 (s, 1H), 10.68 (s, 1H), 7.51 (s, 2H), 7.42 (d, 1H, J= 1.6 Hz), 7.23 (dd, 1H, J= 8.2, 1.6 Hz), 6.81 (d, 1H, J= 8.4) ppm; ^{13}C NMR (100 MHz) δ= 177.0, 173.9, 159.1, 158.1, 152.9, 141.0, 134.9, 128.4, 125.8, 124.3, 116.6, 110.6, 90.9, 56.7, 46.8 ppm.

(±)-2'-Amino-5-chloro-2,5'-dioxo-5'*H*-spiro[indoline-3,4'-pyrano[3,2-*c*]chromene]-3'-carbonitrile (2j; Fig. S49 and Fig. S50): m.p.= 312-314 °C., ^{1}H NMR (400 MHz, DMSO-d_6) δ= 10.84 (s, 1H), 7.93 (d, J = 8.1 Hz, 1H), 7.77 (t, J = 5.9 Hz, 3H), 7.55 (t, J = 7.6 Hz, 1H), 7.50 (d, J = 8.4 Hz, 1H), 7.45 (d, J = 2.3 Hz, 1H), 7.27 (dd, J = 8.3, 2.3 Hz, 1H), 6.87 (d, J = 8.3 Hz, 1H) ppm; ^{13}C NMR (100 MHz) δ= 177.1, 158.6, 155.5, 152.2, 141.2, 135.1, 133.8, 128.9, 126.2, 125.1, 124.7, 122.8, 117.0, 116.8, 112.6, 110.9, 100.8, 56.4, 47.9 ppm.

(±)-2-Amino-5'-bromo-7,7-dimethyl-2',5-dioxo-5,6,7,8-tetrahydrospiro[chromene-4,3'-indoline]-3-carbonitrile (2k; Fig. S51 and Fig. S52): m.p.= 296-298 °C., ^{1}H NMR (400 MHz, DMSO-d_6) δ= 10.54 (s, 1H, NH), 7.31 (s, 2H, NH_2), 7.28 (dd, J= 8.2, 2.1 Hz, 1H, Ar-H), 7.17 (d, J= 2.0 Hz, 1H, Ar-H), 6.72 (d, J= 8.2 Hz, 2H, Ar-H), 2.59 – 2.47 (m, 2H, CH_2), 2.11 (d, J= 3.1 Hz, 2H, CH_2), 0.98 (s, 6H, CH_3) ppm; ^{13}C NMR (100 MHz) δ= 195.7, 178.2, 165.2, 159.4, 141.9, 137.3, 131.5, 126.5, 117.8, 113.9, 111.7, 110.7, 57.2, 50.4, 47.5, 32.5, 28.0, 27.7 ppm.

(±)-2-Amino-5'-bromo-2',5-dioxo-5,6,7,8-tetrahydrospiro[chromene-4,3'-indoline]-3-carbonitrile (2l; Fig. S53 and Fig. S54): m.p.= 277-279 °C., ^{1}H NMR (400 MHz, DMSO-d_6) δ= 10.53 (s, 1H, NH), 7.30 (s, 2H, NH_2), 7.27

(d, *J* = 2.0 Hz, 1H, Ar-H), 7.22 (d, *J*= 1.8 Hz, 1H, Ar-H), 6.71 (d, *J*= 8.2 Hz, 1H, Ar-H), 2.61 (t, *J*= 6.0 Hz, 2H, CH_2), 2.20 (t, *J*= 6.4 Hz, 1H, CH_2), 1.95 – 1.83 (m, 1H, CH_2) ppm; ^{13}C NMR (100 MHz) *δ*= 195.8, 178.3, 167.2, 159.2, 141.8, 137.5, 131.4, 126.7, 117.8, 113.9, 111.7, 111.6, 57.2, 47.6, 36.8, 27.3, 20.2 ppm.

(±)-7'-Amino-5-bromo-2,2',4'-trioxo-1',2',3',4'-tetrahydrospiro[indoline-3,5'-pyrano[2,3-*d*]pyrimidine]-6'-carbonitrile (2m; Fig. S55 and Fig. S56): m.p.= 251-253 °C., ^{1}H NMR (400 MHz, DMSO-*d*$_6$) *δ*= 12.30 (s, 1H, NH), 11.14 (s, 1H, NH), 10.60 (s, 1H, NH), 7.43 (s, 2H, NH_2), 7.41 (d, *J*= 2.0 Hz, 1H, Ar-H), 7.30 (dd, *J*= 8.2, 2.1 Hz, 1H, Ar-H), 6.71 (d, *J*= 8.2 Hz, 1H, Ar-H) ppm; ^{13}C NMR (100 MHz) *δ*= 177.9, 162.1, 158.9, 154.1, 149.8, 141.9, 136.5, 131.6, 127.4, 117.4, 114.1, 111.7, 86.8, 57.5, 47.4 ppm.

(±)-2'-Amino-2,5-dioxo-6,8-diamino-2,5'dihydro-2*H*-spiro[acenaphthylene-1,4'-chrom-ene]-3'-carbonitrile (2n; Fig. S57 and Fig. S58): m.p.= 289-291 °C., ^{1}H NMR (300 MHz, DMSO-*d*$_6$) *δ*= 8.25 (1H, d, *J* = 7.5 Hz, ArH), 7.78-7.93 (4H, m, ArH, NH_2), 7.63 (1H, t, *J* = 7.5 Hz, ArH), 7.28-7.41 (2H, m, ArH), 2.60 (2H, s, CH_2), 2.06 (2H, t, *J* = 17.5 Hz, CH_2), 1.01 (3H, s, CH_3), 0.99 (3H, s, CH_3) ppm; ^{13}C NMR (75 MHz) *δ*= 203.5, 195.3, 164.5, 158.6, 143.1, 140.4, 132.0, 131.4, 129.7, 128.8, 128.4, 124.5, 121.3, 119.7, 117.4, 111.9, 57.8, 50.8, 49.6, 39.8, 32.0, 27.4, 27.0 ppm.

(±)-2'-Amino-2,5'-dioxo-5',6',7',8'-tetrahydro-2*H*-spiro[acenaphthylene-1,4'-chromene]-3'-carbonitrile (2o; Fig. S59 and Fig. S60): m.p.= 244-246 °C., ^{1}H NMR (300 MHz, DMSO-*d*$_6$) *δ*= 8.29 (d, *J*= 8.3 Hz, 1H, Ar), 7.90-7.96 (m, 2H, Ar), 7.84 (t, *J*= 7.8 Hz, 1H, Ar), 7.67 (t, *J*= 7.2 Hz, 1H, Ar), 7.43 (d, *J*= 7.1 Hz, 1H, Ar), 7.34 (brs, 2H, NH_2), 2.74-2.80 (m, 2H, CH_2), 2.17-2.25 (m, 2H, CH_2), 1.94-2.00 (m, 2H, CH_2) ppm; ^{13}C NMR (75 MHz) *δ*= 204.1, 195.9, 166.9, 159.1, 143.8, 140.9, 132.7, 131.9, 130.2, 129.3, 128.9, 124.9, 121.8, 120.5, 118.0, 113.6, 58.5, 51.5, 36.5, 27.2, 20.3 ppm.

(±)-7'-Amino-2,2',4'–trioxo-1',2',3',4'-tetrahydro-2*H*-spiro[acenaphthylene-1,5'-pyrano[2,3-*d*]-pyrimidine]-6'-carbonitrile (2p; Fig. S61 and Fig. S62): m.p.= 298-300 °C., ^{1}H NMR (400 MHz, DMSO-*d*$_6$) *δ*= 13.74 (brs, 1H), 12.28 (s, 1H), 8.35-8.03 (m, 2H), 7.81 (s, 2H), 7.69-7.29 (m, 4H) ppm; ^{13}C NMR (100 MHz) *δ*= 203.4, 161.6, 158.2, 153.3, 149.2, 142.2, 140.9, 131.7, 131.3, 129.7, 128.5, 128.0, 124.4, 121.2, 120.0, 116.9, 87.8, 58.3, 50.5 ppm.

(±)-7'-Amino-2,4'-dioxo-2'-thioxo-1',2',3',4'-tetrahydro-2*H*-spiro[acenaphthylene-1,5'-pyrano[2,3-*d*]pyrimidine]-6'-carbonitrile (2q; Fig. S63 and Fig. S64): m.p.= 214-216 °C., ^{1}H NMR (400 MHz, DMSO-*d*$_6$) *δ*= 13.85 (br s, 1 H, NH), 12.40 (s, 1 H, NH), 8.30 (d, *J*= 8.0 Hz, 1 H_{arom}), 7.95-7.98 (m, 2 H_{arom}), 7.83 (t, *J*= 8.0 Hz, 1 H_{arom}), 7.68 (t, *J*= 8.0 Hz, 1 H_{arom}), 7.60 (d, *J*= 6.8 Hz, 1 H_{arom}), 7.50 (s, 2 H, NH_2) ppm; ^{13}C NMR (100 MHz) *δ*= 203.9, 174.6, 160.0, 158.6, 153.7, 142.4, 141.4, 132.4, 132.0, 130.2, 129.4, 128.9, 128.9, 125.3, 122.1, 121.2, 117.5, 92.9, 58.5, 51.1 ppm.

(±)-2'-Amino-2,5'-dioxo-2*H*,5'*H*-spiro[acenaphthylene-1,4'-pyrano[3,2-*c*]chromene]-3'-carbonitrile (2r; Fig. S65 and Fig. S66): m.p.= 298-300 °C., ^{1}H NMR (300 MHz, DMSO-*d*$_6$) *δ*= 8.37 (d, *J*= 8.0 Hz, 1H, Ar), 8.00-8.07 (m, 3H, Ar), 7.90 (t, *J*= 7.6 Hz, 1H, Ar), 7.64-7.83 (m, 5H, Ar, NH_2), 7.59 (t, *J*= 7.6 Hz, 1H, Ar), 7.51 (d, *J*= 8.0 Hz,

1H, Ar) ppm; ^{13}C NMR (75 MHz) δ = 203.6, 159.4, 158.9, 155.9, 152.6, 142.2, 141.6, 134.1, 132.7, 131.9, 130.32, 129.5, 129.2, 125.7, 125.6, 123.2, 122.6, 113.0, 102.7, 58.1, 52.0 ppm.

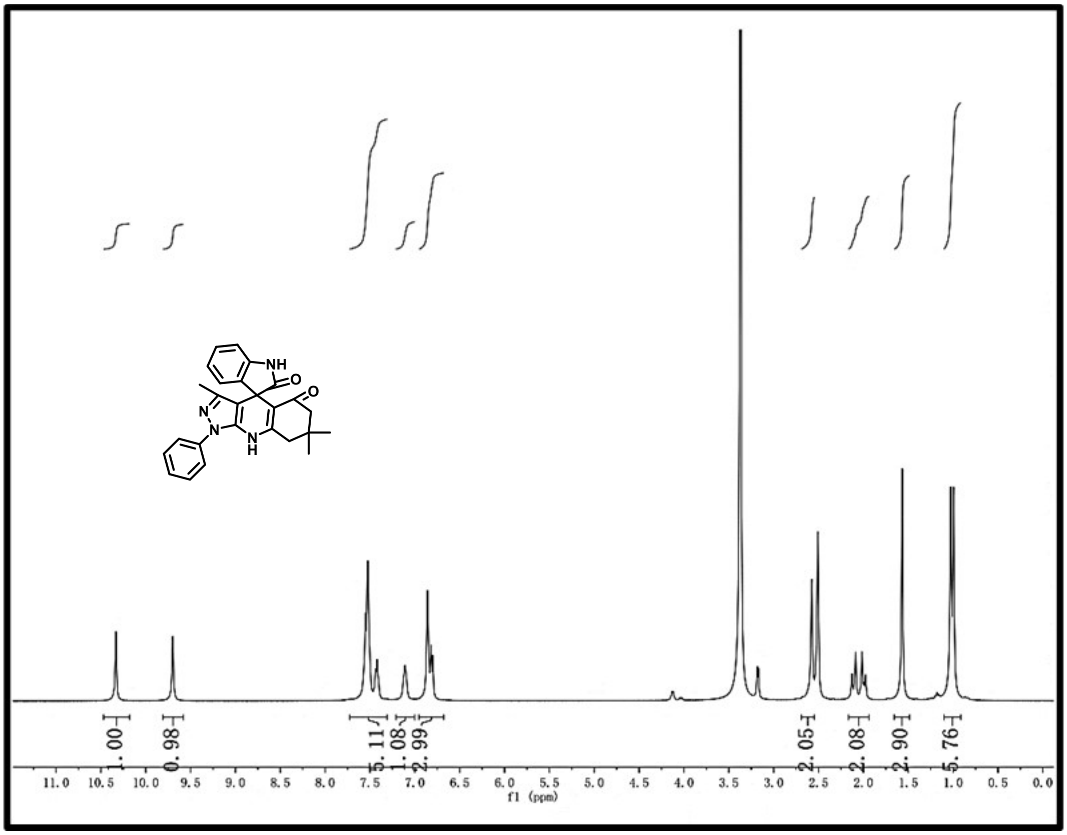

Fig. S1. ^{1}H NMR of (±)-3',7',7'-Trimethyl-1'-phenyl-1',7',8',9'-tetrahydrospiro[indoline-3,4'-pyrazolo[3,4-*b*]quinoline]-2,5'(6'*H*)-dione (1a).

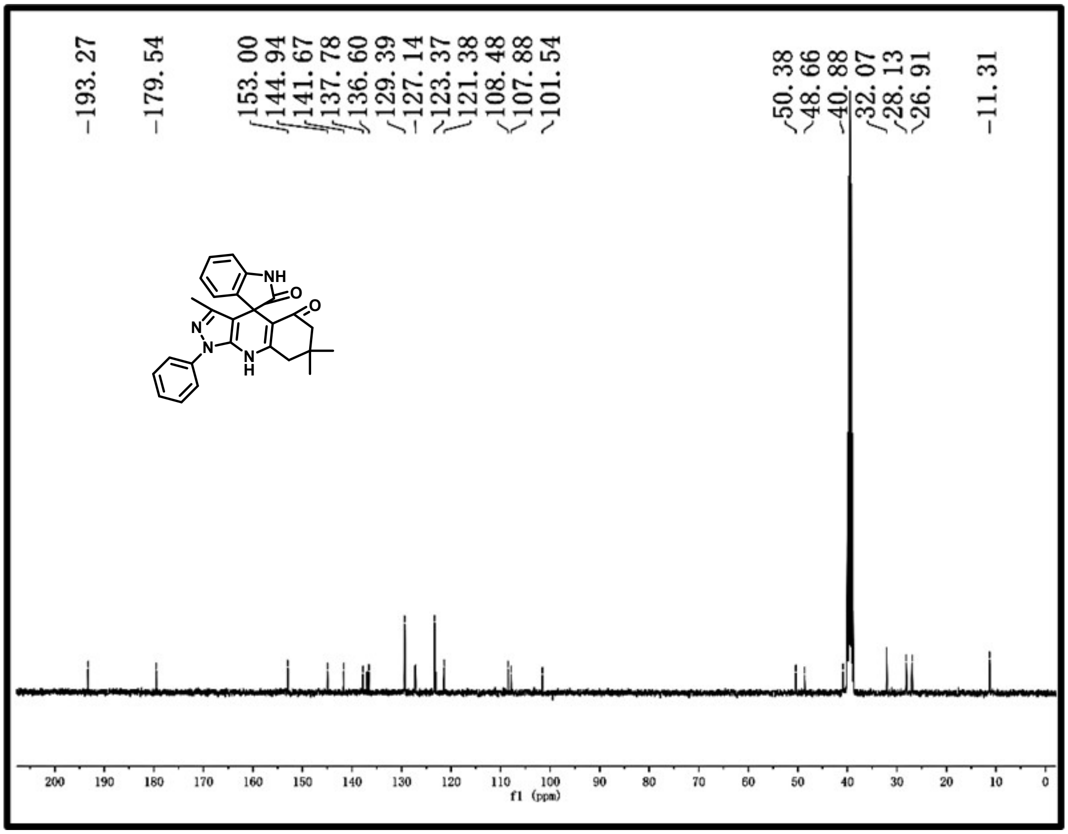

Fig. S2. ^{13}C NMR of (±)-3',7',7'-Trimethyl-1'-phenyl-1',7',8',9'-tetrahydrospiro[indoline-3,4'-pyrazolo[3,4-*b*]quinoline]-2,5'(6'*H*)-dione (1a).

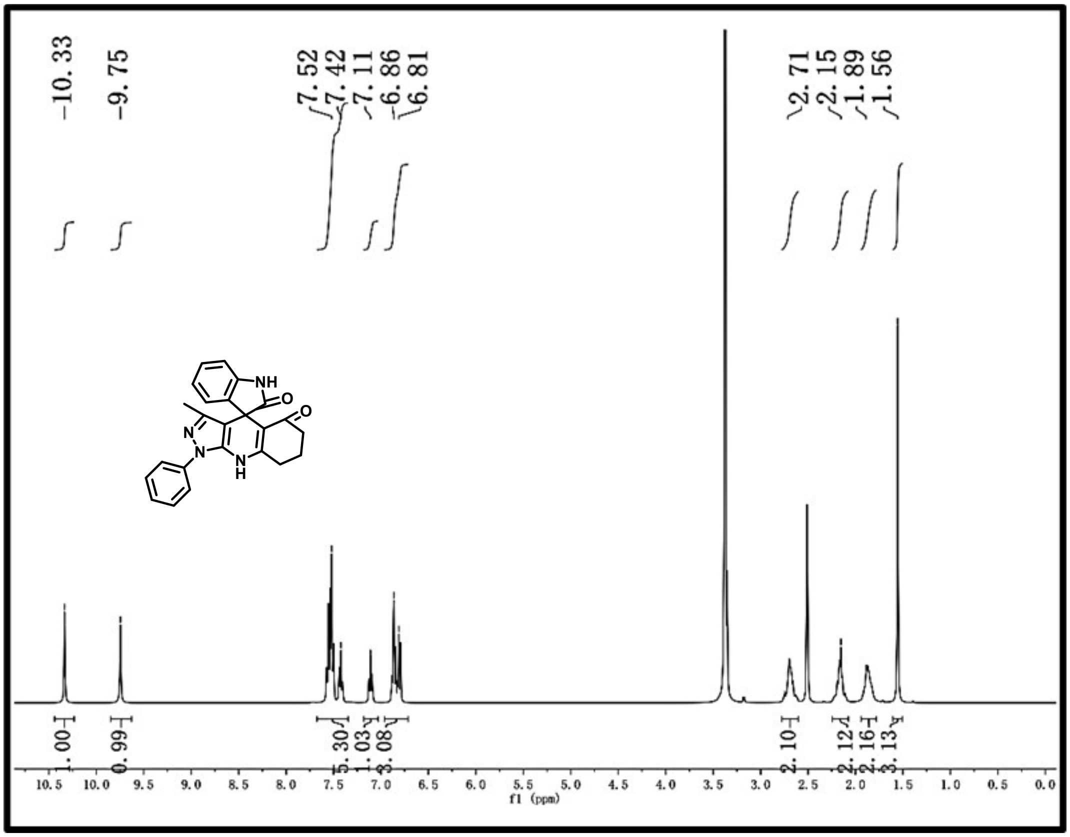

Fig. S3. ^{1}H NMR of (±)-3'-Methyl-1'-phenyl-1',7',8',9'-tetrahydrospiro[indoline-3,4'-pyrazolo[3,4-*b*]quinoline]-2,5'(6'*H*)-dione (1b).

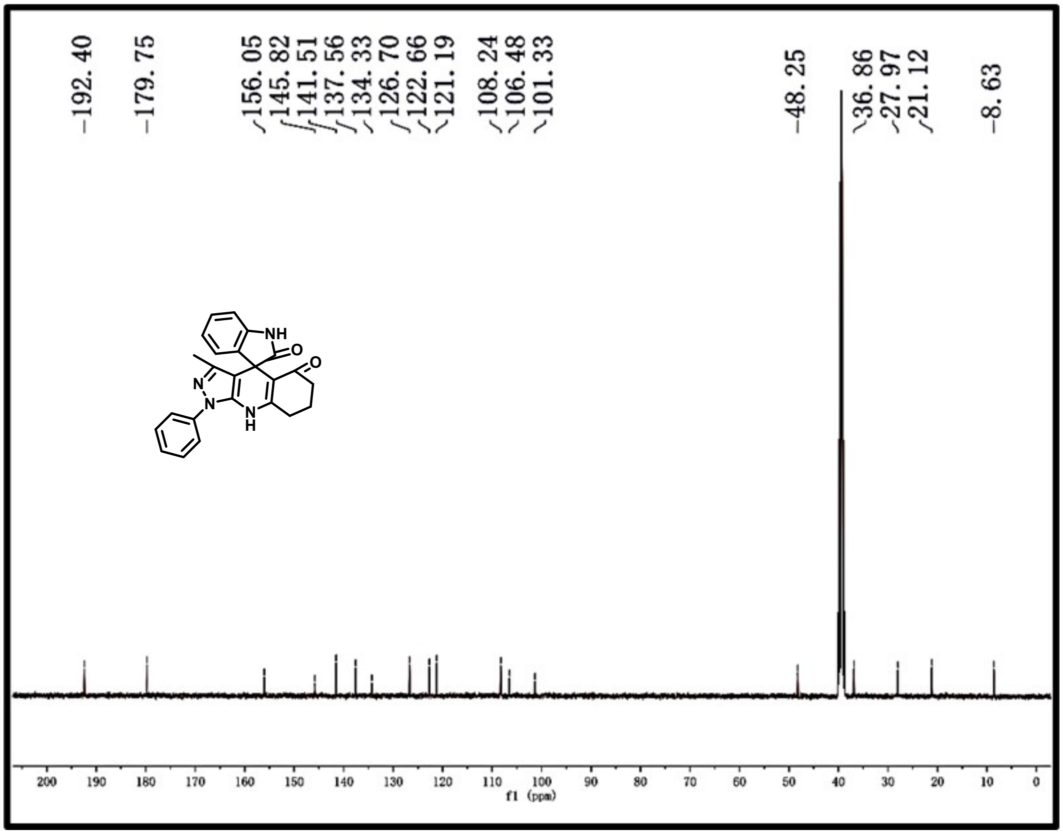

Fig. S4. ^{13}C NMR of (±)-3'-Methyl-1'-phenyl-1',7',8',9'-tetrahydrospiro[indoline-3,4'-pyrazolo[3,4-*b*]quinoline]-2,5'(6'*H*)-dione (1b).

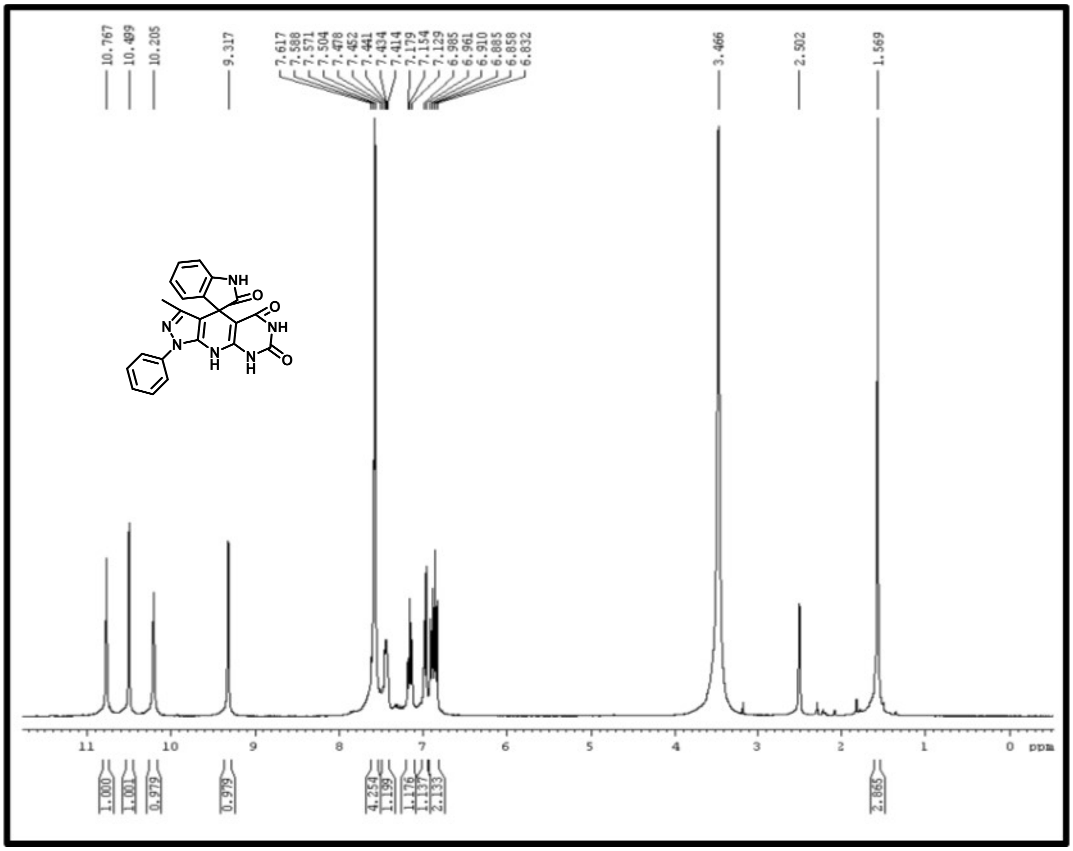

Fig. S5. ^{1}H NMR of (±)-3′-Methyl-1′-phenylspiro[indoline-3,4′-pyrazolo [4′,3′:5,6]pyrido[2,3-*d*]pyrimidine]-2,5′,7′(6′*H*,8′*H*,9′*H*)-trione (1c).

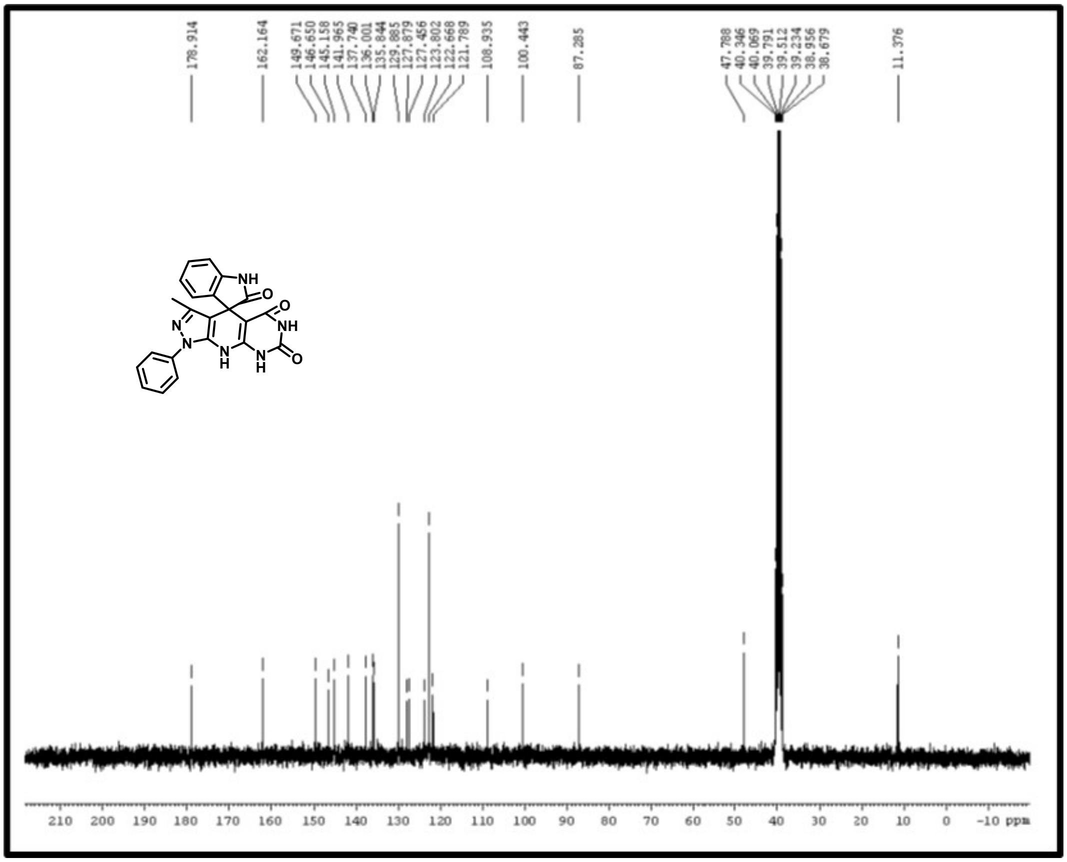

Fig. S6. ^{13}C NMR of (±)-3′-Methyl-1′-phenylspiro[indoline-3,4′-pyrazolo [4′,3′:5,6]pyrido[2,3-*d*]pyrimidine]-2,5′,7′(6′*H*,8′*H*,9′*H*)-trione (1c).

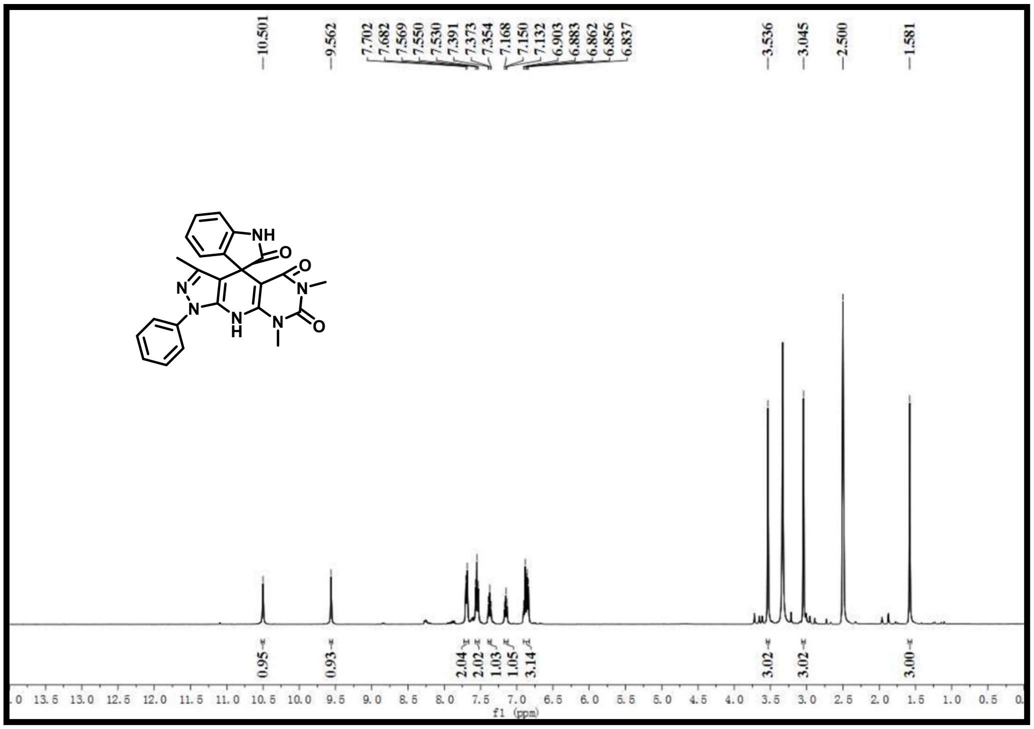

Fig. S7. ^{1}H NMR of (±)-3′,6′,8′-Trimethyl-1′-phenyl-1′,9′-dihydrospiro[indoline-3,4′-pyrazolo[4′,3′:5,6]pyrido[2,3-*d*]pyrimidine]-2,5′,7′(6′*H*,8′*H*)-trione (1d).

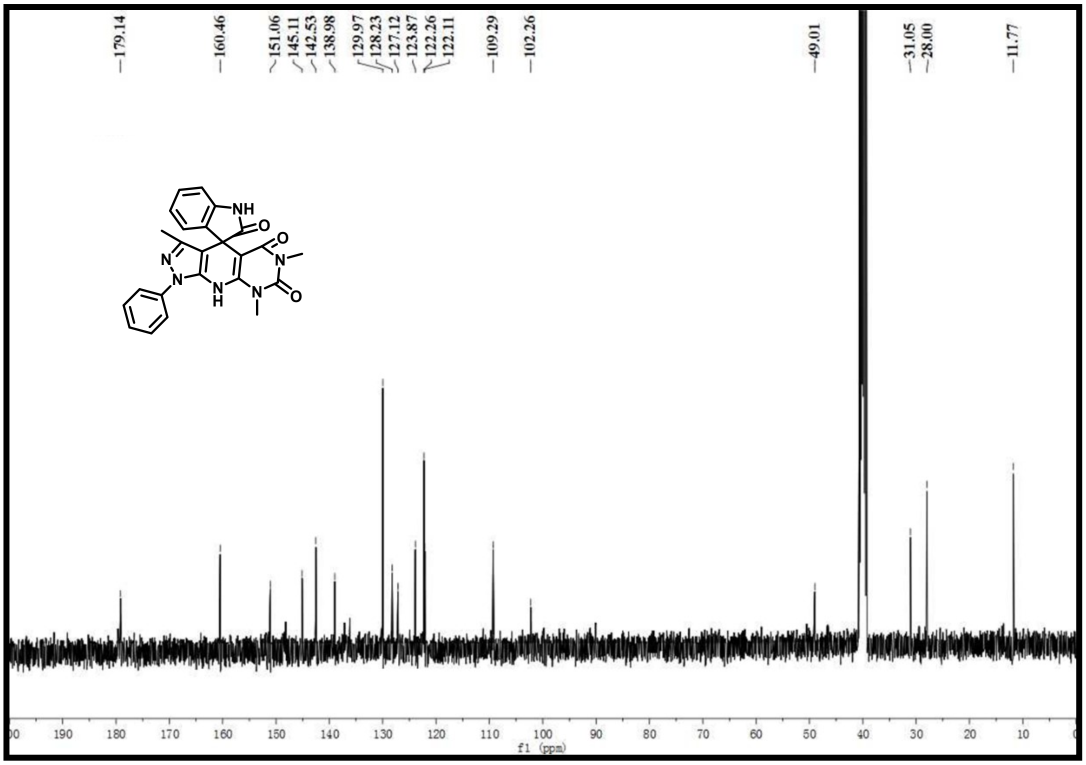

Fig. S8. ^{13}C NMR of (±)-3′,6′,8′-Trimethyl-1′-phenyl-1′,9′-dihydrospiro[indoline-3,4′-pyrazolo[4′,3′:5,6]pyrido[2,3-*d*]pyrimidine]-2,5′,7′(6′*H*,8′*H*)-trione (1d).

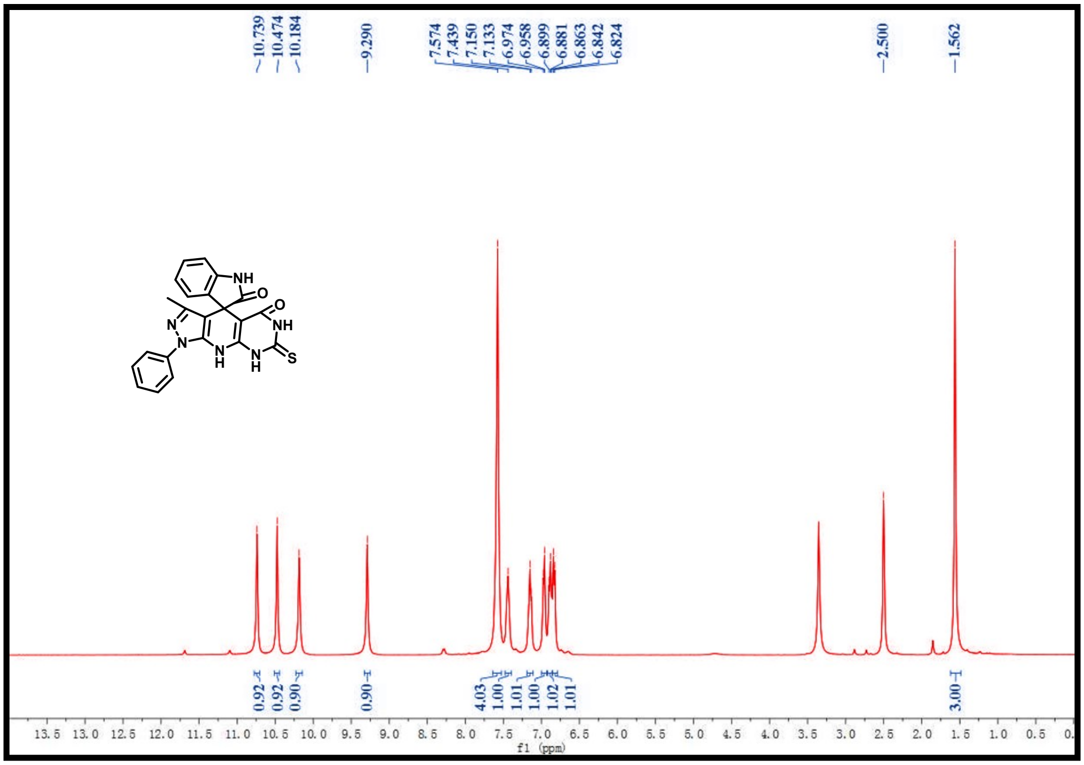

Fig. S9. ¹H NMR of (±)-3′-Methyl-1′-phenyl-7′-thioxo-spiro[indoline-3,4′-pyrazolo[4′,3′:5,6]pyrido[2,3-*d*]pyrimidine]-2,5′(6′*H*,8′*H*,9′*H*)-dione (1e).

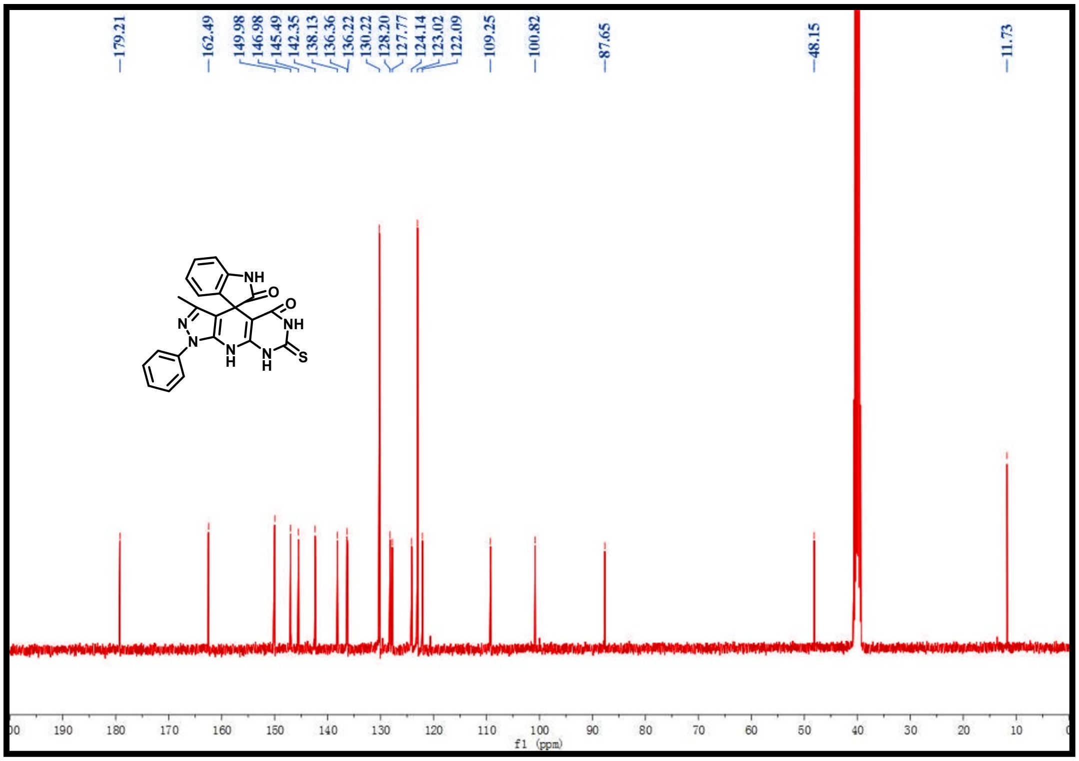

Fig. S10. ^{13}C NMR of (±)-3′-Methyl-1′-phenyl-7′-thioxo-spiro[indoline-3,4′-pyrazolo[4′,3′:5,6]pyrido[2,3-*d*]pyrimidine]-2,5′(6′*H*,8′*H*,9′*H*)-dione (1e).

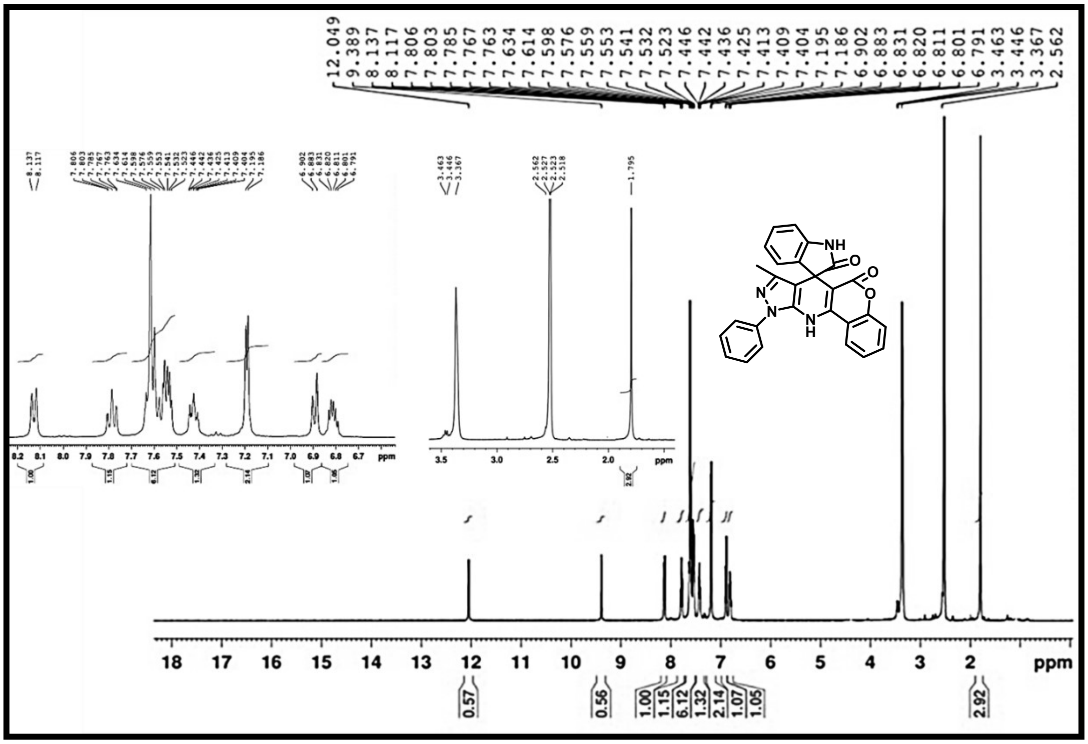

Fig. S11. ^{1}H NMR of (±)-Spiro[1*H*-pyrazolo[5,4-*b*]pyrido[5,6-*c*]chromene-4,3-indoline]-4*H*,5*H*,11*H*-3-methyl-1-phenyl-5,2′-dione (1f).

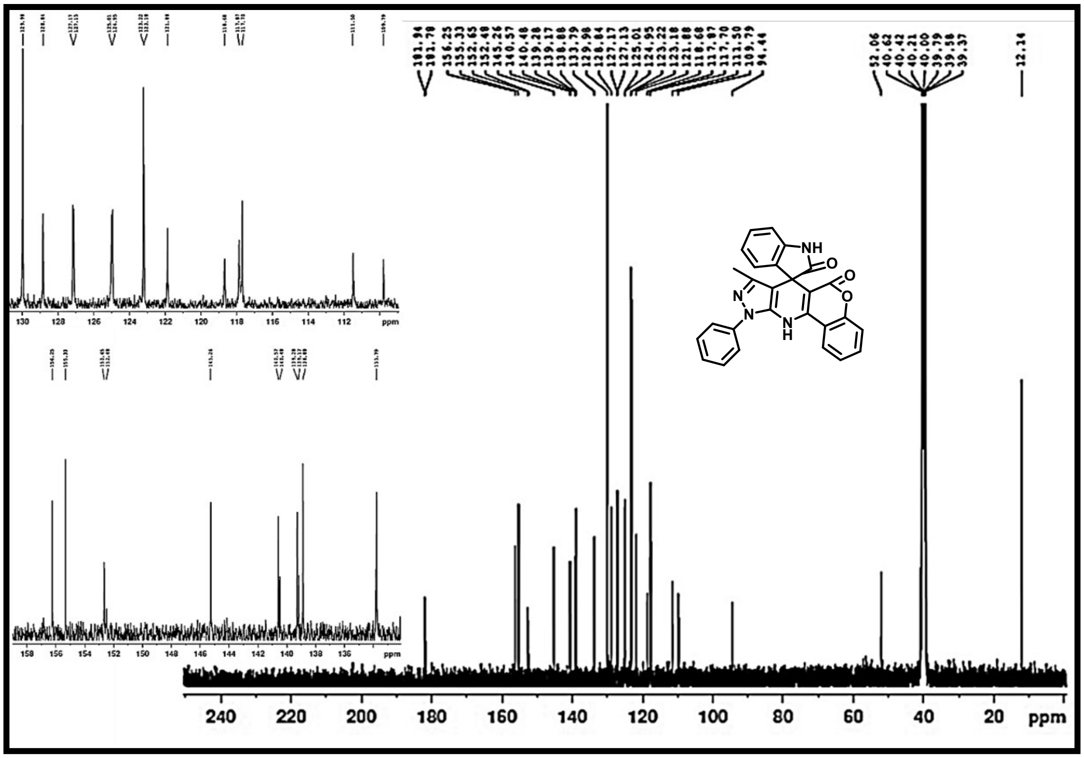

Fig. S12. ^{13}C NMR of (±)-Spiro[1*H*-pyrazolo[5,4-*b*]pyrido[5,6-*c*]chromene-4,3-indoline]-4*H*,5*H*,11*H*-3-methyl-1-phenyl-5,2′-dione (1f).

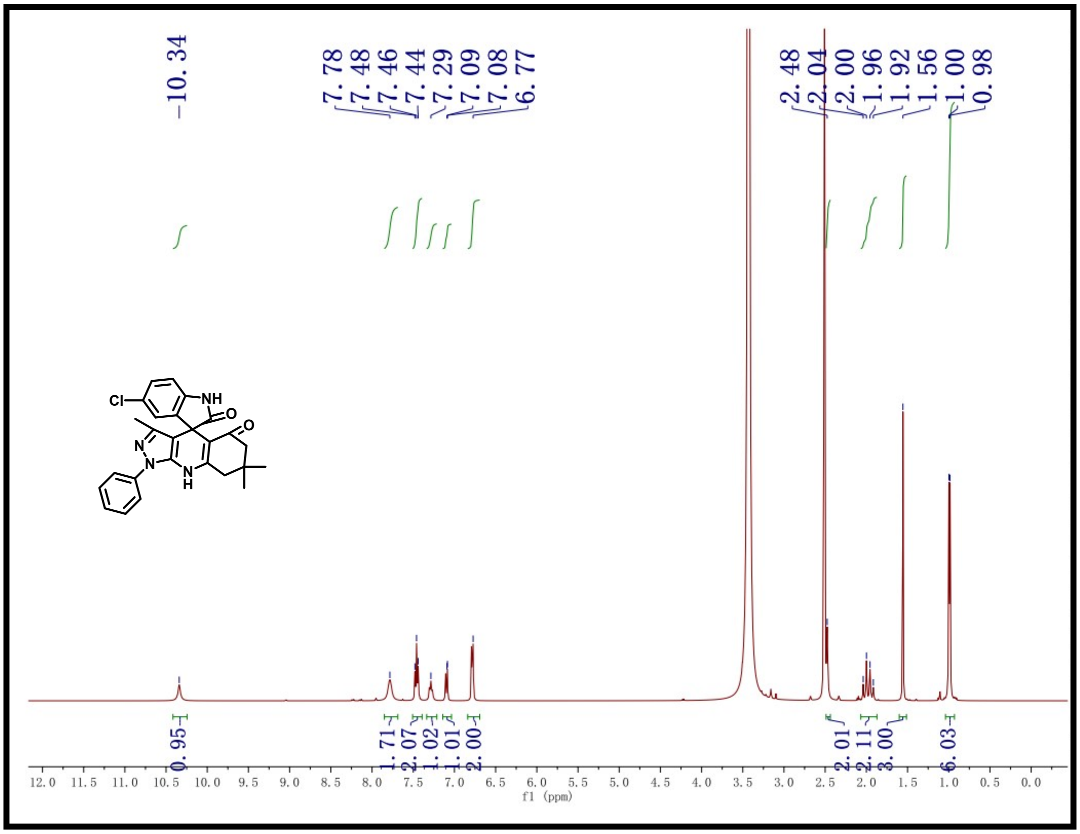

Fig. S13. ^{1}HNMR of (±)-5-Chloro-3',7',7'-trimethyl-1'-phenyl-1',7',8',9'-tetrahydrospiro[indoline-3,4'-pyrazolo[3,4-*b*]quinoline]-2,5'(6'*H*)-dione (1g).

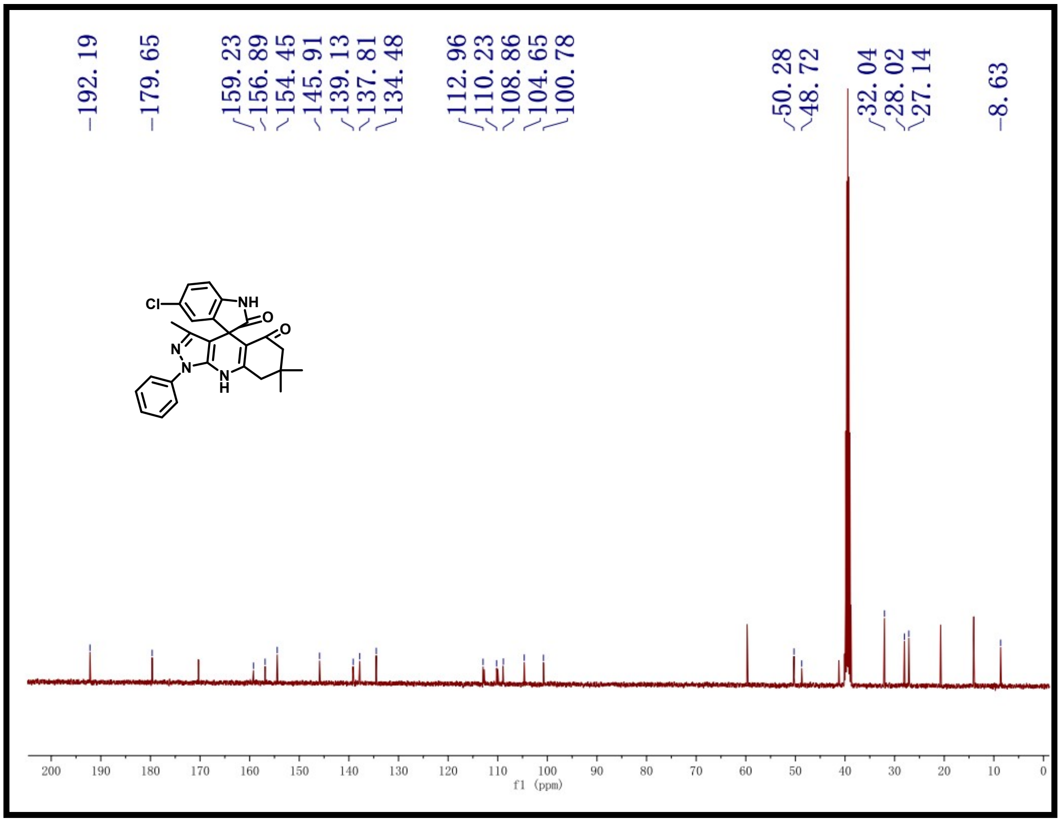

Fig. S14. ^{13}C NMR of (±)-5-Chloro-3',7',7'-trimethyl-1'-phenyl-1',7',8',9'-tetrahydrospiro[indoline-3,4'-pyrazolo[3,4-*b*]quinoline]-2,5'(6'*H*)-dione (1g).

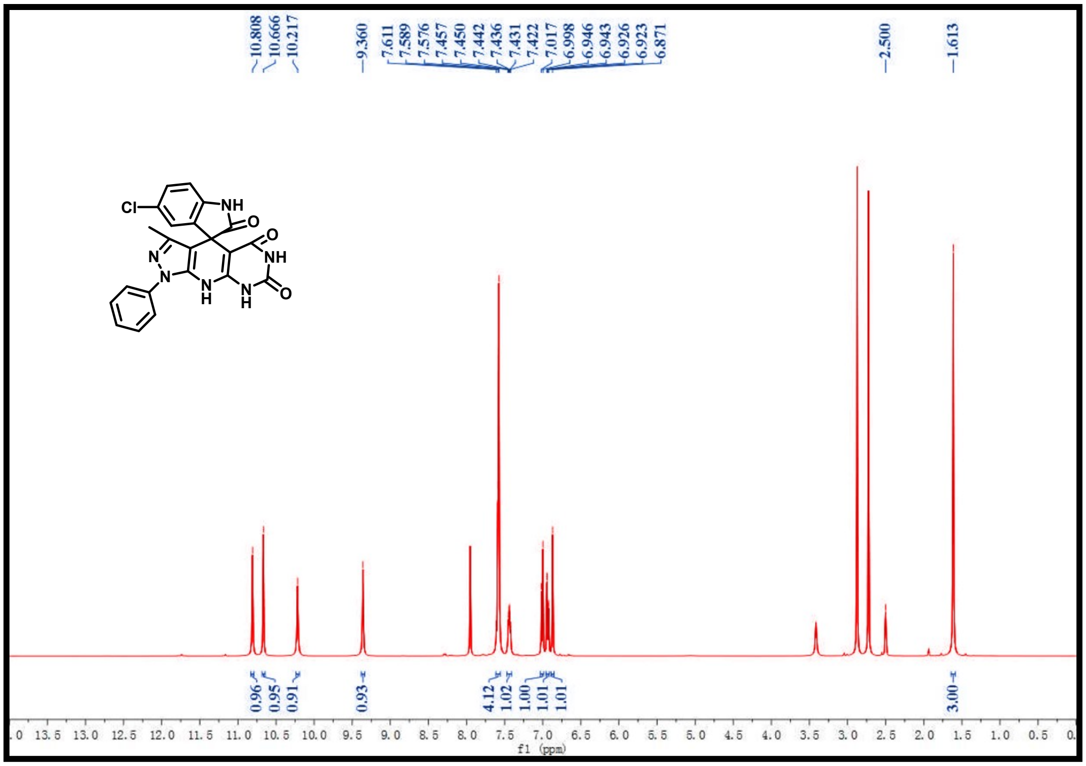

Fig. S15. ^{1}H NMR of (±)-5-Chloro-3′-methyl-1′-phenylspiro[indoline-3,4′-pyrazolo[4′,3′:5,6]pyrido[2,3-*d*]pyrimidine]-2,5′,7′(6′*H*,8′*H*,9′*H*)-trione (1h).

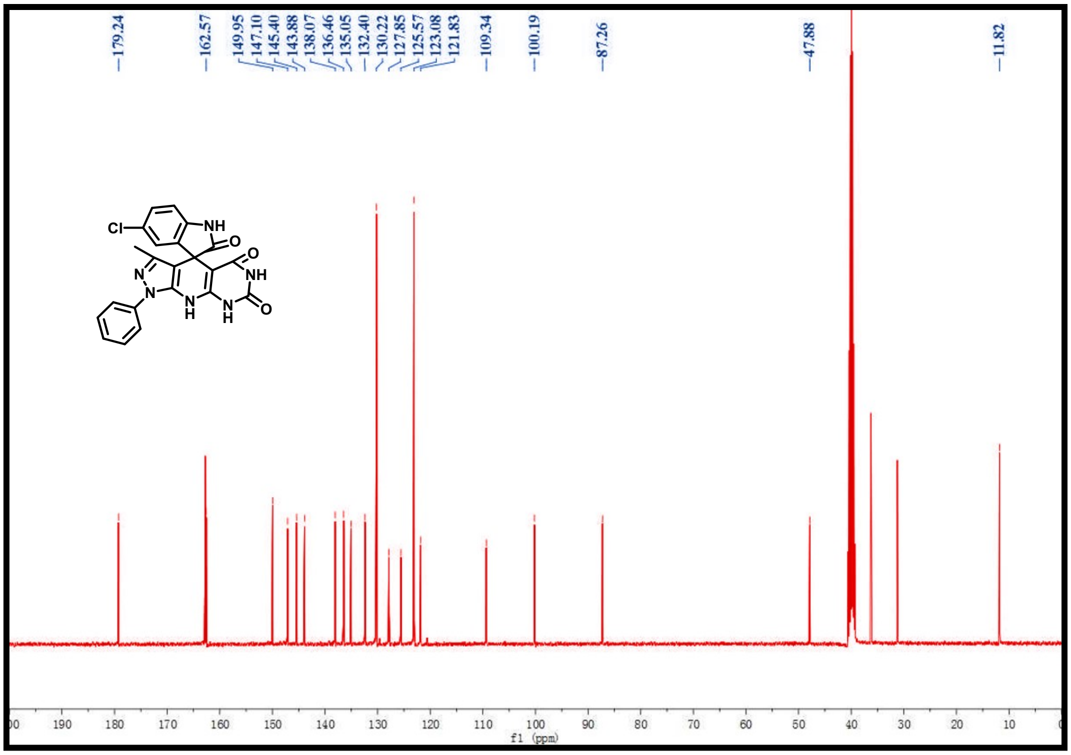

Fig. S16. ^{13}C NMR of (±)-5-Chloro-3′-methyl-1′-phenylspiro[indoline-3,4′-pyrazolo[4′,3′:5,6]pyrido[2,3-*d*]pyrimidine]-2,5′,7′(6′*H*,8′*H*,9′*H*)-trione (1h).

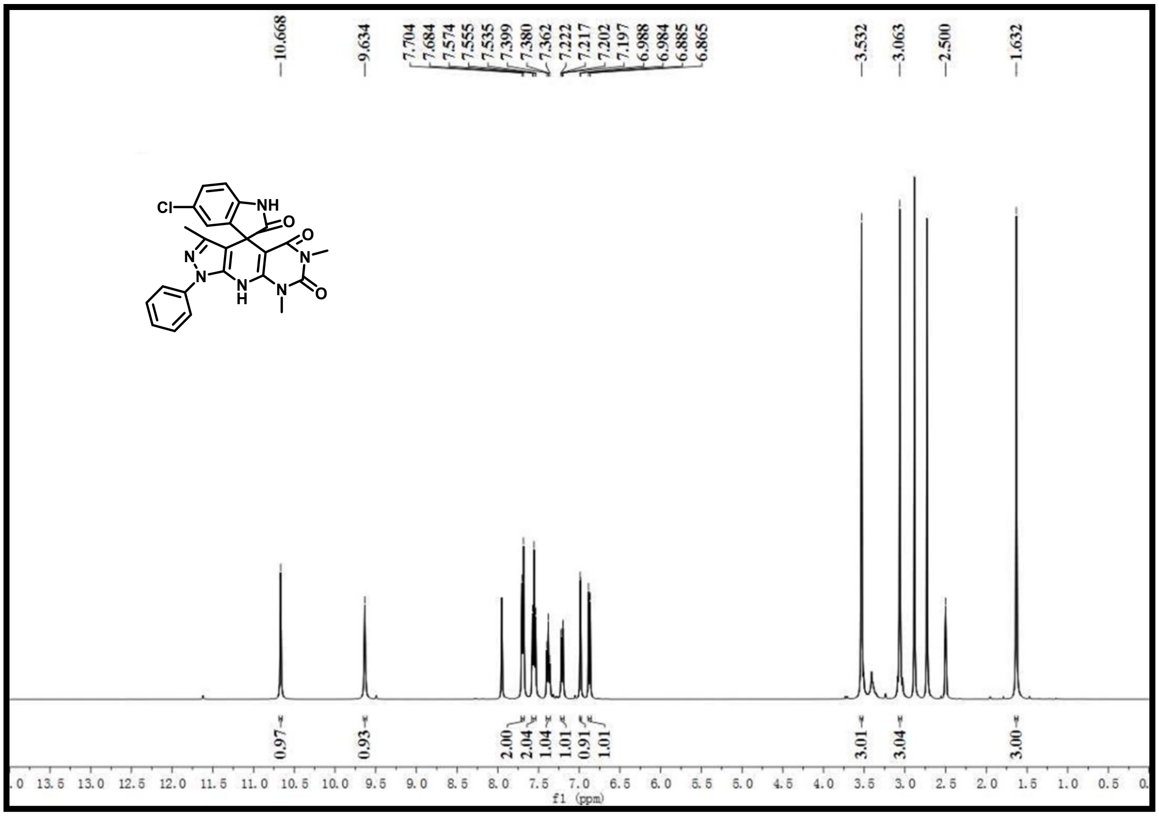

Fig. S17. ^{1}H NMR of (±)-5-Chloro-3′,6′,8′-trimethyl-1′-phenyl-1′,9′-dihydrospiro[indoline-3,4′-pyrazolo[4′,3′:5,6]pyrido[2,3-*d*]pyrimidine]-2,5′,7′(6′*H*,8′*H*)-trione (1i).

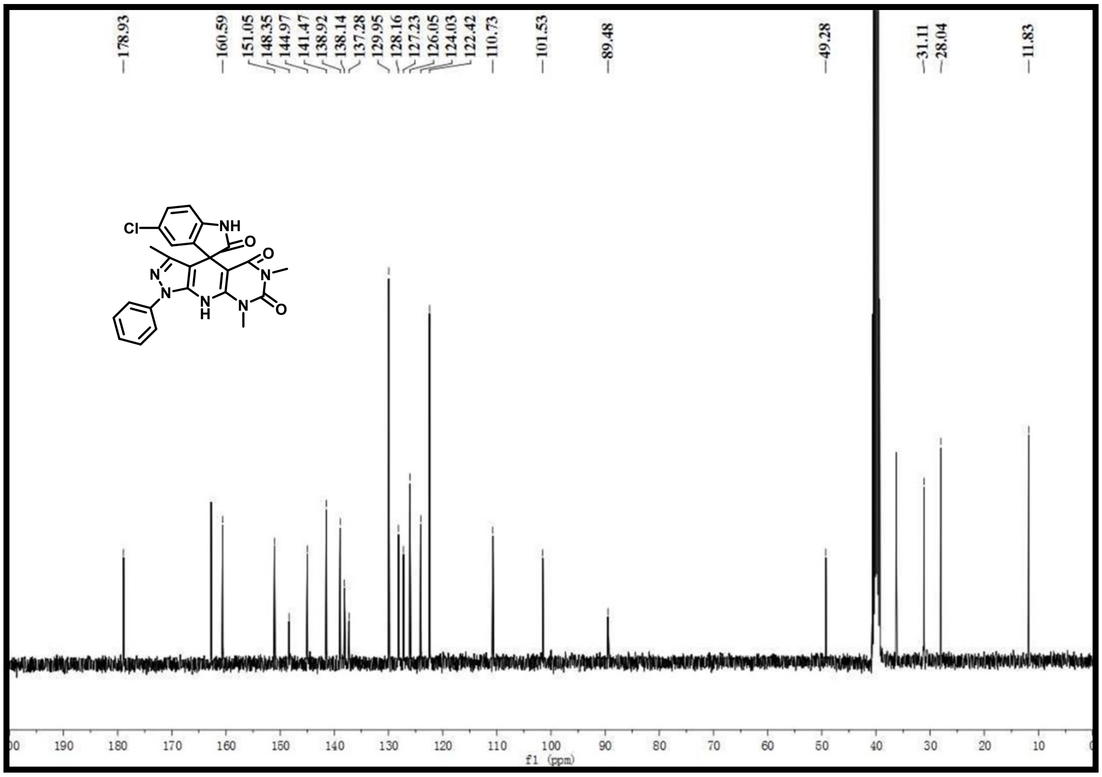

Fig. S18. ^{13}C NMR of (±)-5-Chloro-3′,6′,8′-trimethyl-1′-phenyl-1′,9′-dihydrospiro[indoline-3,4′-pyrazolo[4′,3′:5,6]pyrido[2,3-*d*]pyrimidine]-2,5′,7′(6′*H*,8′*H*)-trione (1i).

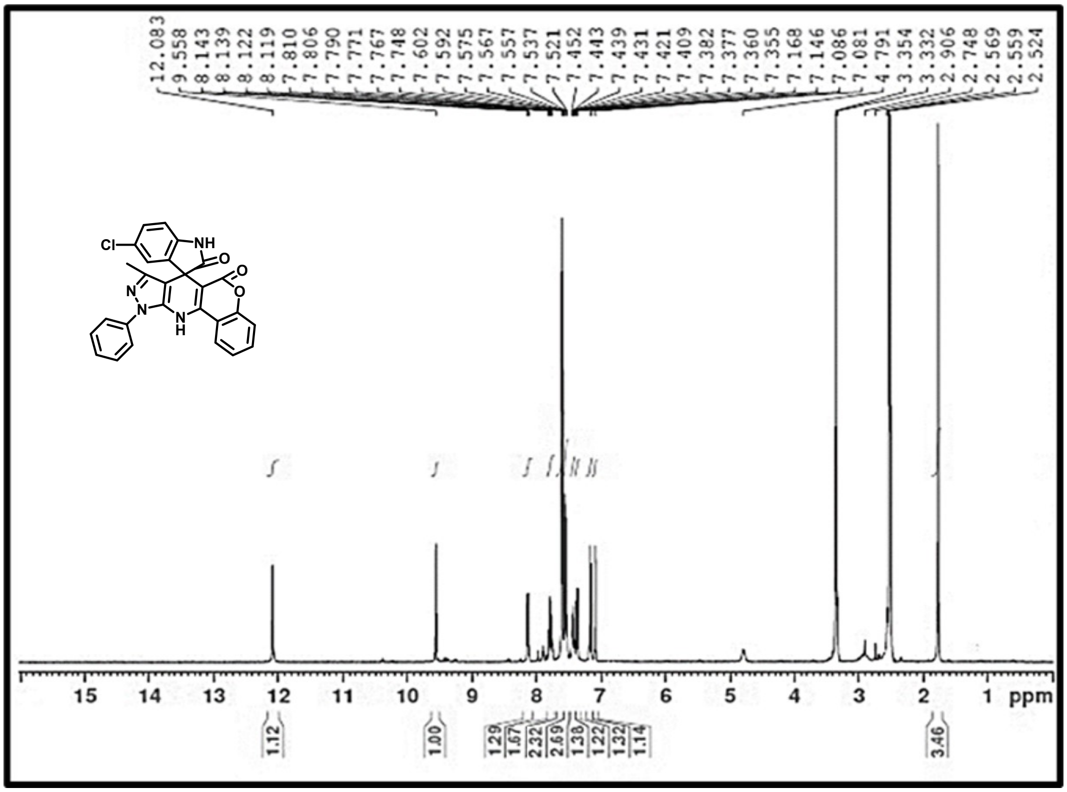

Fig. S19. ^{1}H NMR of (±)-Spiro[1*H*-pyrazolo[5,4-*b*]pyrido[5,6-*c*]chromene-4,3-indoline]-4*H*,5*H*,11*H*-5'-chloro-3-methyl-1-phenyl-5,2'-dione (1j).

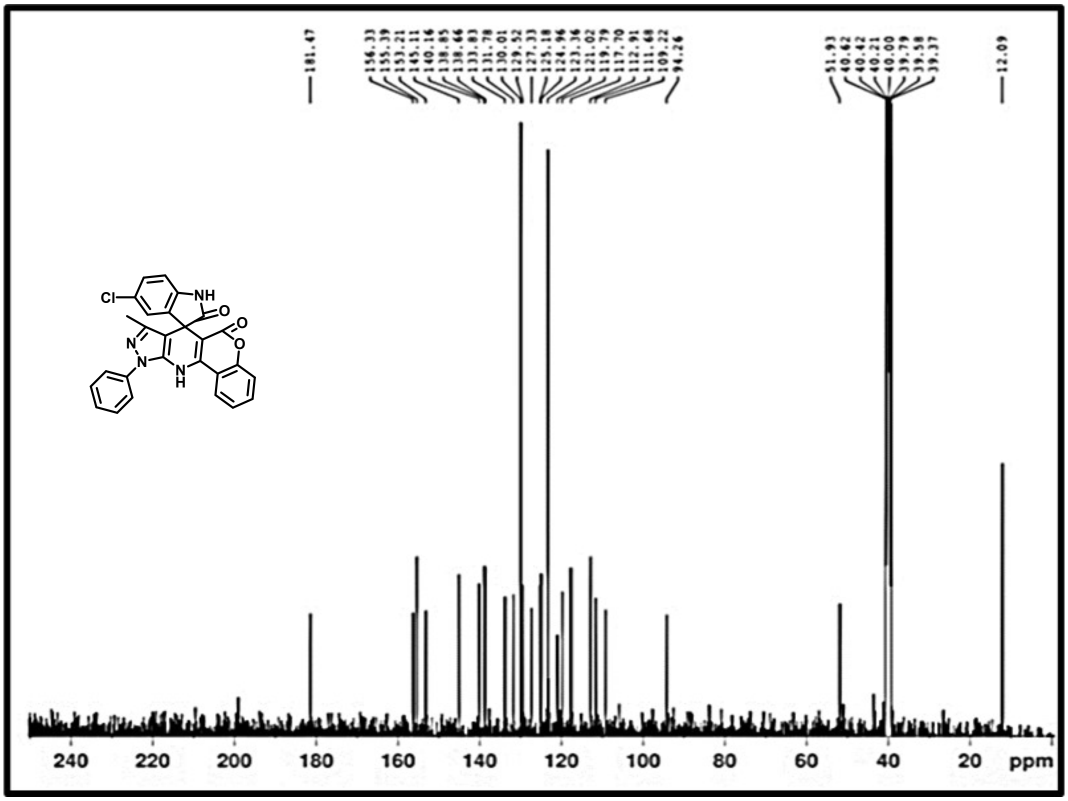

Fig. S20. ^{13}C NMR of (±)-Spiro[1*H*-pyrazolo[5,4-*b*]pyrido[5,6-*c*]chromene-4,3-indoline]-4*H*,5*H*,11*H*-5'-chloro-3-methyl-1-phenyl-5,2'-dione (1j).

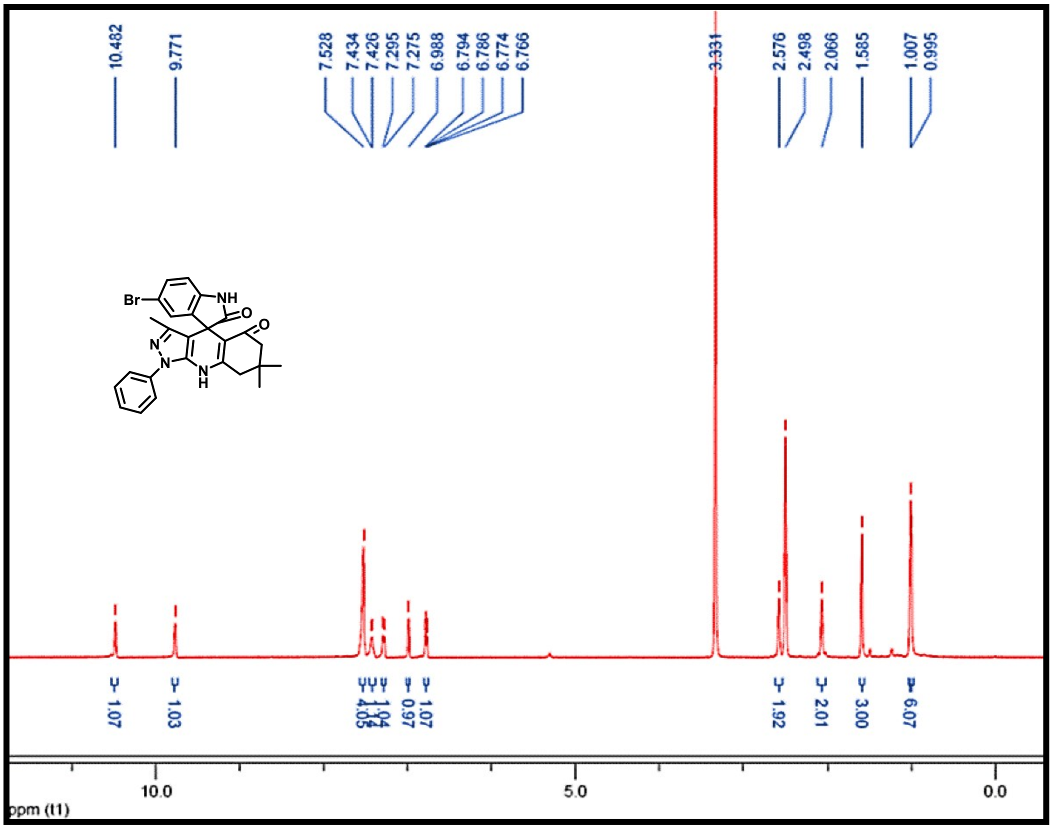

Fig. S21. ^{1}H NMR of (±)-5-Bromo-3',7',7'-trimethyl-1'-phenyl-6',7',8',9'-tetrahydrospiro[indoline-3,4'-pyrazolo[3,4-*b*]quinoline]-2,5'(1'*H*)-dione (1k).

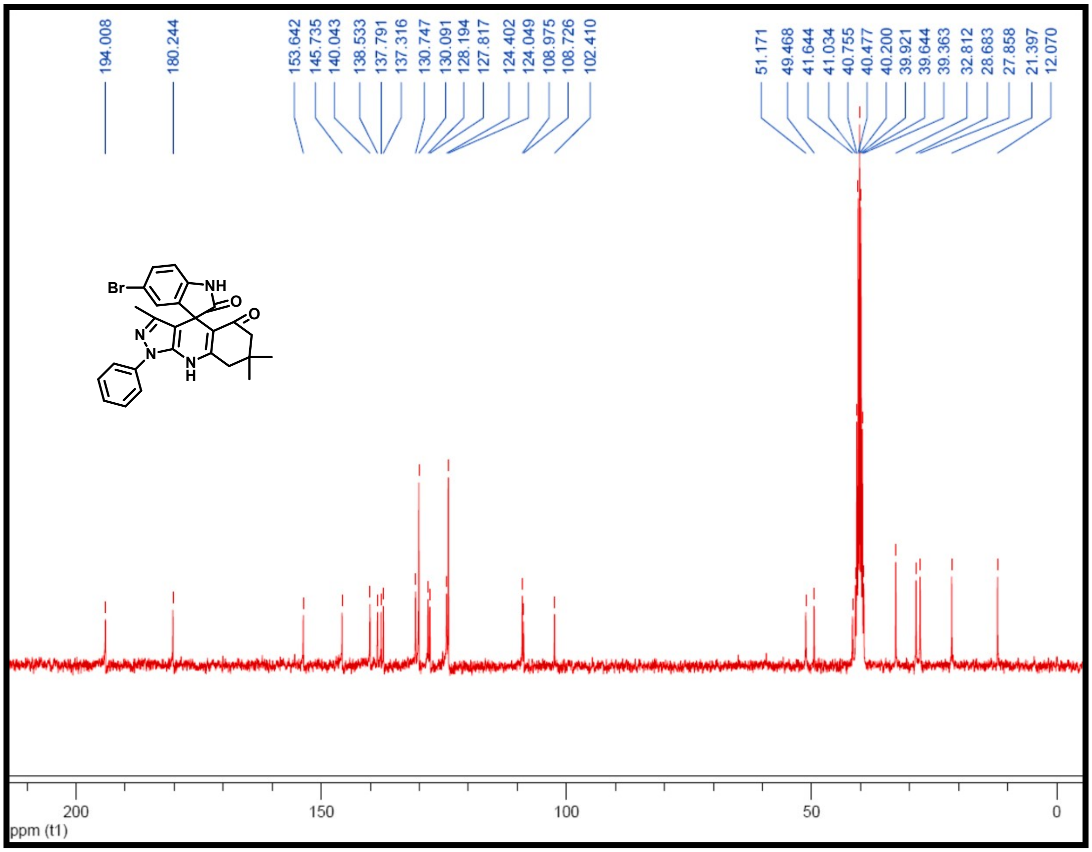

Fig. S22. ^{13}C NMR of (±)-5-Bromo-3',7',7'-trimethyl-1'-phenyl-6',7',8',9'-tetrahydrospiro[indoline-3,4'-pyrazolo[3,4-*b*]quinoline]-2,5'(1'*H*)-dione (1k).

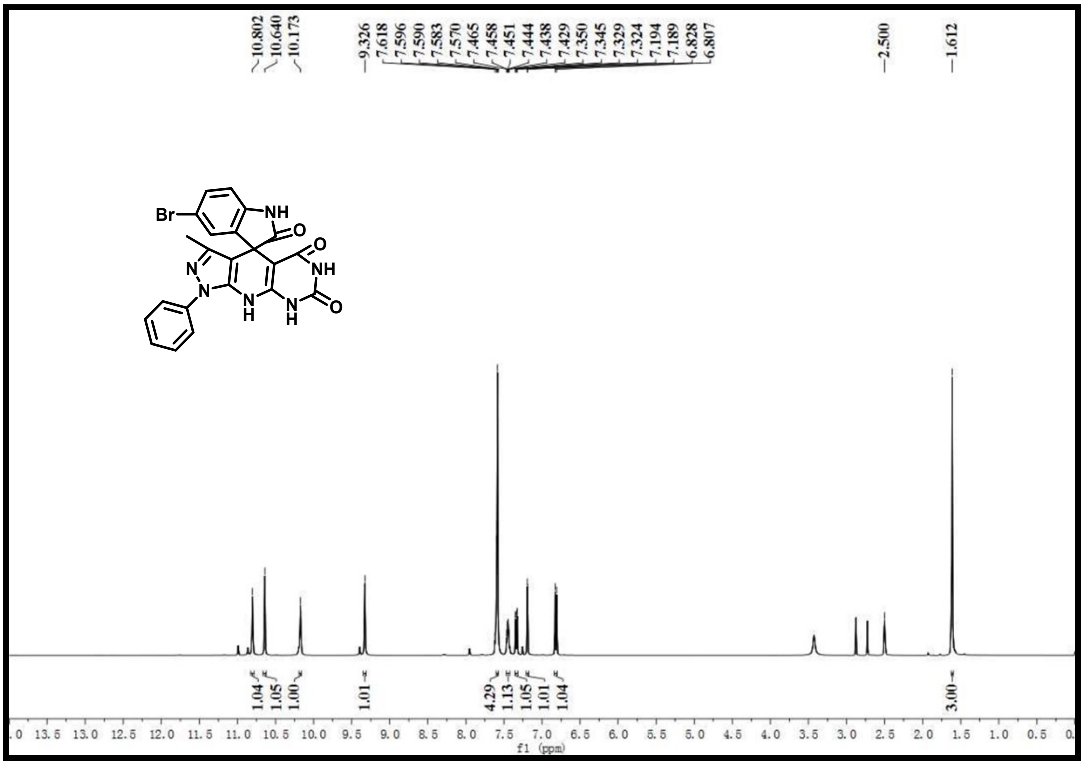

Fig. S23. ^{1}H NMR of (±)-5-Bromo-3′-methyl-1′-phenyl-1′,9′-dihydrospiro[indoline-3,4′-pyrazolo[4′,3′:5,6]pyrido[2,3-*d*]pyrimidine]-2,5′,7′(6′*H*,8′*H*)-trione (1l).

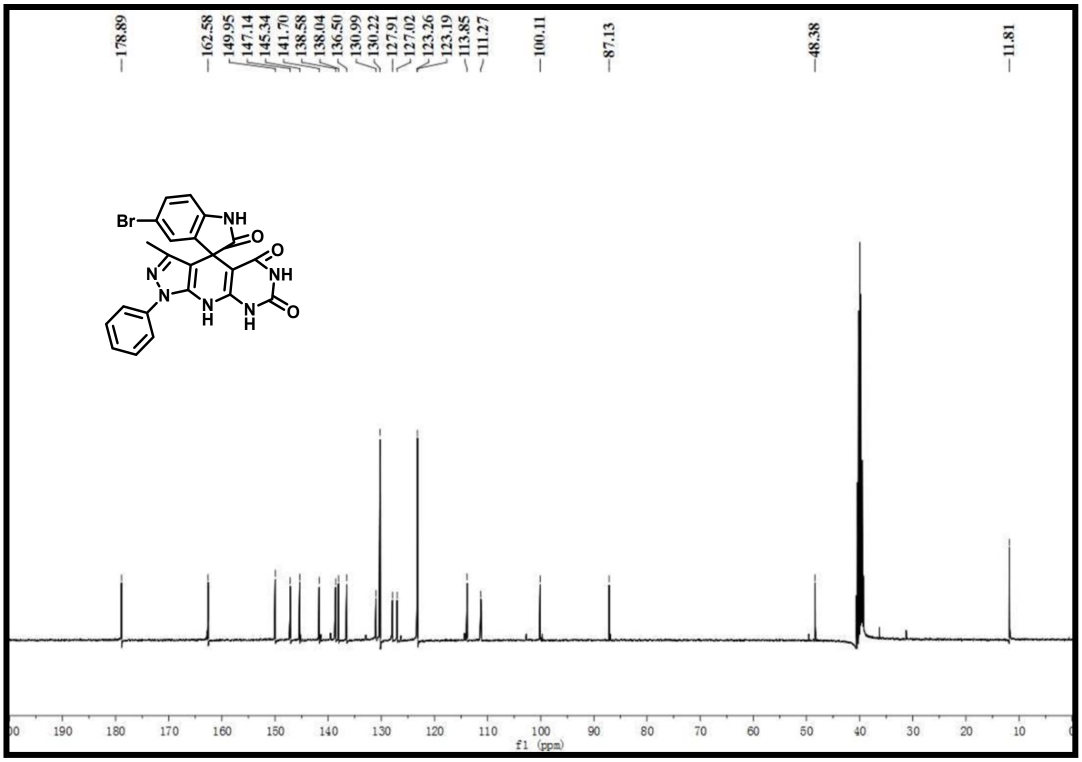

Fig. S24. ^{13}C NMR of (±)-5-Bromo-3′-methyl-1′-phenyl-1′,9′-dihydrospiro[indoline-3,4′-pyrazolo[4′,3′:5,6]pyrido[2,3-*d*]pyrimidine]-2,5′,7′(6′*H*,8′*H*)-trione (1l).

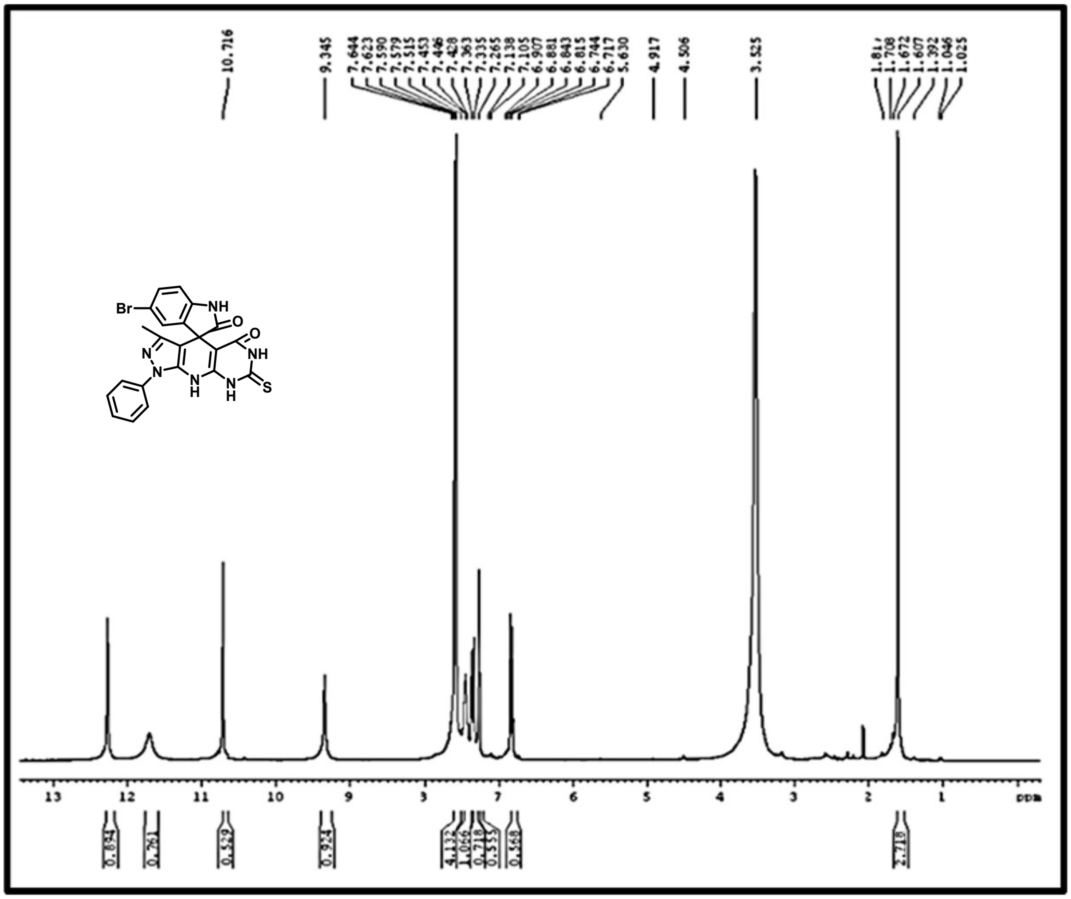

Fig. S25. ^{1}H NMR of (±)-5-Bromo-3′-methyl-1′-phenyl-7′-thioxo-spiro-[indoline-3,4′-pyrazolo[4′,3′:5,6]pyrido[2,3-*d*]pyrimidine]-2,5′(6′*H*,8′*H*,9′*H*)-dione (1m).

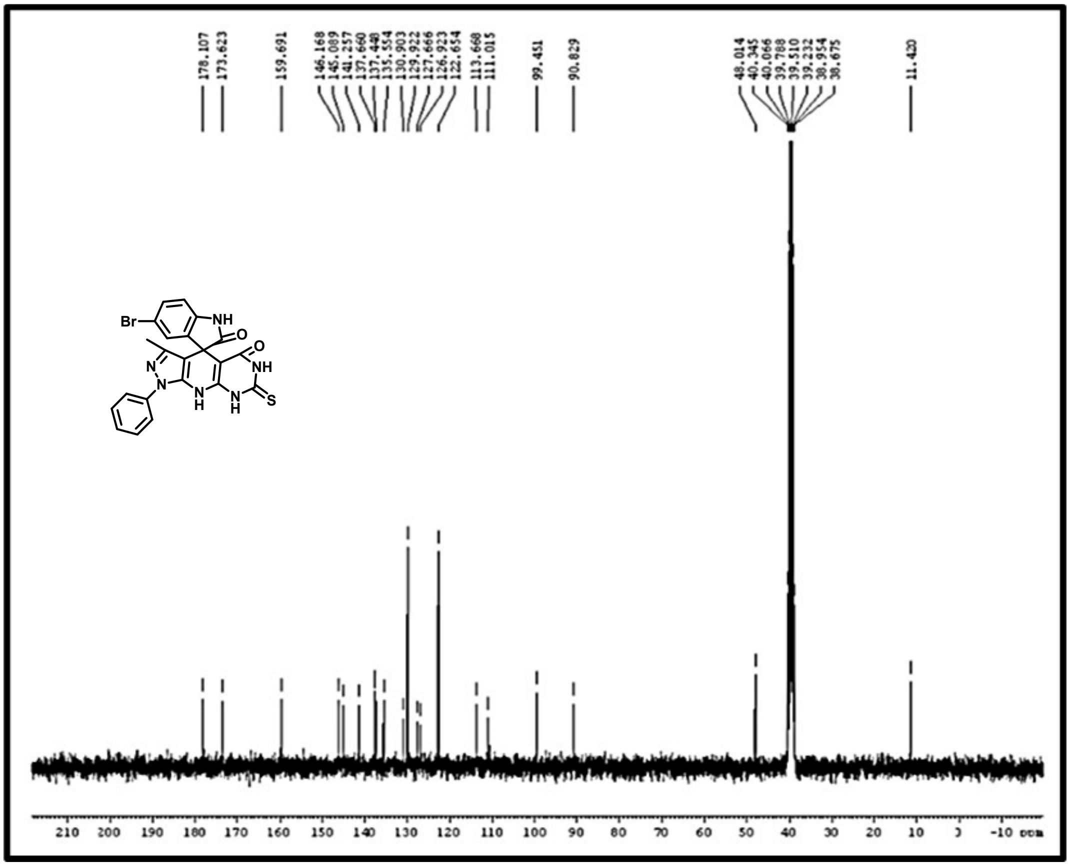

Fig. S26. ^{13}C NMR of (±)-5-Bromo-3′-methyl-1′-phenyl-7′-thioxo-spiro-[indoline-3,4′-pyrazolo[4′,3′:5,6]pyrido[2,3-*d*]pyrimidine]-2,5′(6′*H*,8′*H*,9′*H*)-dione (1m).

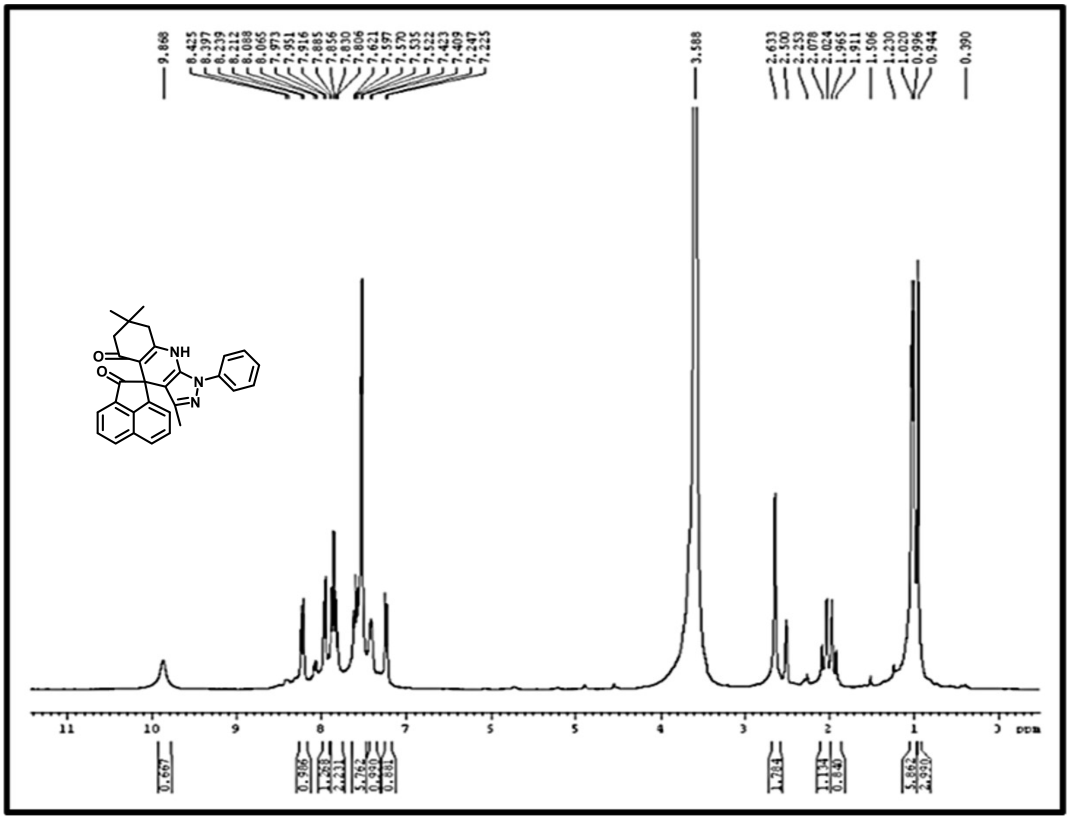

Fig. S27. ^{1}H NMR of (±)-3′,7′,7′-Trimethyl-1′-phenyl-6′,7′,8′,9′-tetra-hydro-2*H*-spiro[acenaphthylene-1,4′-pyrazolo[3,4-*b*]quinoline]-2,5′(1′*H*)-dione (1n).

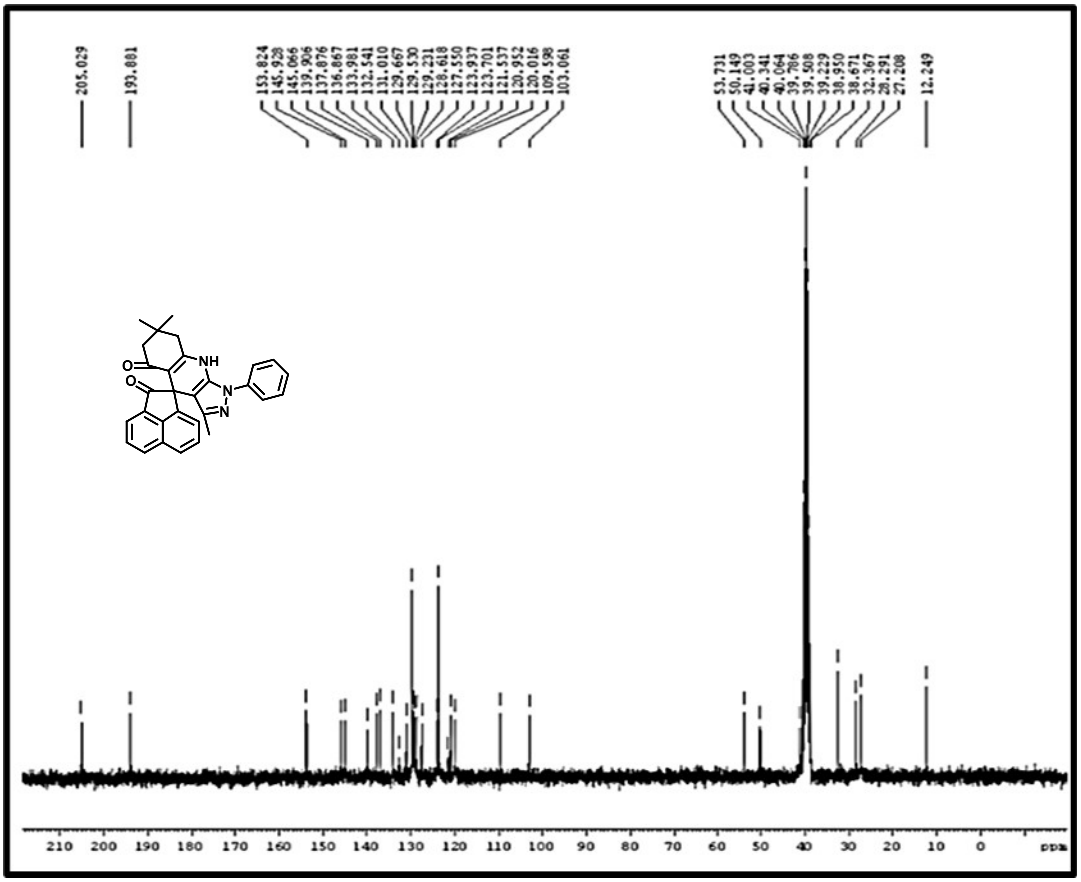

Fig. S28. ^{13}C NMR of (±)-3′,7′,7′-Trimethyl-1′-phenyl-6′,7′,8′,9′-tetra-hydro-2*H*-spiro[acenaphthylene-1,4′-pyrazolo[3,4-*b*]quinoline]-2,5′(1′*H*)-dione (1n).

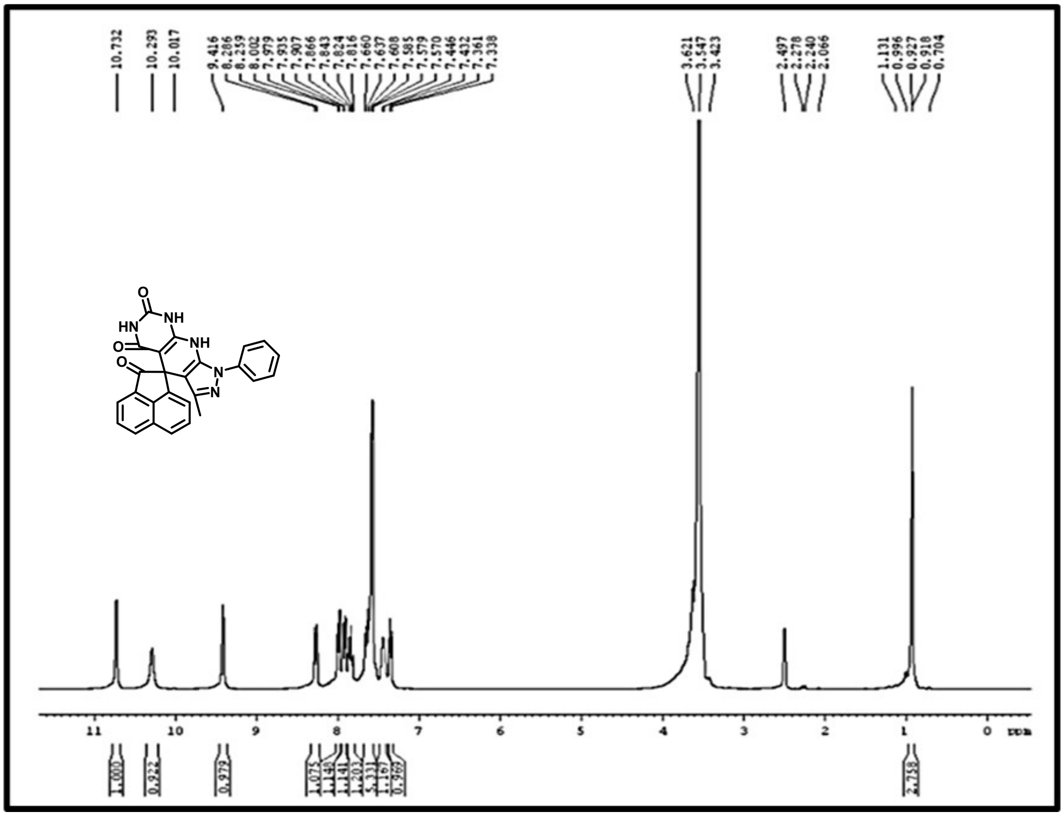

Fig. S29. ^{1}H NMR of (±)-3′-Methyl-1′-phenyl-spiro[acenaphthylene-3,4′-pyrazolo[4′,3′:5,6]pyrido[2,3-*d*]pyrimidine]-2,5′,7′(6′*H*,8′*H*,9′*H*)-trione (1o).

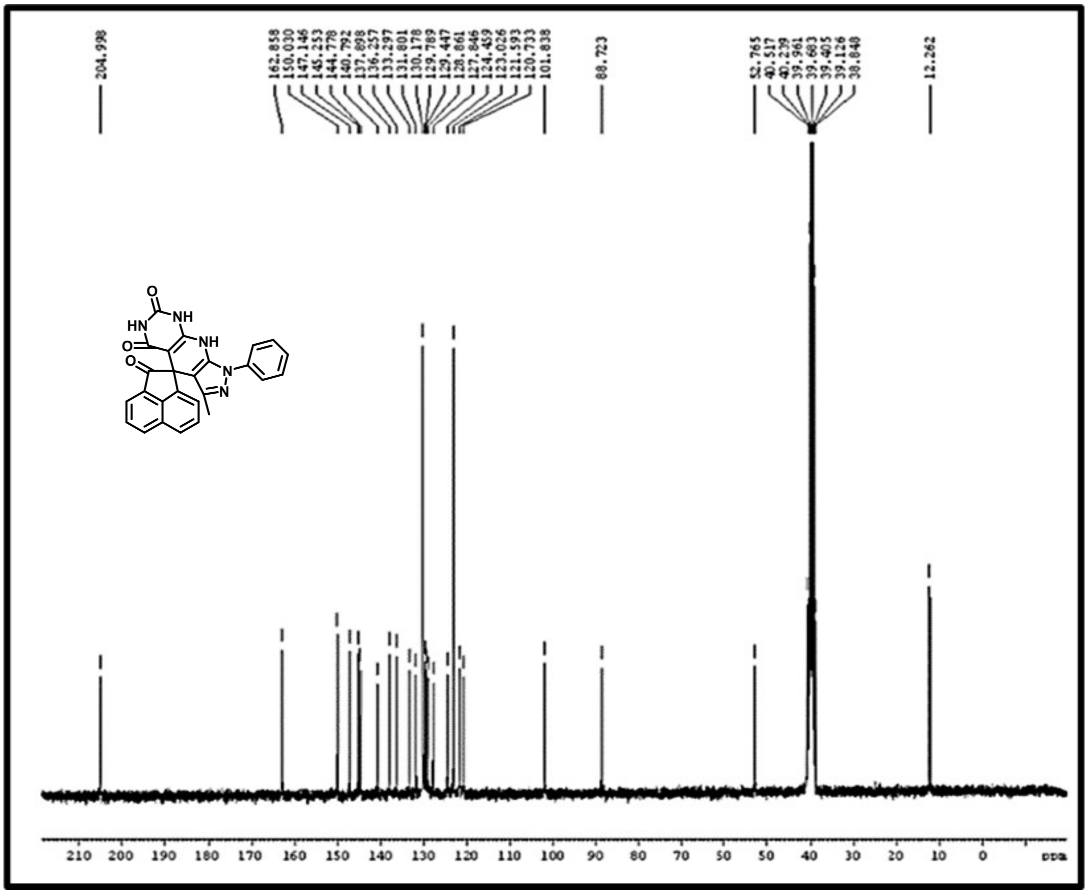

Fig. S30. ^{13}C NMR of (±)-3′-Methyl-1′-phenyl-spiro[acenaphthylene-3,4′-pyrazolo[4′,3′:5,6]pyrido[2,3-*d*]pyrimidine]-2,5′,7′(6′*H*,8′*H*,9′*H*)-trione (1o).

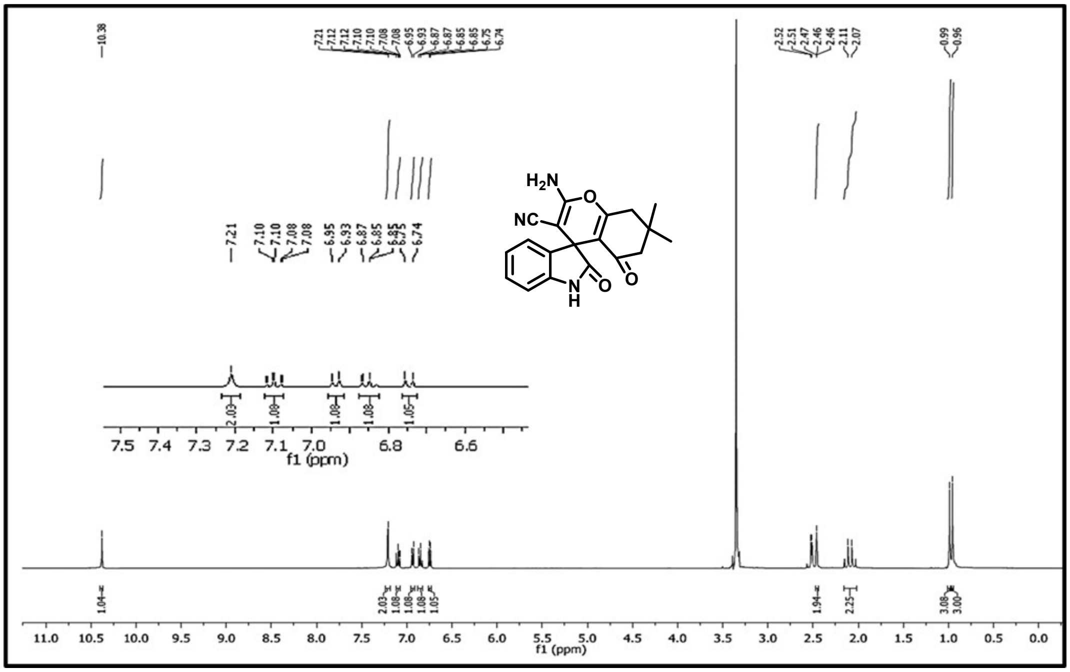

Fig. S31. ^{1}H NMR of (±)-2-Amino-7,7-dimethyl-2′,5-dioxo-5,6,7,8-tetrahydrospiro[chromene-4,3′-indoline]-3-carbonitrile (2a).

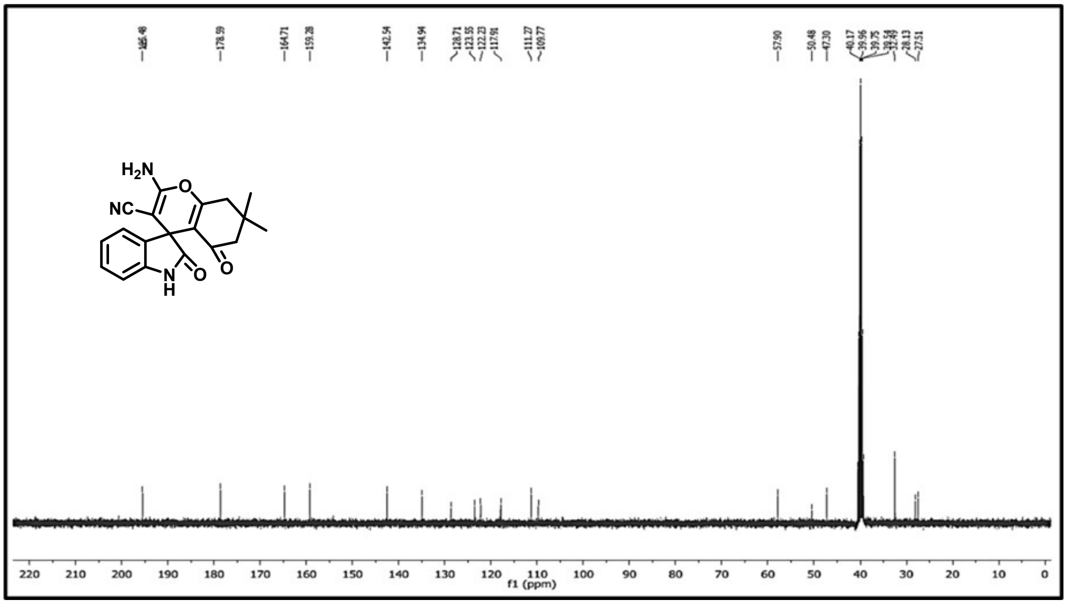

Fig. S32. ^{13}C NMR of (±)-2-Amino-7,7-dimethyl-2′,5-dioxo-5,6,7,8-tetrahydrospiro[chromene-4,3′-indoline]-3-carbonitrile (2a).

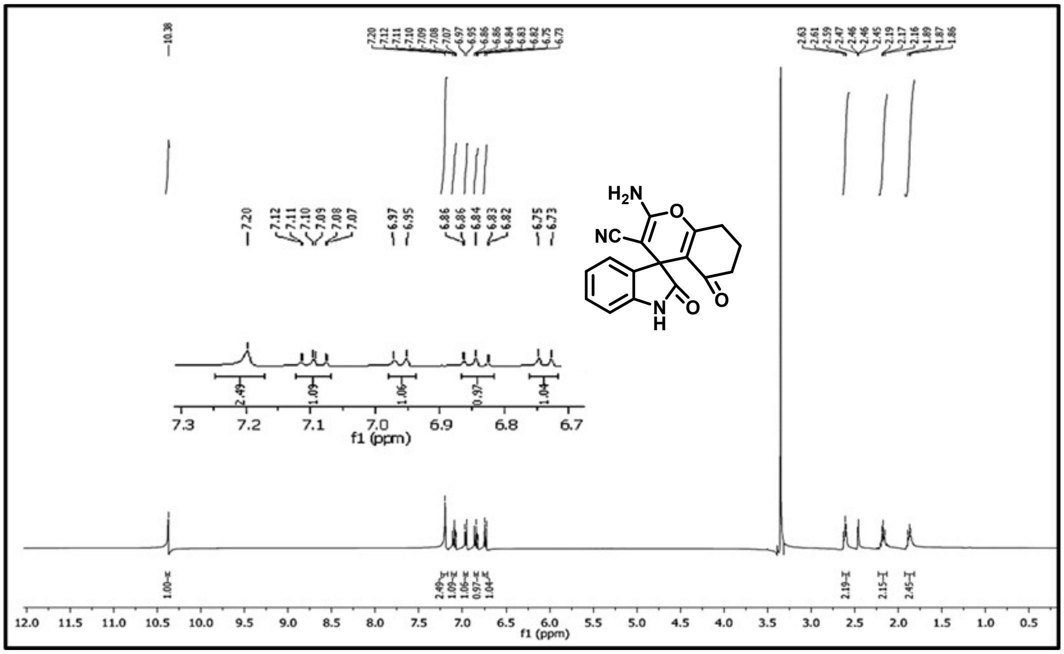

Fig. S33. ^1H NMR of (±)-2-Amino-2',5-dioxo-5,6,7,8-tetrahydrospiro[chromene-4,3'-indoline]-3-carbonitrile (2b).

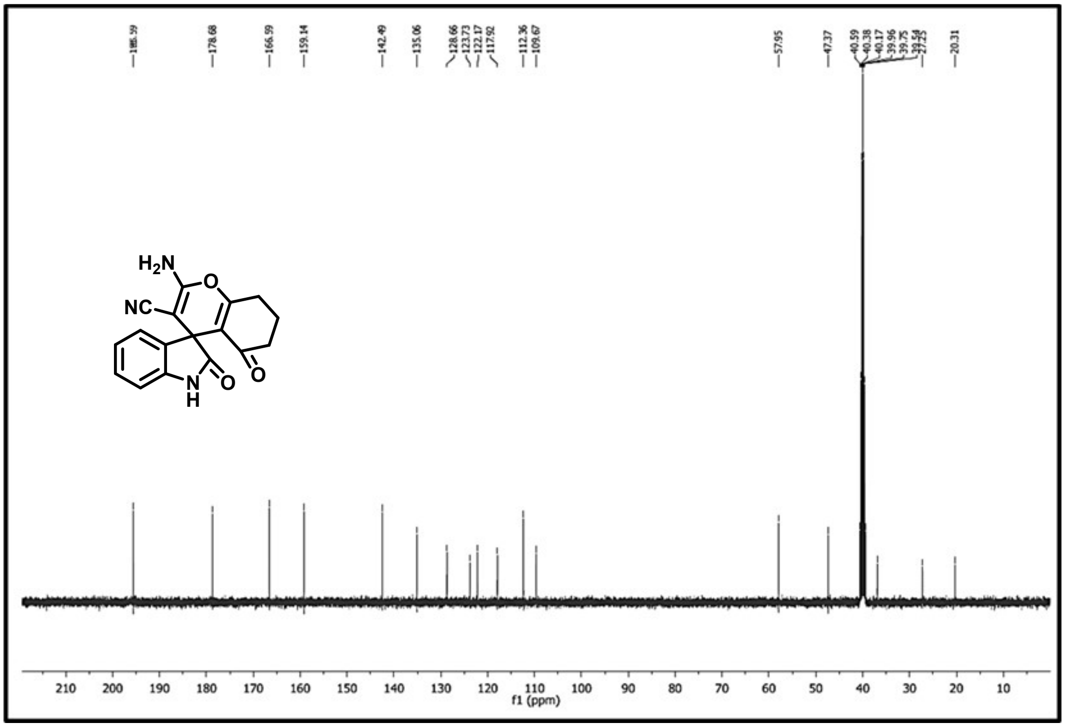

Fig. S34. ^{13}C NMR of (±)-2-Amino-2',5-dioxo-5,6,7,8-tetrahydrospiro[chromene-4,3'-indoline]-3-carbonitrile (2b).

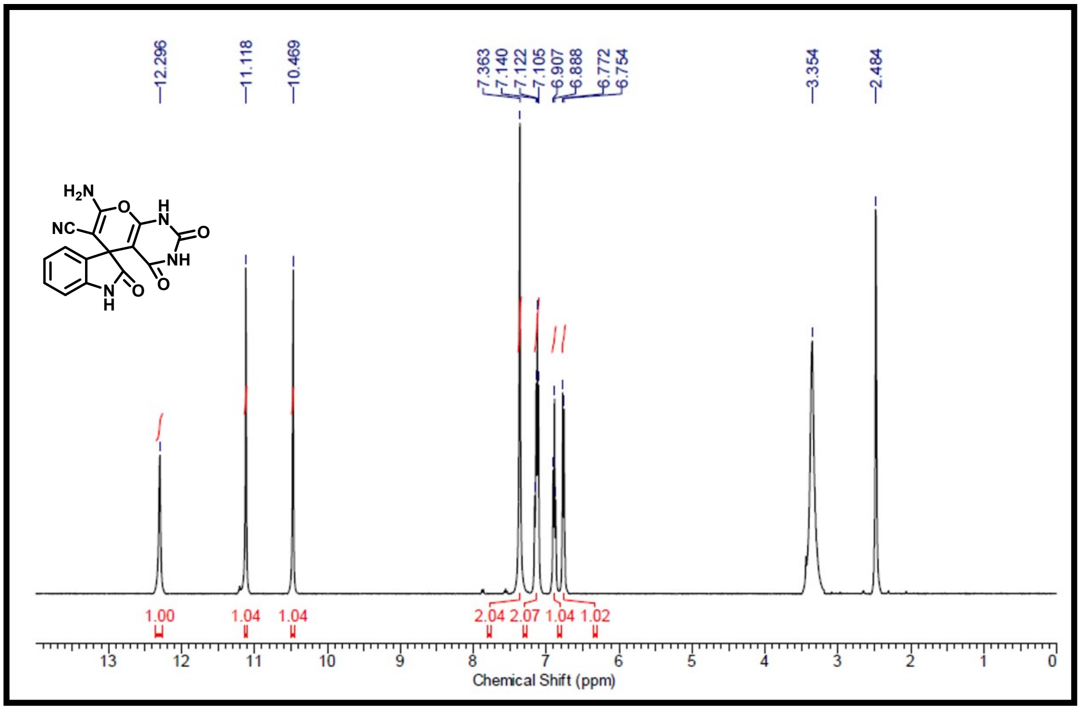

Fig. S35. ^{1}H NMR of (±)-7′-Amino-2,2′,4′-trioxo-1′,2′,3′,4′-tetrahydrospiro[indoline-3,5′-pyrano[2,3-*d*]pyrimidine]-6′-carbonitrile (2c).

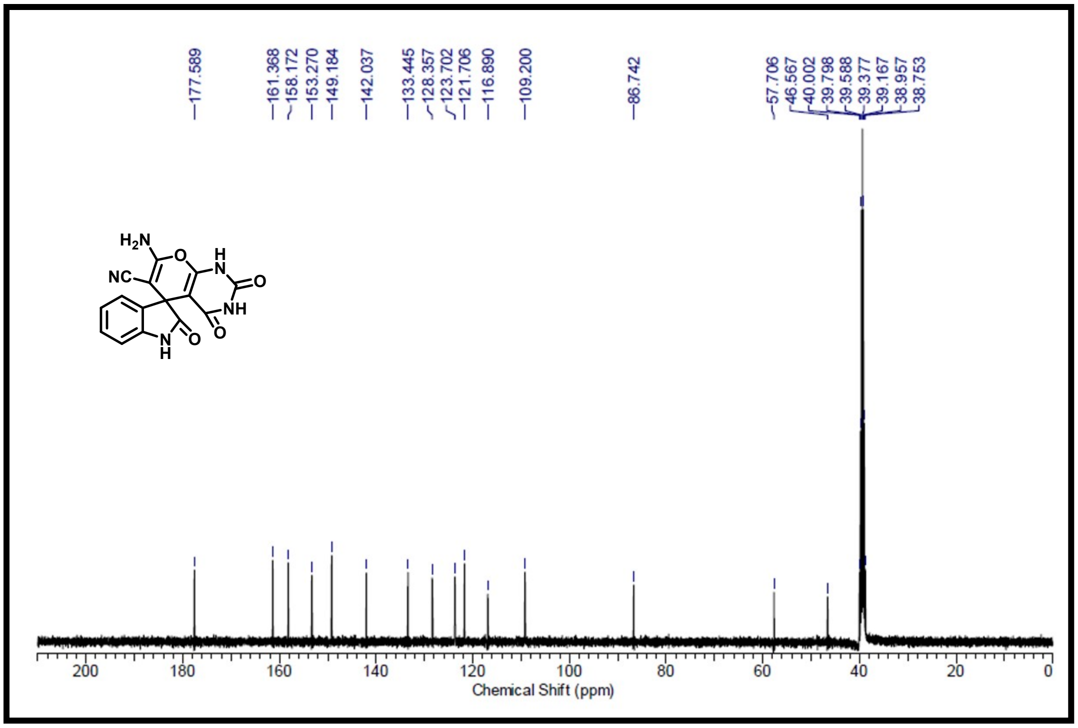

Fig. S36. ^{13}C NMR of (±)-7′-Amino-2,2′,4′-trioxo-1′,2′,3′,4′-tetrahydrospiro[indoline-3,5′-pyrano[2,3-*d*]pyrimidine]-6′-carbonitrile (2c).

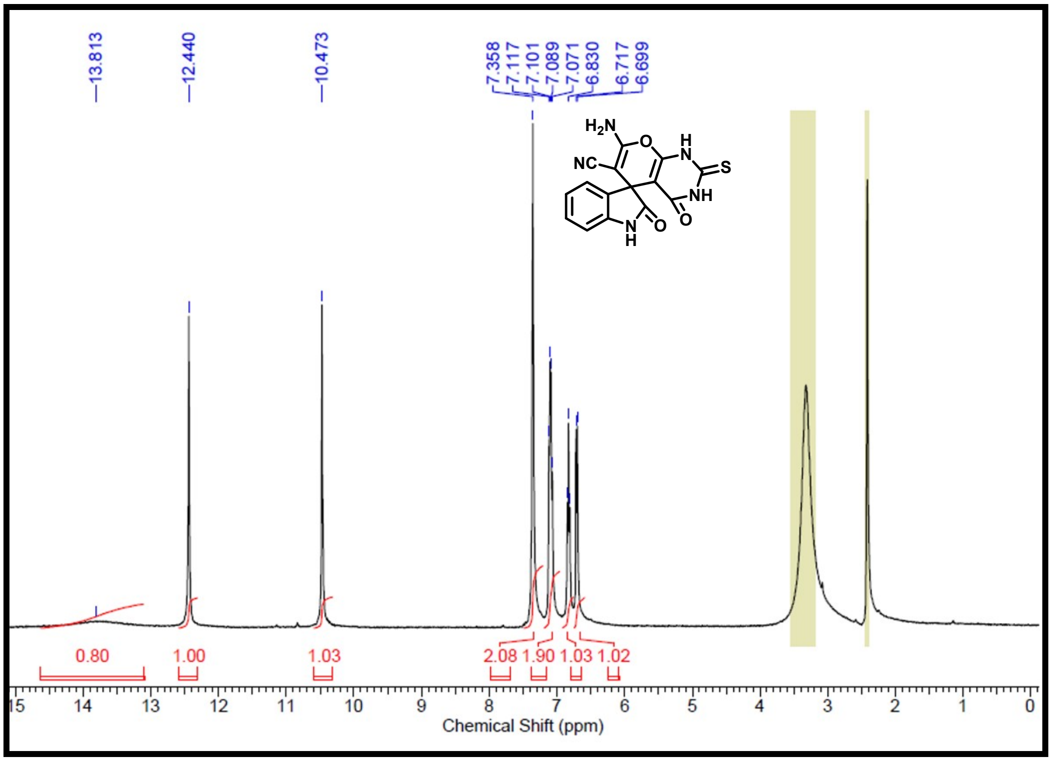

Fig. S37. ^{1}H NMR of (±)-7'-Amino-2,4'-dioxo-2'-thioxo-1',2',3',4'-tetrahydrospiro[indoline-3,5'-pyrano[2,3-*d*]pyrimidine]-6'-carbonitrile (2d).

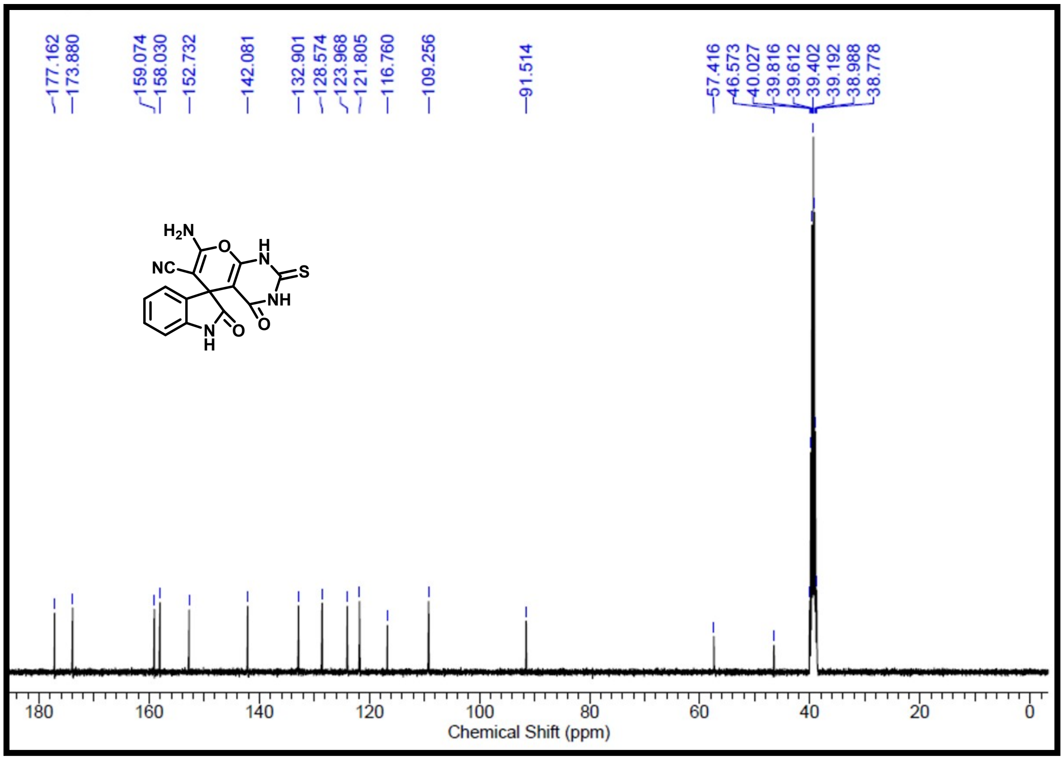

Fig. S38. ^{13}C NMR of (±)-7'-Amino-2,4'-dioxo-2'-thioxo-1',2',3',4'-tetrahydrospiro[indoline-3,5'-pyrano[2,3-*d*]pyrimidine]-6'-carbonitrile (2d).

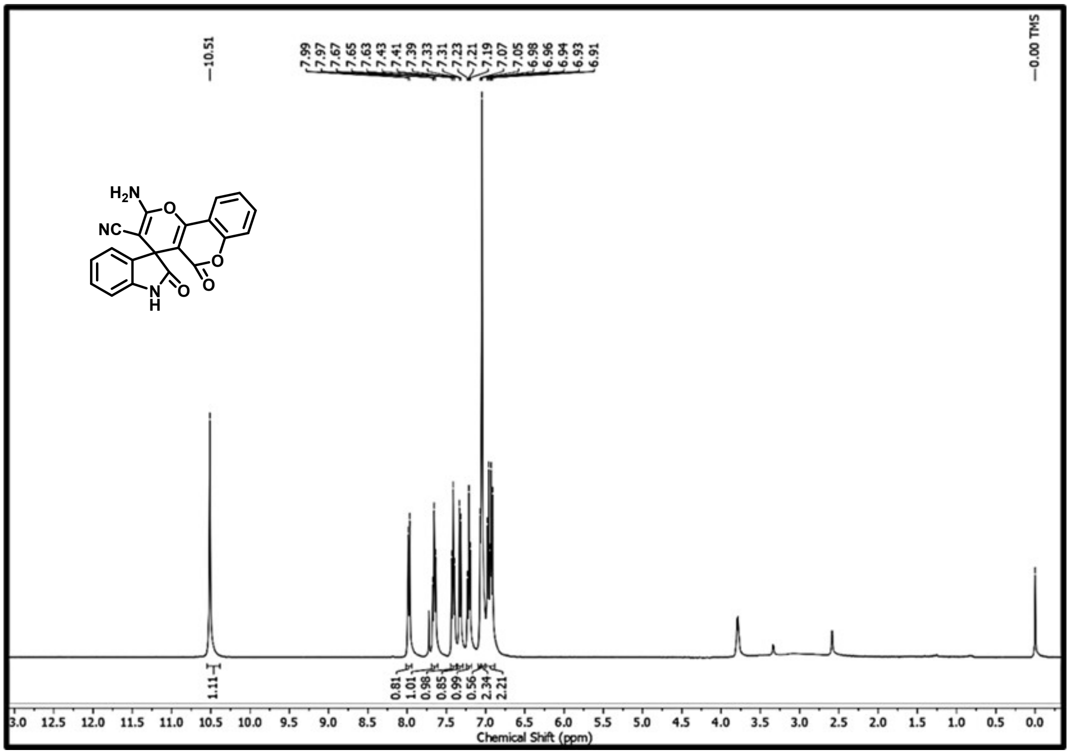

Fig. S39. ^{1}H NMR of (±)-2'-Amino-2,5'-dioxo-5'*H*-spiro[indoline-3,4'-pyrano[3,2-*c*]chromene]-3'-carbonitrile (2e).

.

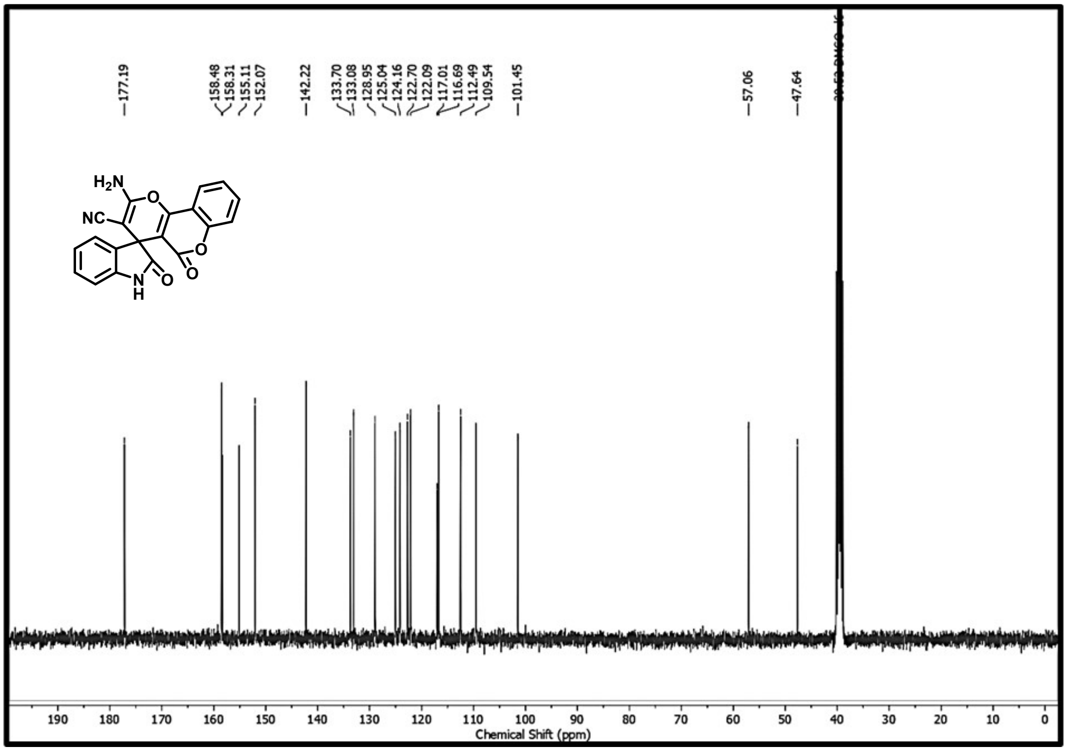

Fig. S40. ^{13}C NMR of (±)-2'-Amino-2,5'-dioxo-5'*H*-spiro[indoline-3,4'-pyrano[3,2-*c*]chromene]-3'-carbonitrile (2e).

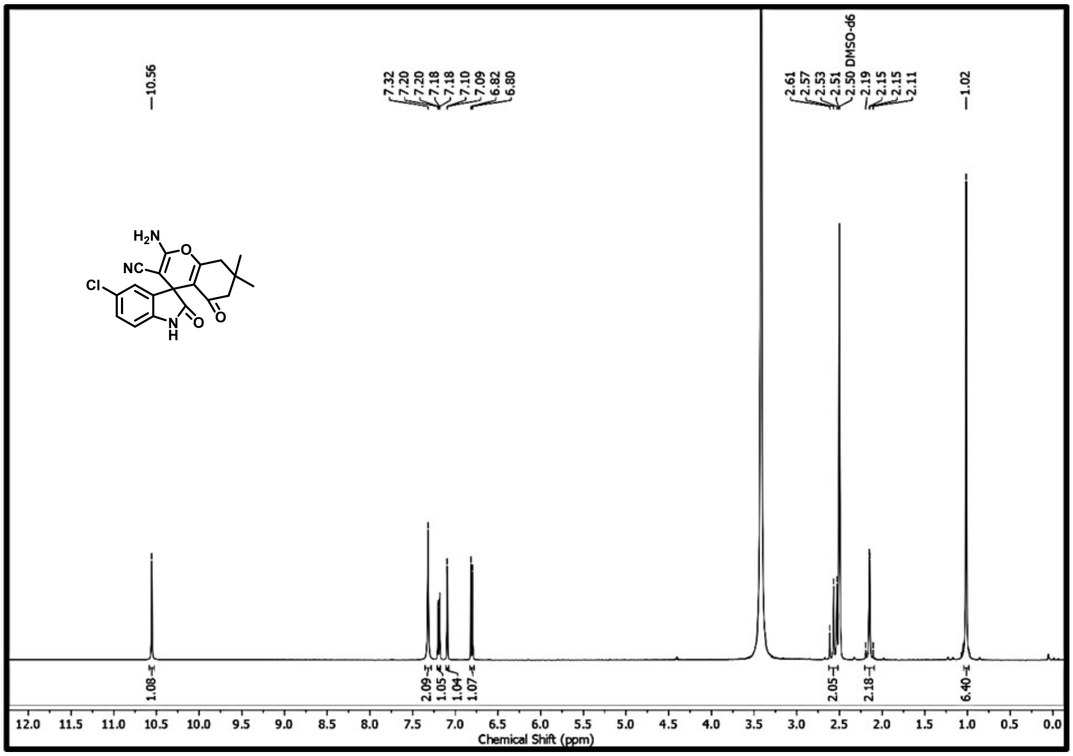

Fig. S41. ^{1}H NMR of (±)-2-Amino-5'-chloro-7,7-dimethyl-2',5-dioxo-5,6,7,8-tetrahydrospiro[chromene-4,3'-indoline]-3-carbonitrile (2f).

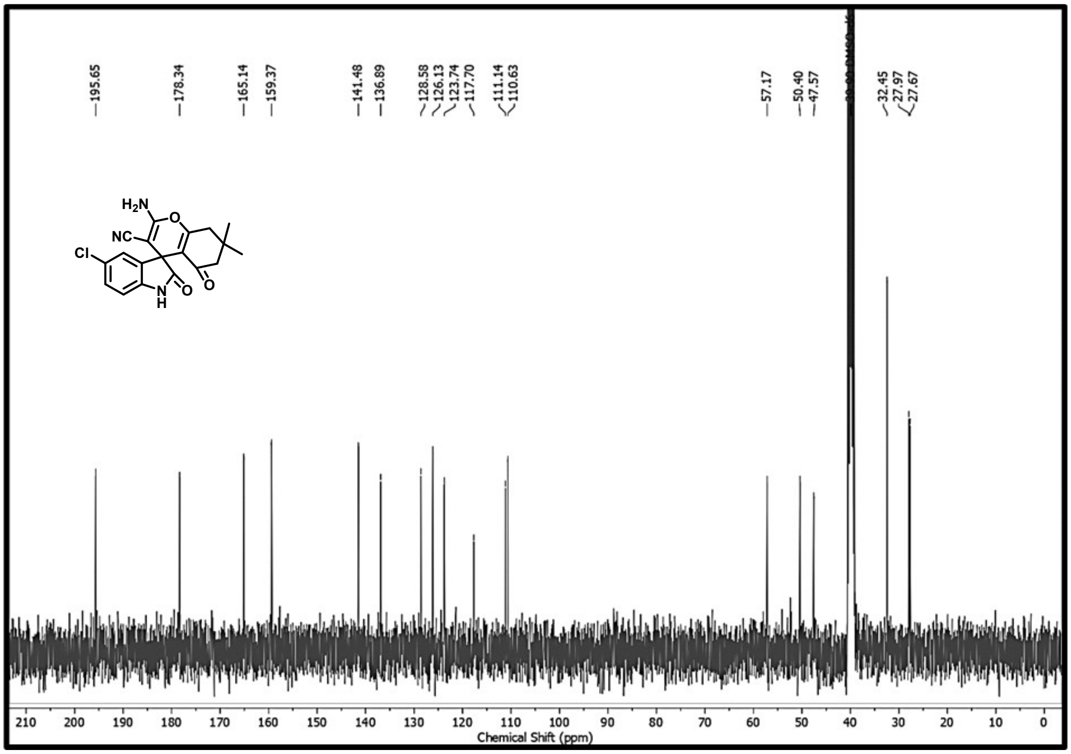

Fig. S42. ^{13}C NMR of (±)-2-Amino-5'-chloro-7,7-dimethyl-2',5-dioxo-5,6,7,8-tetrahydrospiro[chromene-4,3'-indoline]-3-carbonitrile (2f).

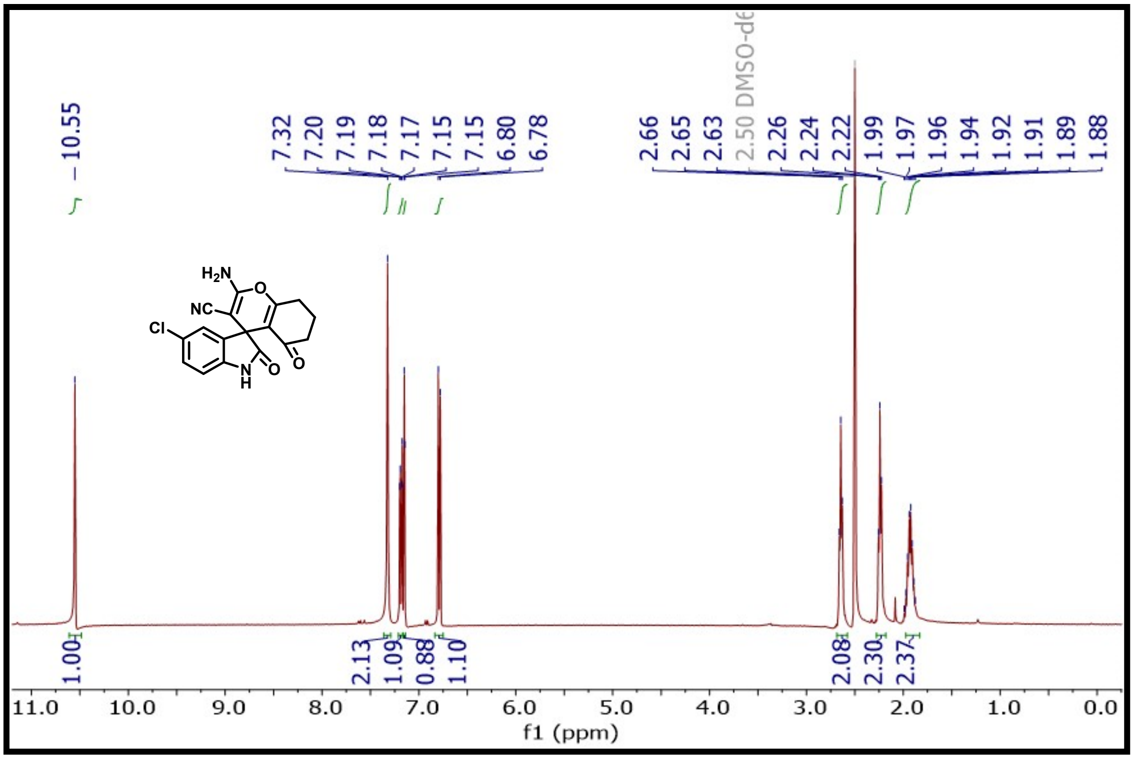

Fig. S43. ^{1}H NMR of (±)-2-Amino-5'-chloro-2',5-dioxo-5,6,7,8-tetrahydrospiro[chromene-4,3'-indoline]-3-carbonitrile (2g).

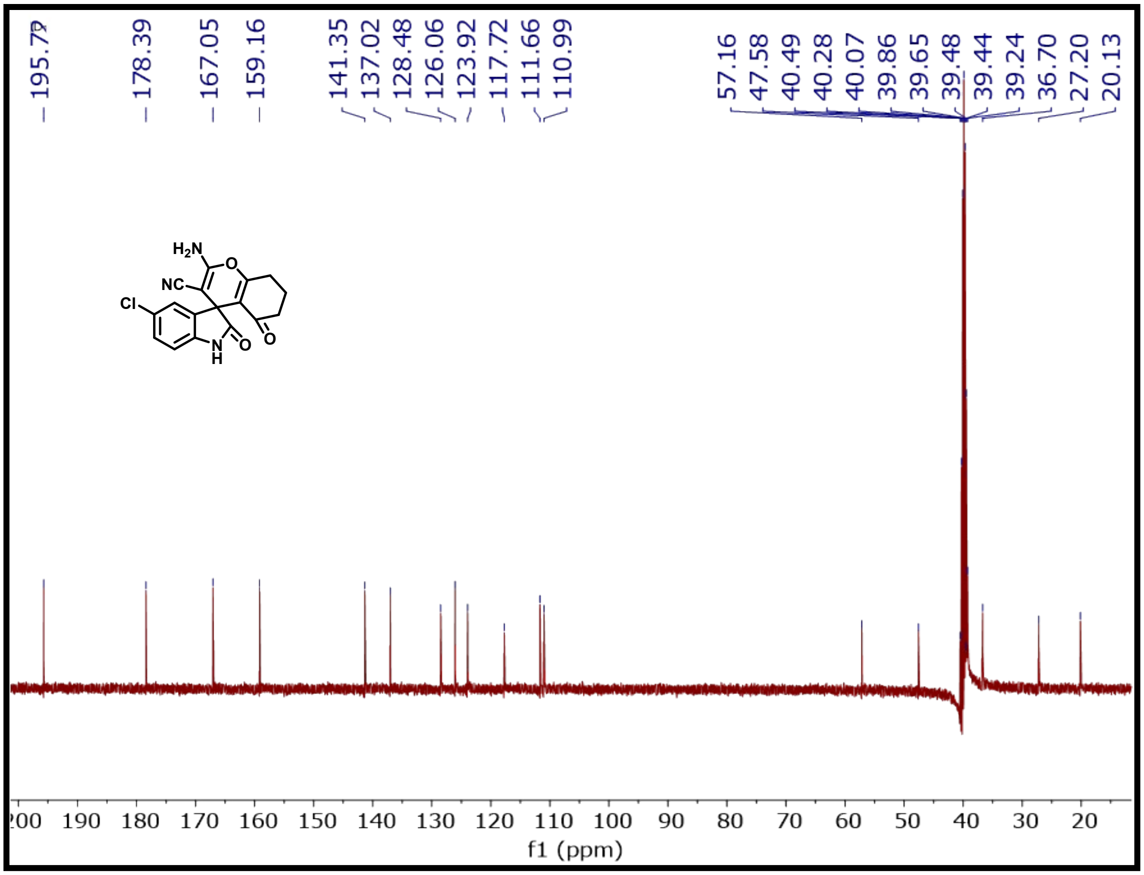

Fig. S44. ^{13}C NMR of (±)-2-Amino-5'-chloro-2',5-dioxo-5,6,7,8-tetrahydrospiro[chromene-4,3'-indoline]-3-carbonitrile (2g).

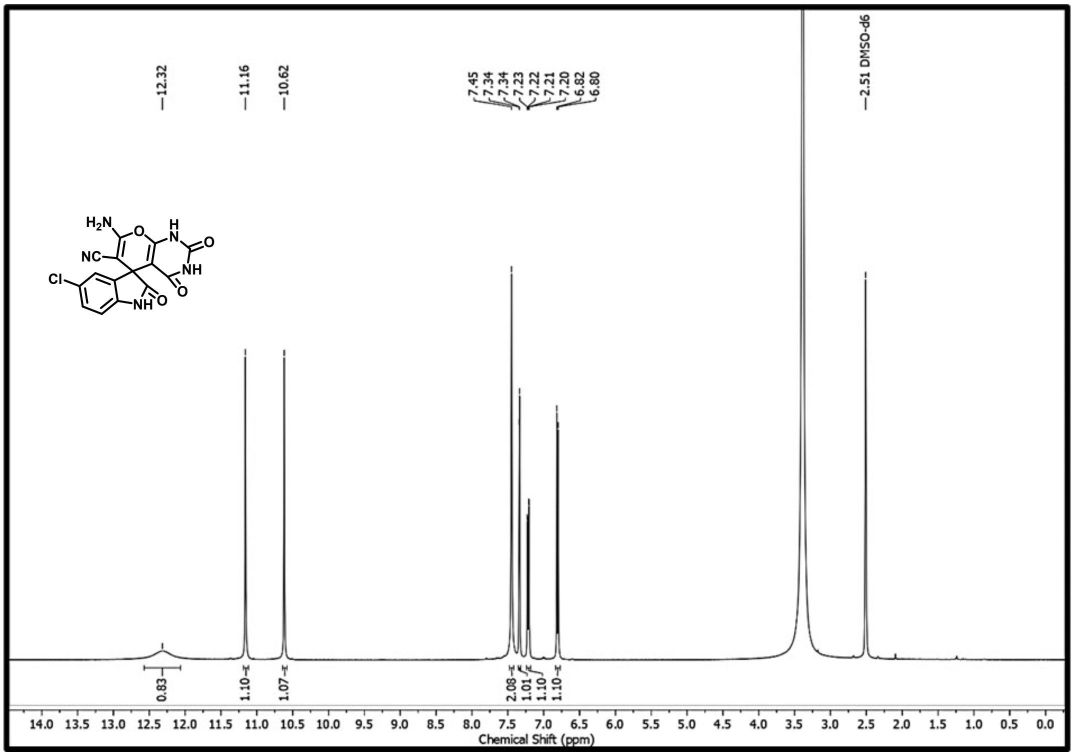

Fig. S45. ^{1}H NMR of (±)-7'-Amino-5-chloro-2,2',4'-trioxo-1',2',3',4'-tetrahydrospiro[indoline-3,5'-pyrano[2,3-*d*]pyrimidine]-6'-carbonitrile (2h).

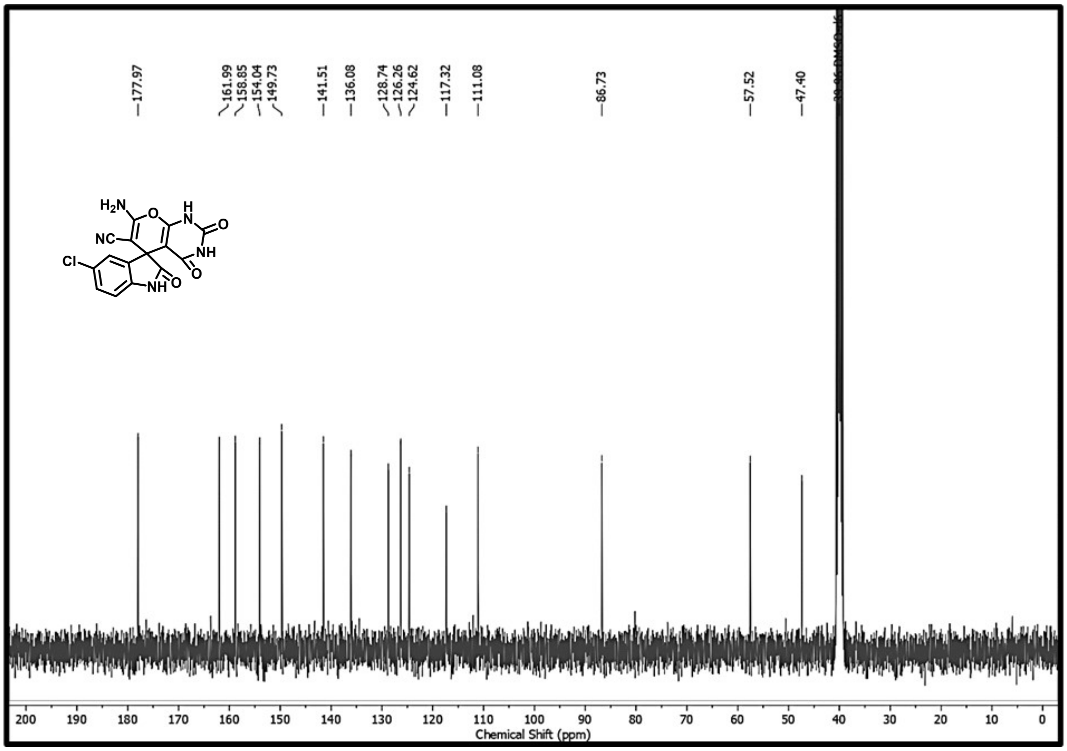

Fig. S46. ^{13}C NMR of (±)-7'-Amino-5-chloro-2,2',4'-trioxo-1',2',3',4'-tetrahydrospiro[indoline-3,5'-pyrano[2,3-*d*]pyrimidine]-6'-carbonitrile (2h).

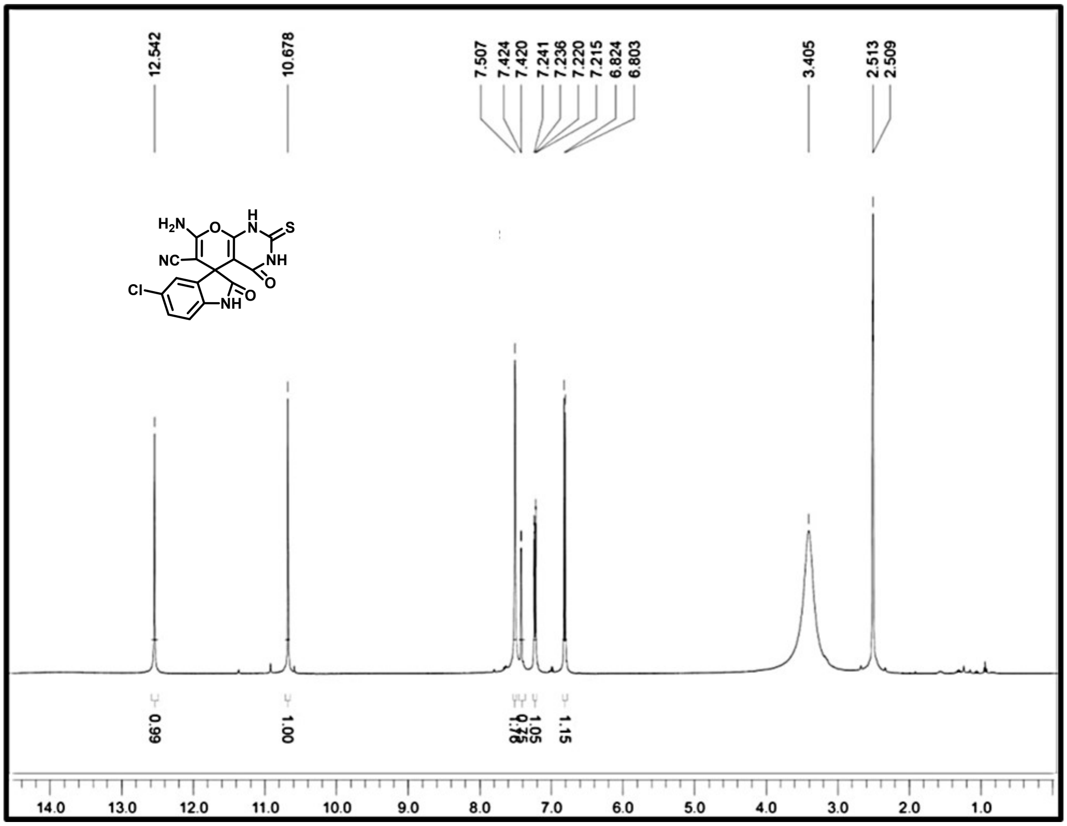

Fig. S47. ^{1}H NMR of (±)-7'-Amino-5-chloro-2,4'-dioxo-2'-thioxo-1',2',3',4'-tetrahydrospiro[indoline-3,5'-pyrano[2,3-*d*] pyrimidine]-6'-carbonitrile (2i).

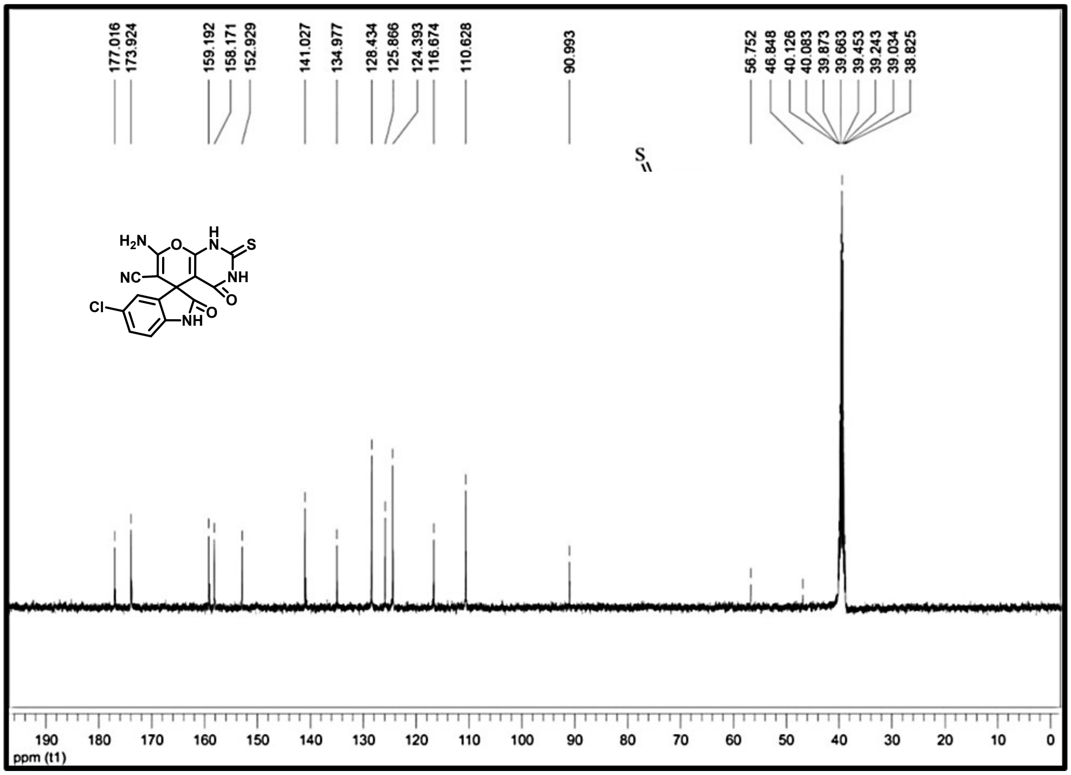

Fig. S48. ^{13}C NMR of (±)-7'-Amino-5-chloro-2,4'-dioxo-2'-thioxo-1',2',3',4'-tetrahydrospiro[indoline-3,5'-pyrano[2,3-*d*] pyrimidine]-6'-carbonitrile (2i).

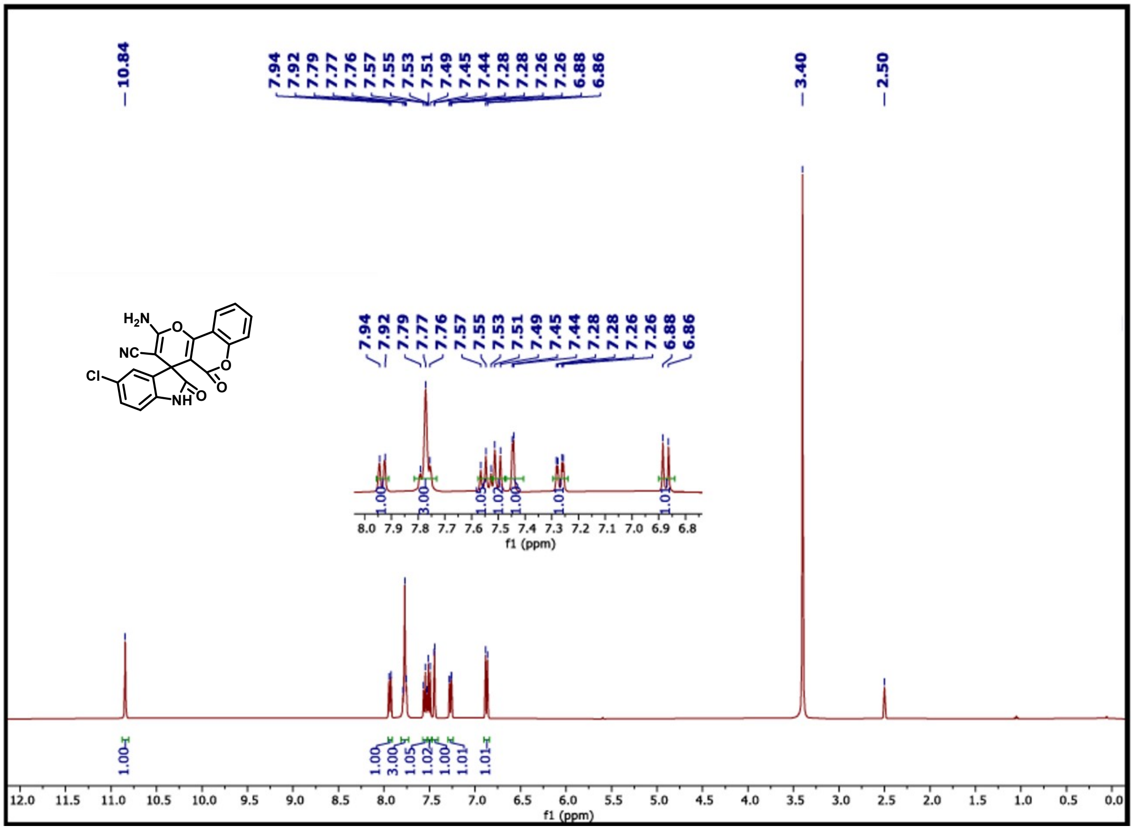

Fig. S49. ^{1}H NMR of (±)-2'-Amino-5-chloro-2,5'-dioxo-5'*H*-spiro[indoline-3,4'-pyrano[3,2-*c*]chromene]-3' carbonitrile (2j).

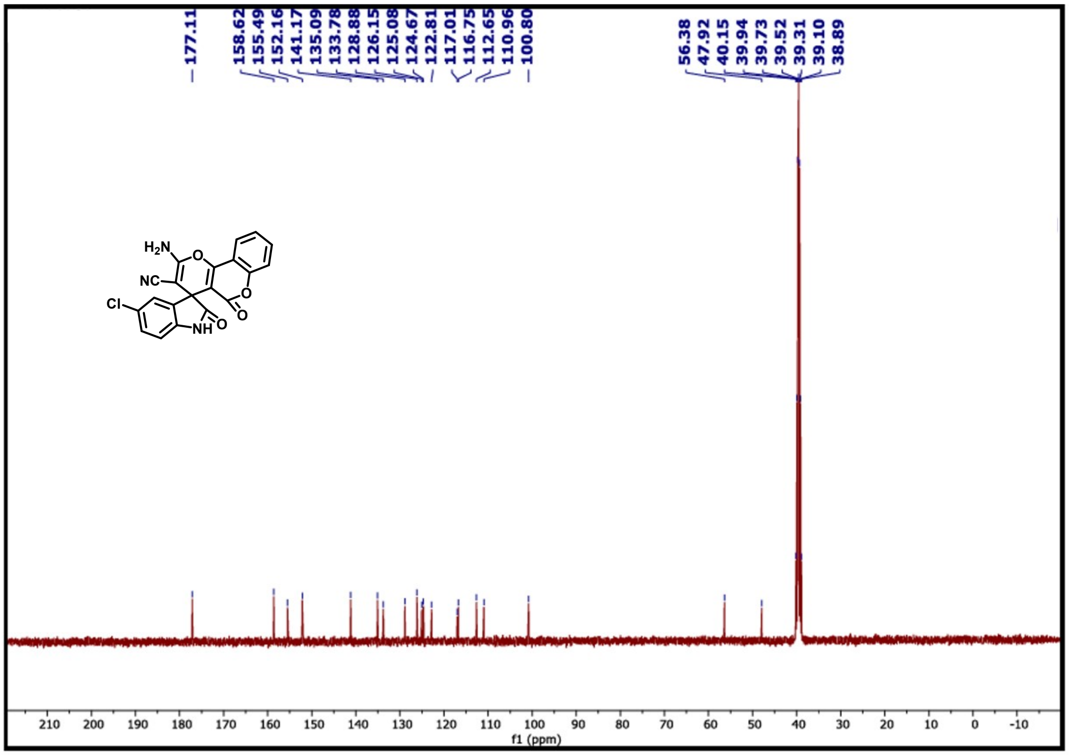

Fig. S50. ^{13}C NMR of (±)-2'-Amino-5-chloro-2,5'-dioxo-5'*H*-spiro[indoline-3,4'-pyrano[3,2-*c*]chromene]-3'-carbonitrile (2j).

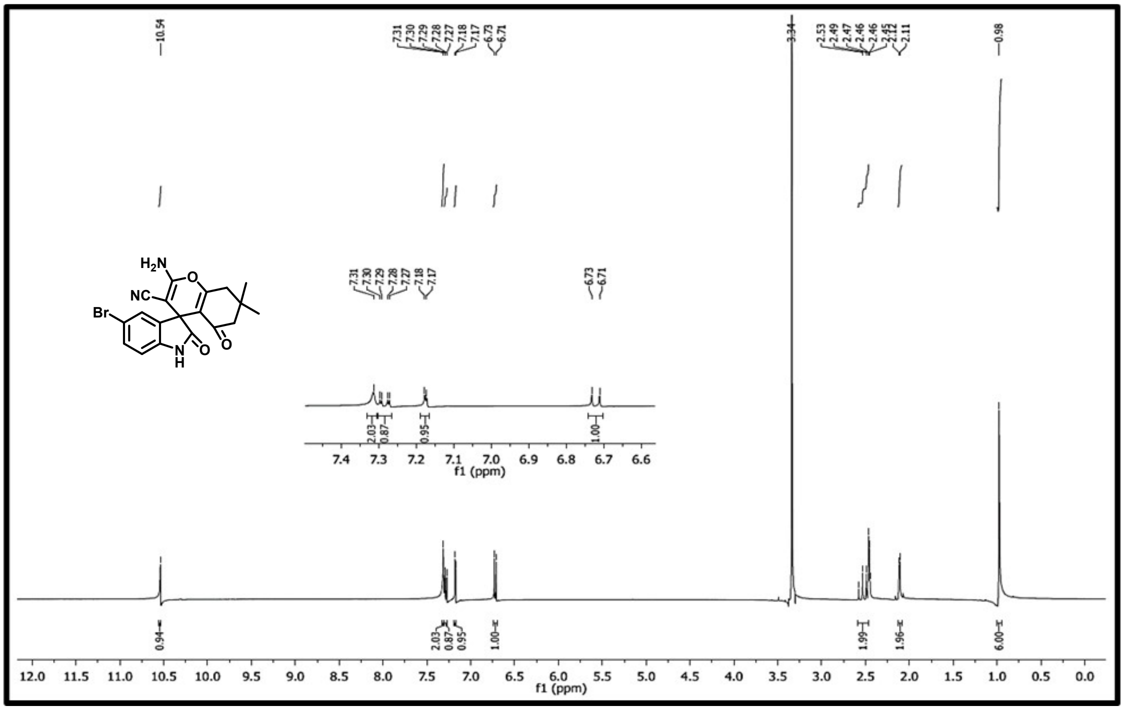

Fig. S51. ^{1}H NMR of (±)-2-Amino-5'-bromo-7,7-dimethyl-2',5-dioxo-5,6,7,8-tetrahydrospiro[chromene-4,3'-indoline]-3-carbonitrile (2k).

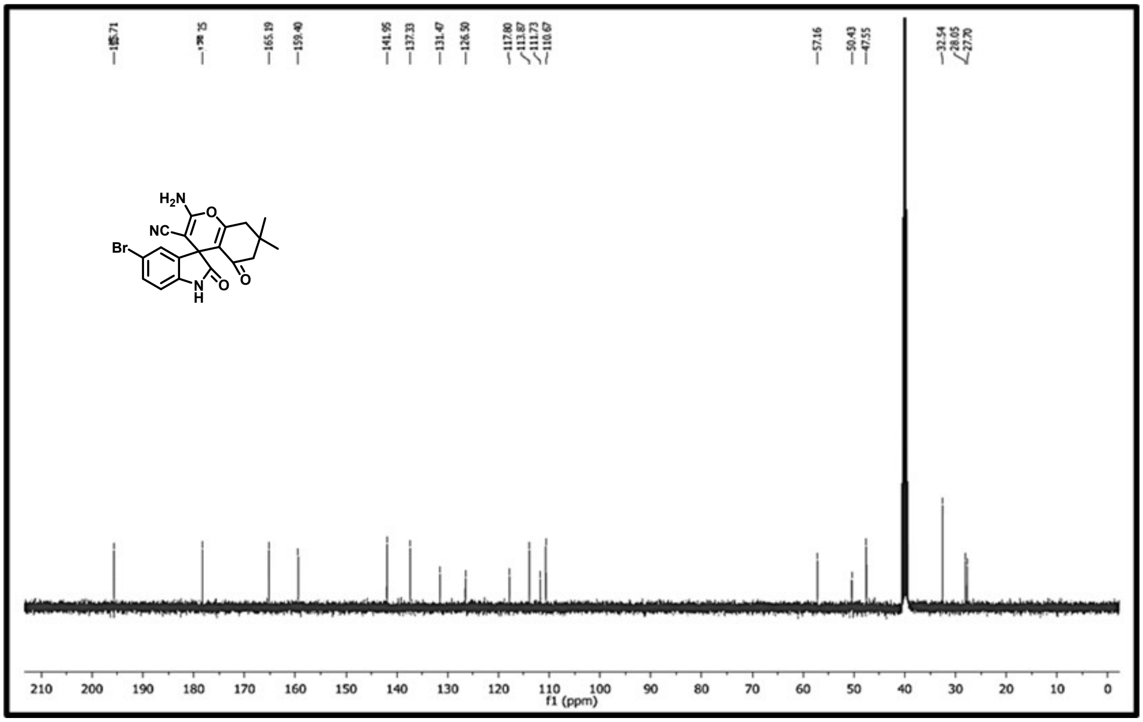

Fig. S52. ^{13}C NMR of (±)-2-Amino-5'-bromo-7,7-dimethyl-2',5-dioxo-5,6,7,8-tetrahydrospiro[chromene-4,3'-indoline]-3-carbonitrile (2k).

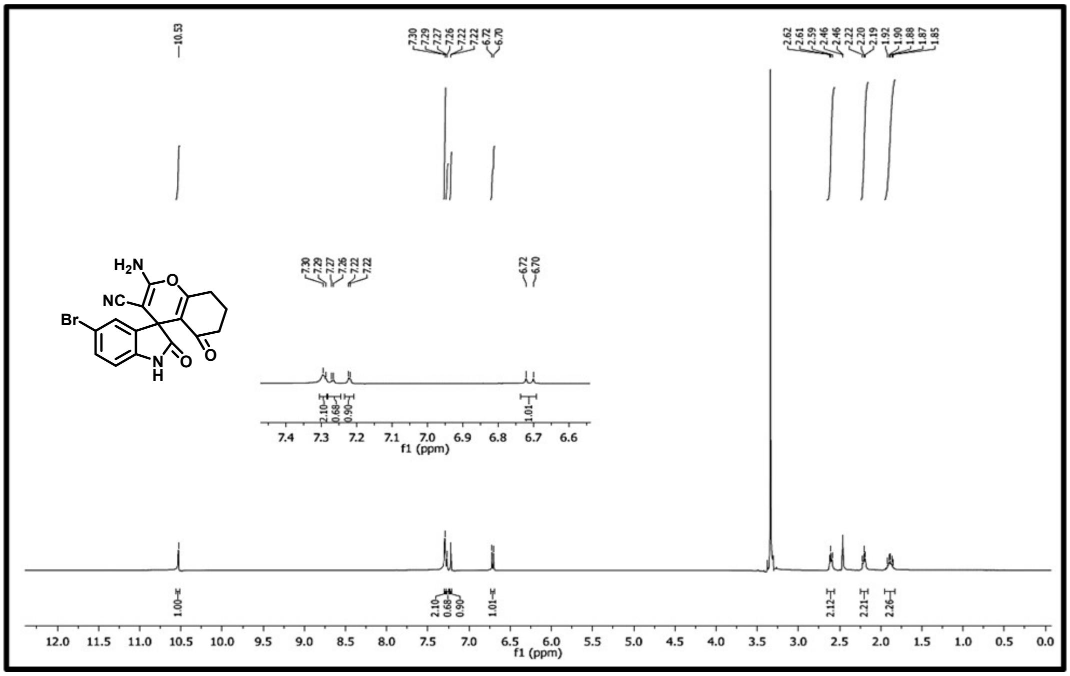

Fig. S53. ^{1}H NMR of (±)-2-Amino-5'-bromo-2',5-dioxo-5,6,7,8-tetrahydrospiro[chromene-4,3'-indoline]-3-carbonitrile (2l).

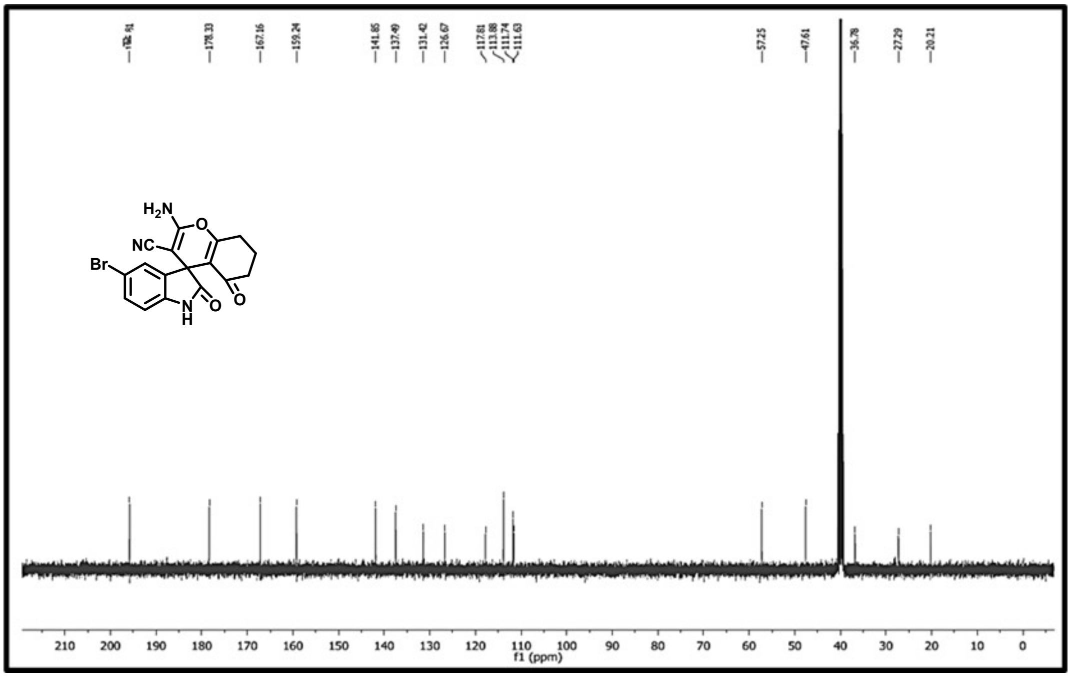

Fig. S54. ^{13}C NMR of (±)-2-Amino-5'-bromo-2',5-dioxo-5,6,7,8-tetrahydrospiro[chromene-4,3'-indoline]-3-carbonitrile (2l).

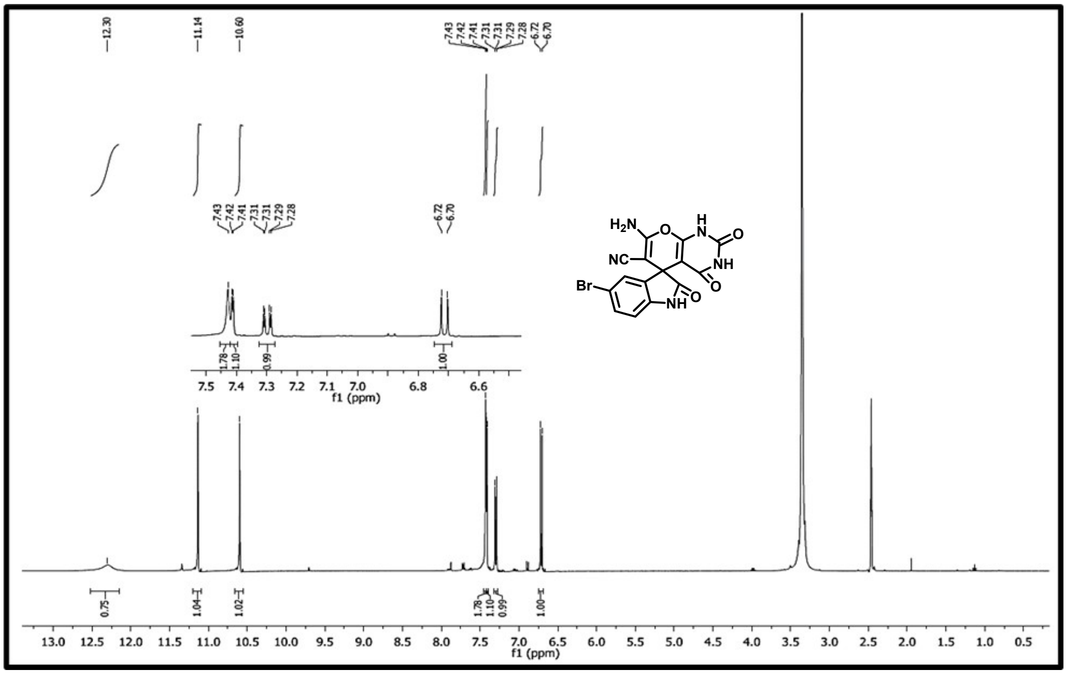

Fig. S55. ^{1}H NMR of (±)-7'-Amino-5-bromo-2,2',4'-trioxo-1',2',3',4'-tetrahydrospiro[indoline-3,5'-pyrano[2,3-*d*]pyrimidine]-6'-carbonitrile (2m).

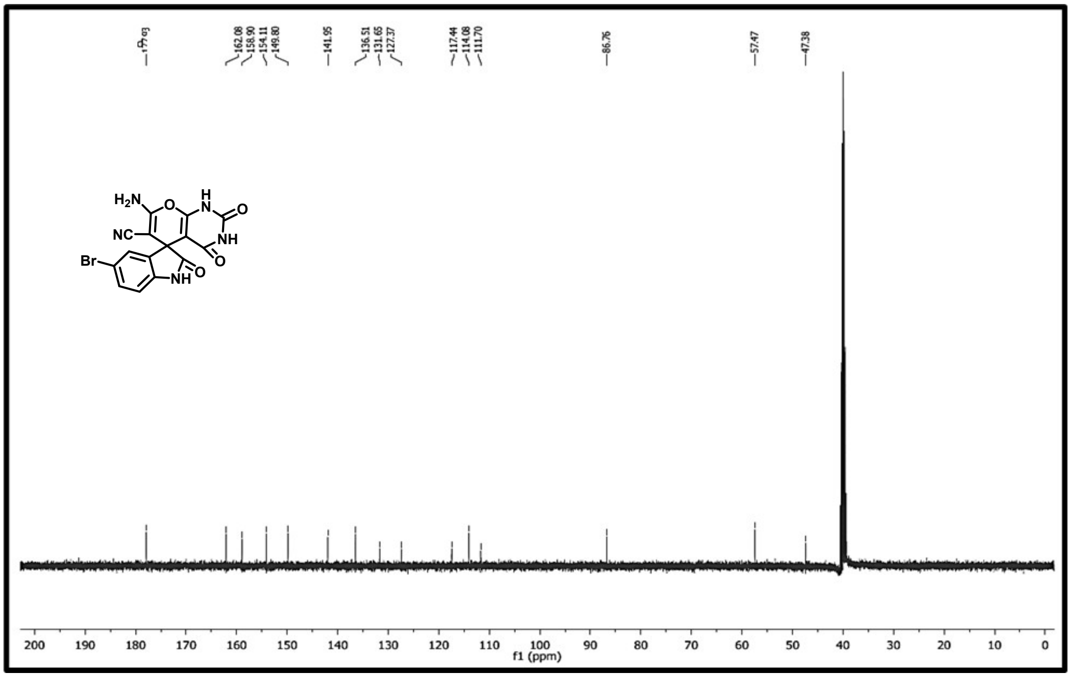

Fig. S56. ^{13}C NMR of (±)-7'-Amino-5-bromo-2,2',4'-trioxo-1',2',3',4'-tetrahydrospiro[indoline-3,5'-pyrano[2,3-*d*]pyrimidine]-6'-carbonitrile (2m).

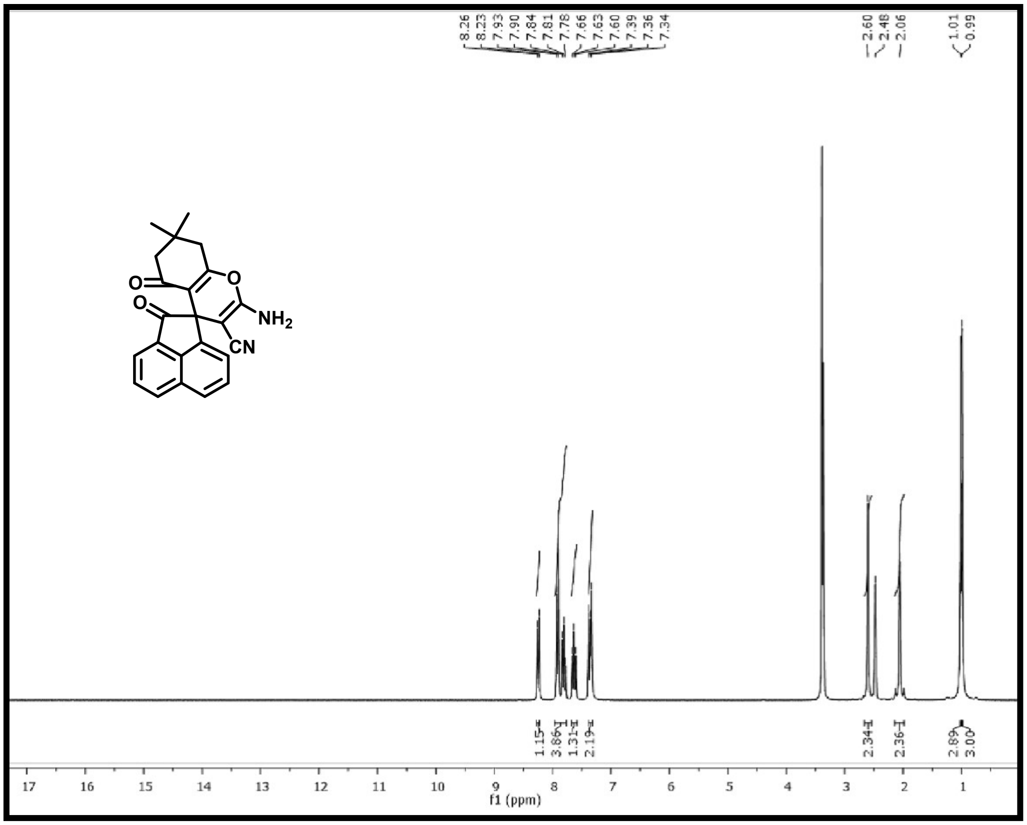

Fig. S57. ^{1}H NMR of (±)-2'-Amino-2,5-dioxo-6,8-diamino-2,5'dihydro-2*H*-spiro[acenaphthylene-1,4'-chrom-ene]-3'-carbonitrile (2n).

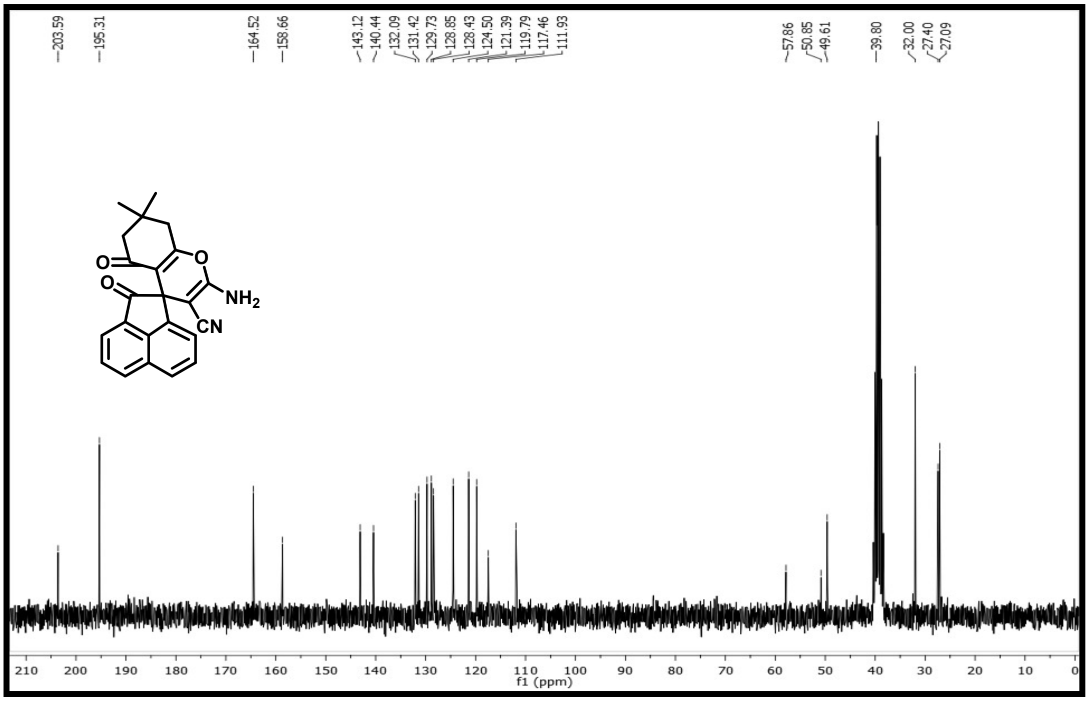

Fig. S58. ^{13}C NMR of (±)-2'-Amino-2,5-dioxo-6,8-diamino-2,5'dihydro-2*H*-spiro[acenaphthylene-1,4'-chrom-ene]-3'-carbonitrile (2n).

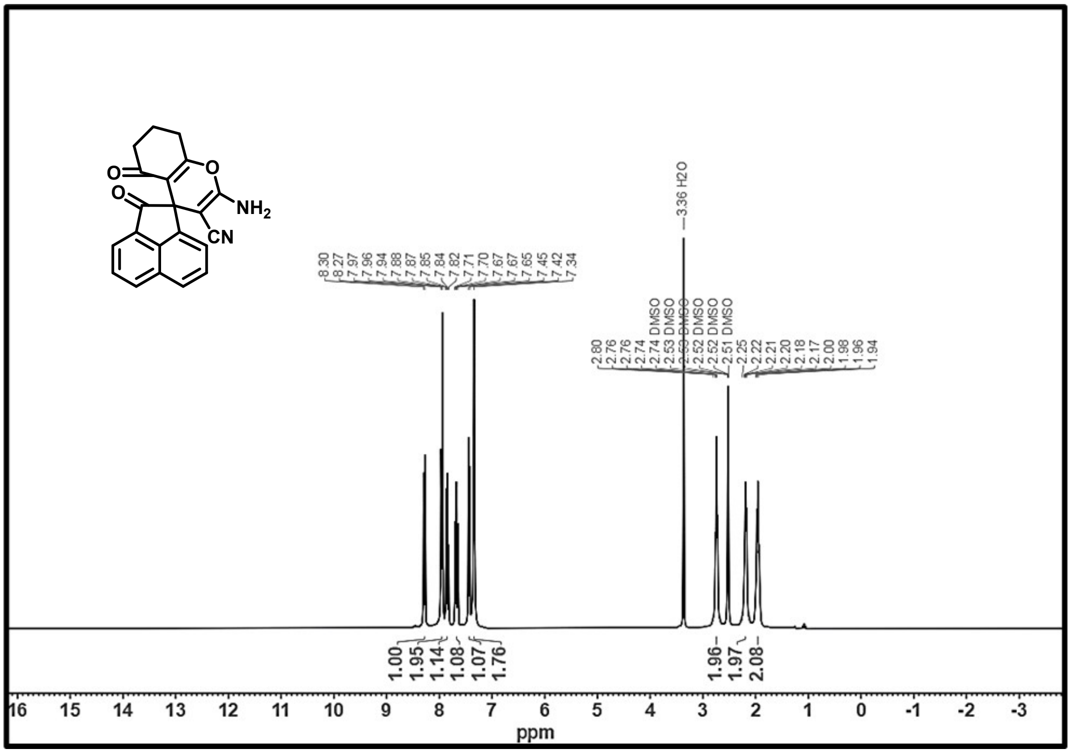

Fig. S59. ^{1}H NMR of (±)-2'-Amino-2,5'-dioxo-5',6',7',8'-tetrahydro-2*H*-spiro[acenaphthylene-1,4'-chromene]-3'-carbonitrile (2o).

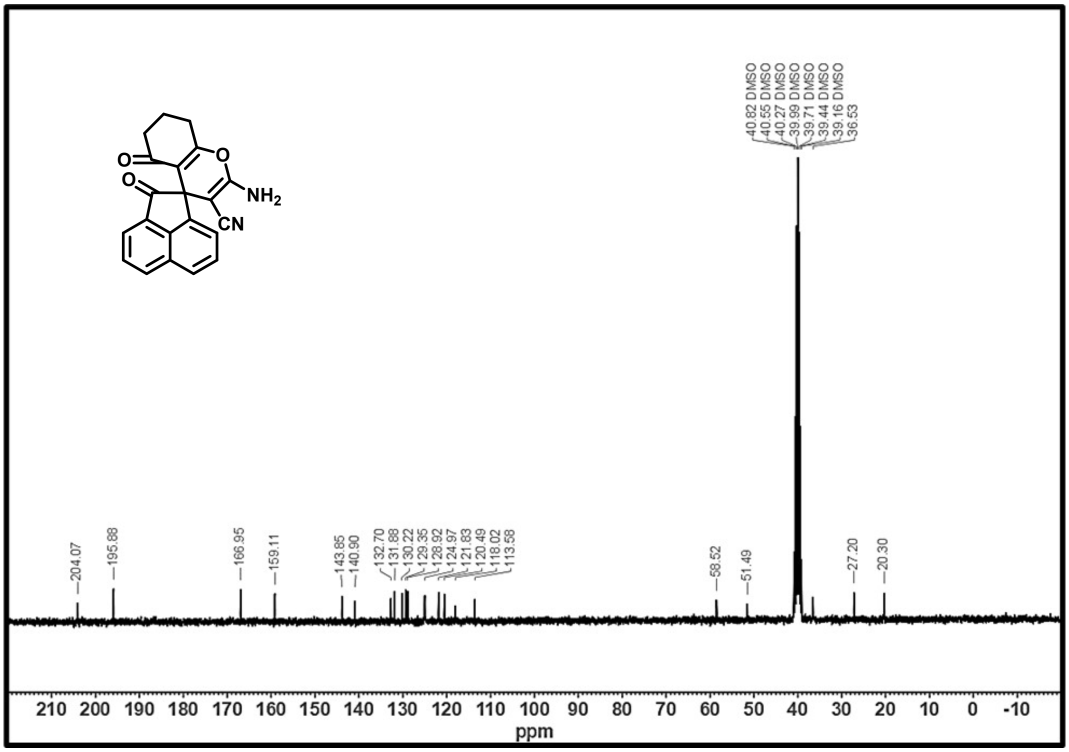

Fig. S60. ^{13}C NMR of (±)-2'-Amino-2,5'-dioxo-5',6',7',8'-tetrahydro-2*H*-spiro[acenaphthylene-1,4'-chromene]-3'-carbonitrile (2o).

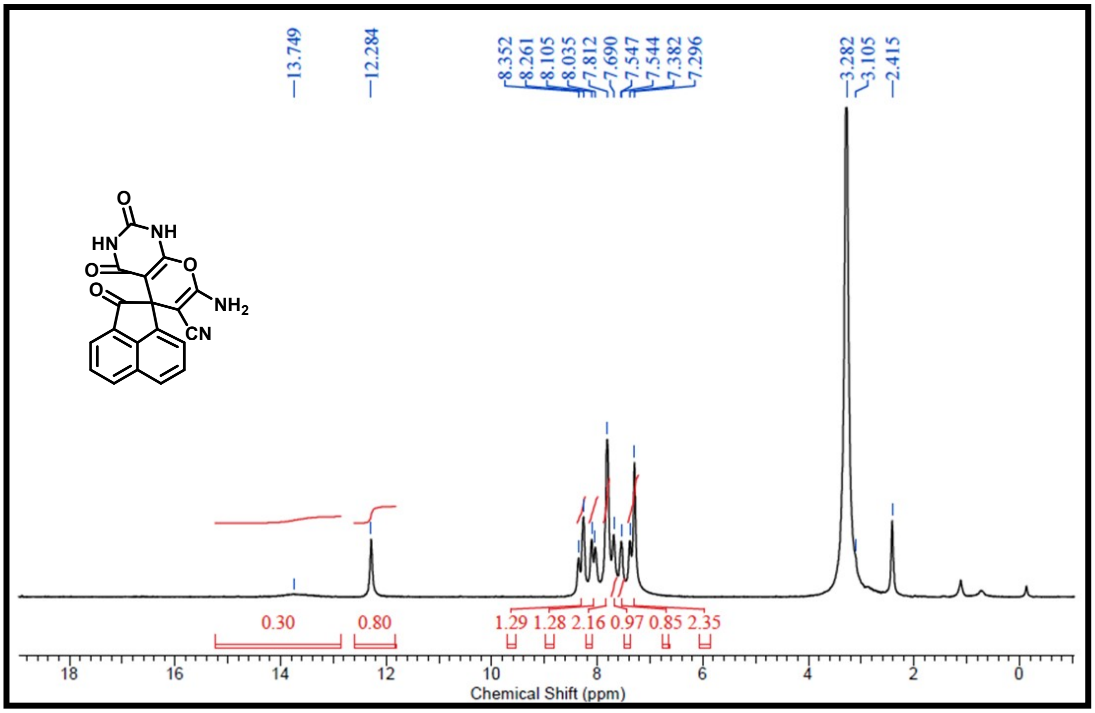

Fig. S61. ^{1}H NMR of (±)-2'-Amino-2,5'-dioxo-2,5'dihydro-2*H*-spiro(acenaphthylene-1,4'-pyrano[3,2*c*]chromene)-3'-carbonitrile (2p).

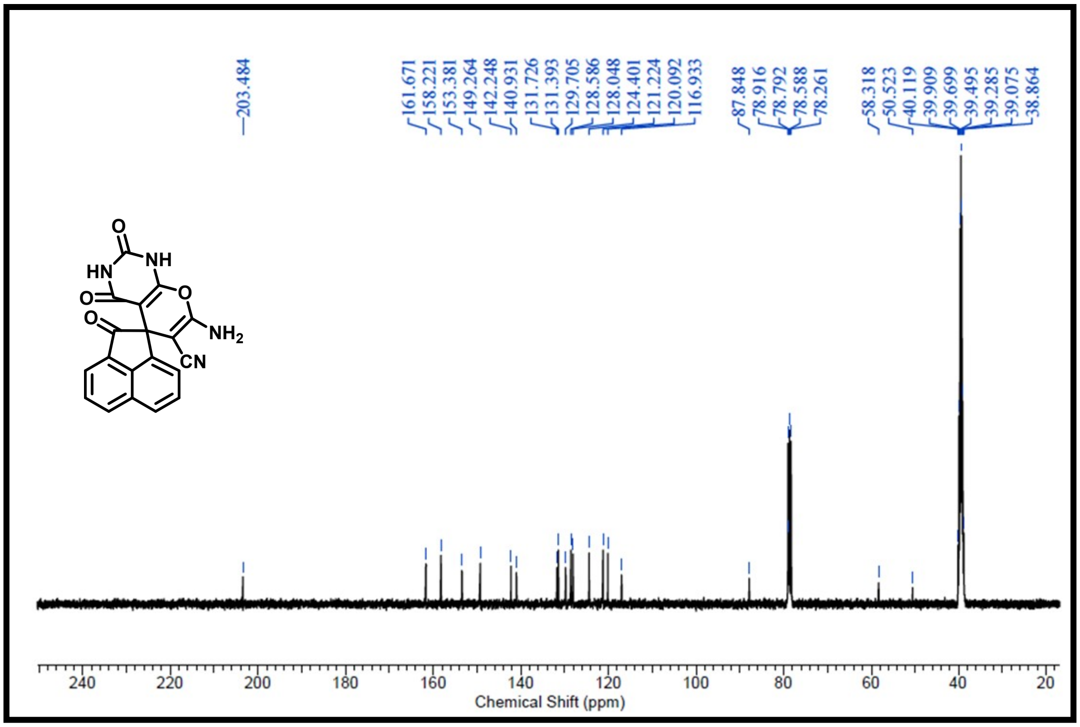

Fig. S62. ^{13}C NMR of (±)-2'-Amino-2,5'-dioxo-2,5'dihydro-2*H*-spiro(acenaphthylene-1,4'-pyrano[3,2*c*]chromene)-3'-carbonitrile (2p).

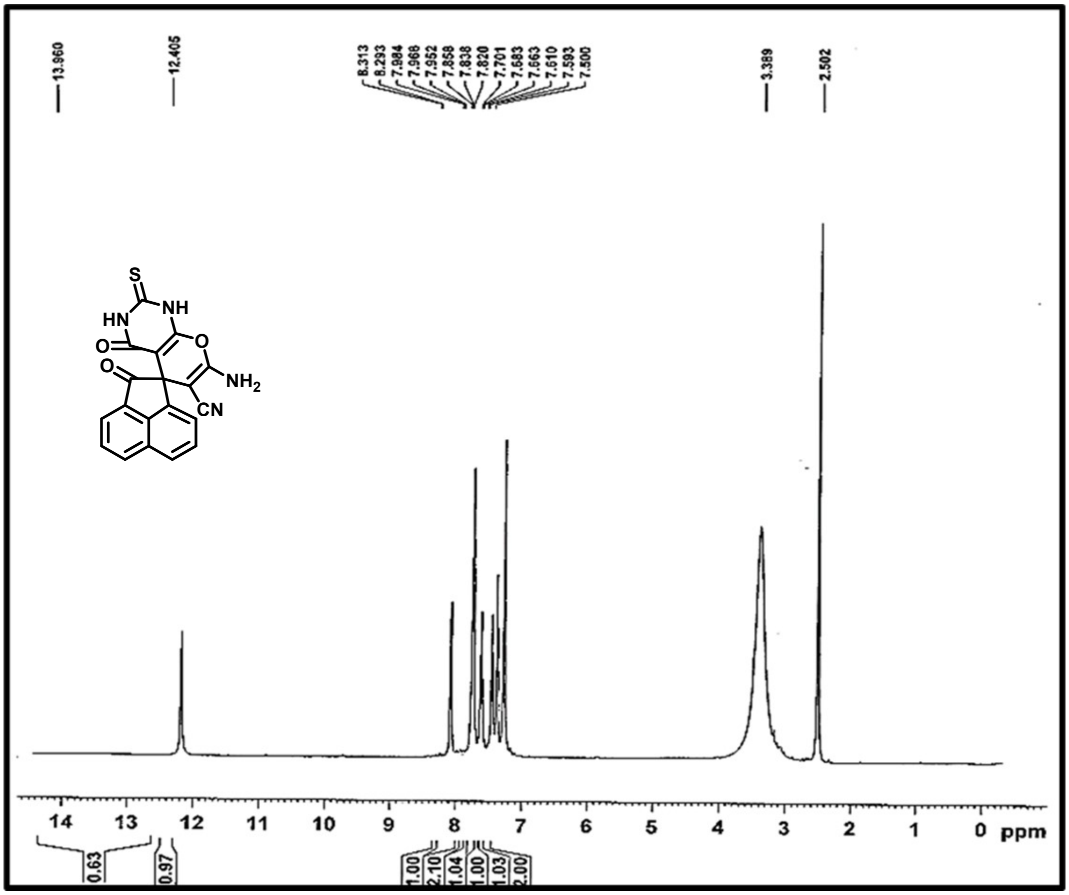

Fig. S63. ^{1}H NMR of (±)-7'-Amino-2,4'-dioxo-2'-thioxo-1',2',3',4'-tetrahydro-2*H*-spiro[acenaphthylene-1,5'-pyrano[2,3-*d*]pyrimidine]-6'-carbonitrile (2q).

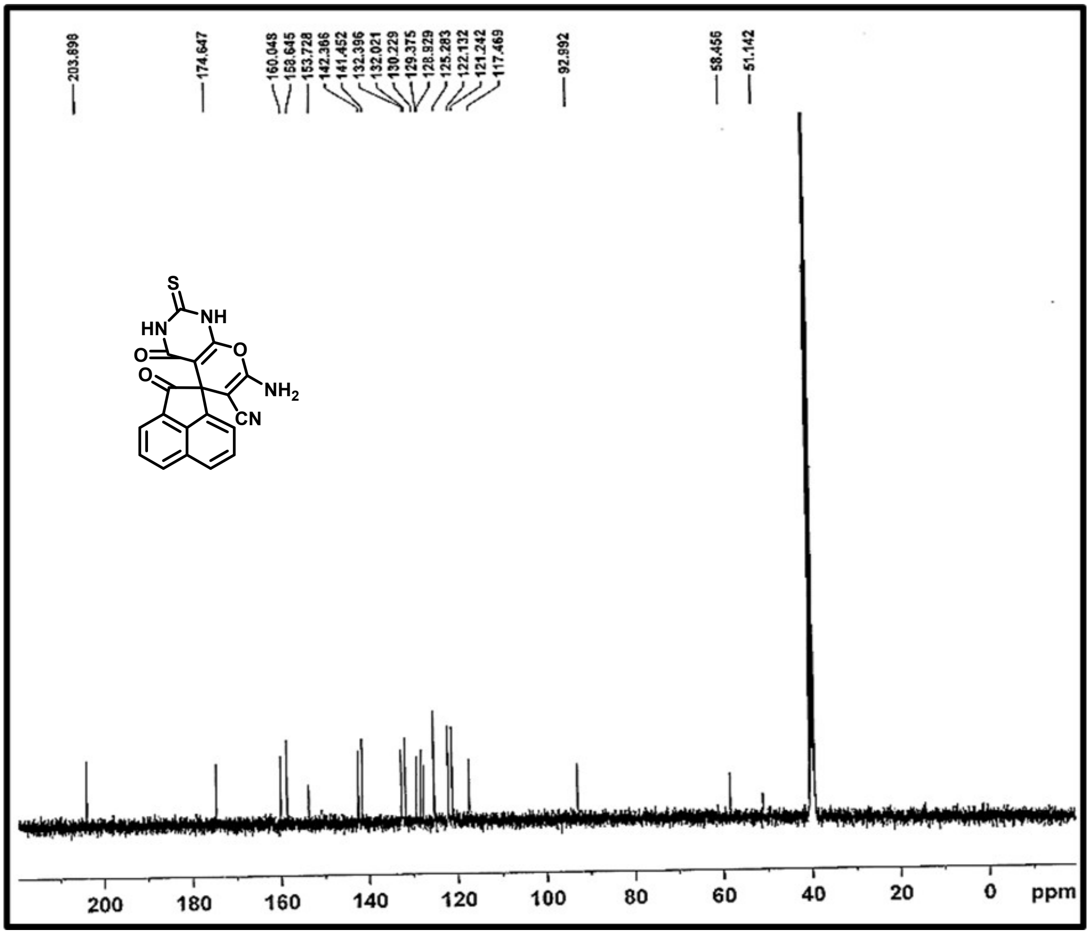

Fig. S64. ^{13}C NMR of (±)-7'-Amino-2,4'-dioxo-2'-thioxo-1',2',3',4'-tetrahydro-2*H*-spiro[acenaphthylene-1,5'-pyrano[2,3-*d*]pyrimidine]-6'-carbonitrile (2q).

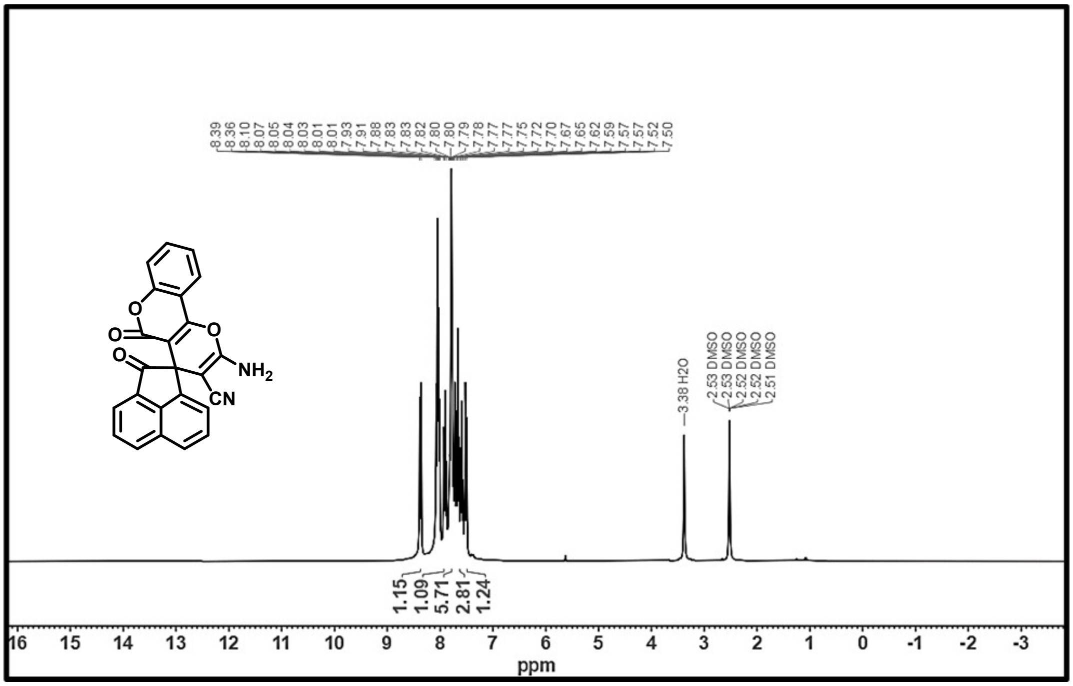

Fig. S65. ^{1}H NMR of (±)-2'-Amino-2,5'-dioxo-2*H*,5'*H*-spiro[acenaphthylene-1,4'-pyrano[3,2-*c*]chromene]-3'-carbonitrile (2r).

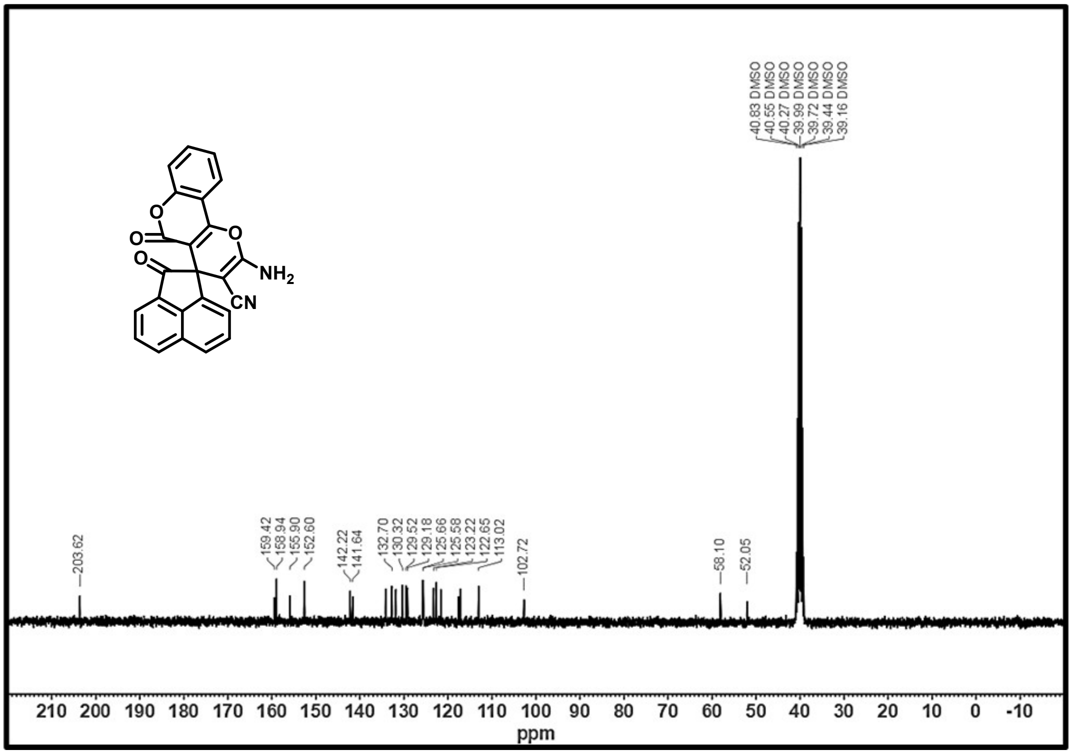

Fig. S66. ^{13}C NMR of (±)-2'-Amino-2,5'-dioxo-2*H*,5'*H*-spiro[acenaphthylene-1,4'-pyrano[3,2-*c*]chromene]-3'-carbonitrile (2r).
